# Comparison of methods to handle missing values in a binary index test in a diagnostic accuracy study – a simulation study

**DOI:** 10.1186/s12874-026-02865-6

**Published:** 2026-04-30

**Authors:** Dennis Juljugin, Katharina Stahlmann, Antonia Zapf

**Affiliations:** Institute of Medical Biometry and Epidemiology, University Medical Center Hamburg-Eppendorf, Hamburg, Germany

**Keywords:** Missing values, Diagnostic study, Simulation study, Sensitivity, Specificity

## Abstract

**Background:**

As there are no recommendations on handling missing values in a dichotomous index test of a diagnostic study, researchers often ignore missing values in the analysis or use simple methods. Thus, this simulation study compares selected methods regarding their performance of estimating sensitivity and specificity of a dichotomous index test with missing values.

**Methods:**

Data of a single-test diagnostic study were simulated including a dichotomous reference standard, a dichotomous index test and three dichotomous covariates. Following different proportions of missing values and missingness mechanisms, missing values were modeled in the index test. Additionally, the sample size, true sensitivity and specificity, and the prevalence of the target condition were varied in the data generation resulting in 729 scenarios. Seven methods were compared: complete case analysis, worst case scenario (WC), random hot deck, multiple imputation by chained equations (MICE), and three different product multinomial framework approaches.

**Results:**

Apart from WC, most methods are unbiased under missing completely at random (MCAR). Under missing at random (MAR), however, MICE clearly outperforms the other methods and is nearly unbiased while the other methods are considerably more biased. Additionally, MICE shows the best coverage probability for MCAR and MAR. If missing values are missing not at random (MNAR), all methods are substantially biased and show coverage probability that is too low.

**Conclusions:**

While most methods perform well when the proportion of missing values is small, especially under MCAR, MICE should be used when the proportion of missing values increases and the missing values are MAR. None of the tested methods seems to be suitable for MNAR.

## Background

Missing data is an ever-present issue in medical research. When collecting data in the real-world, missing values are often unavoidable even if preventative measures were taken [1]. The analysis of incomplete data sets poses a risk of a lack of validity and efficiency of the results [2], and it is therefore important to have certainty on how to handle missing data.

In the field of medical research, intervention studies have seen the most amount of attention when it comes to testing methods for handling missing data and deriving guidelines. One type of study that has received less attention regarding research on missing data is diagnostic studies. This leads to researchers conducting diagnostic studies often only considering complete cases [3, 4]. However, the Standards for Reporting of Diagnostic Accuracy Studies (STARD) guidelines clearly recommend a more sophisticated approach in the face of incomplete data, which might include assessing the impact of the missing data or imputing the missing data, to avoid bias and loss of precision [2]. This seeming lack of awareness of productive ways to approach missing data in diagnostic studies, aside from complete case analyses, motivated Stahlmann et al. [5] to conduct a scoping review of methods of handling missing data. The scoping review showed that there is not necessarily a lack of methods, but rather that finding these methods and using them can be quite work- and time-intensive. Additionally, the authors offered some advice on how to implement these methods in practice, without being able to provide concrete recommendations as a systematic comparison of methods was lacking [5].

In order to conduct a systematic comparison of the methods outlined in the scoping review, it is first important to note that diagnostic studies can be designed in different ways and as such, missing data recommendations might differ by design. Conventionally, diagnostic tests that are examined in diagnostic studies have dichotomous test results and thus, the sensitivity (probability of a positive result given that a patient has the target condition) and specificity (probability of a negative result given that a patient does not have the target condition) of these tests should be investigated as co-primary endpoints. If diagnostic tests have ordinal or continuous outcomes, sensitivity, and specificity values can be assessed for each possible threshold and represented in a receiver operating characteristics (ROC) curve and by the area under the ROC curve (AUC). The overview of methods by Stahlmann et al. [5] clearly indicates that these two cases require different sets of methods of handling missing data. Furthermore, Stahlmann et al. [6] have already compared methods with regard to their performance in estimating the AUC of a continuous index test with missing values. However, research regarding methods for estimating sensitivity and specificity for a binary index test with missing values is still lacking. This study aims to fill this gap.

Missing data can occur in the index test (the new diagnostic test that is evaluated in the study), the reference test (the test that indicates the patients’ true target conditions), or the covariates that are recorded alongside the test results. Considering that this study is motivated by an initial examination of methods, only missings in the index test will be considered here. Also, different mechanisms can underlie the missingness itself. Data can be missing completely at random (MCAR), missing at random (MAR) or missing not at random (MNAR) [7].

A systematic comparison to assess the performance of methods of handling missing data for diagnostic studies in different scenarios is a complicated issue that does not necessarily allow for analytical approaches to provide definitive answers. Where analytic results are hard to achieve, simulation studies offer help by providing empirical results [8, 9]. Therefore, this paper will examine the missing data methods via a simulation study.

To summarize, the handling of missing data is an issue that has not received much attention in the context of diagnostic studies. This study aims to compare different methods in regard to their performance in accurate estimations of sensitivity and specificity for a binary index test with missing values.

This paper is structured as follows: in Methods section the methods of this study will be presented following the ADEMP structure [9]. In Results section the results of the simulation study will be shown. In Discussion section, the results will be discussed as well as the limitations of this study and prospects for future research.

## Methods

Simulation studies are empirical experiments and as such it is important to plan the study rigorously. Morris et al. [9] recommend a planning scheme that is structured in the following way: Aims, Data-generating mechanisms, Estimands and other targets, Methods, and Performance measures. This planning scheme is referred to as the ADEMP structure. This section will abide by the ADEMP structure to report the methods of the simulation study at hand.

### Preliminary information

This simulation study was conducted in R version 4.4.1 [10] and RStudio Version 2023.12.0.369 [11]. All the code needed to run the simulation and calculate the results can be found in our GitHub repository [12].

The simulation was run on a computer with an AMD Ryzen 5 5600 6-Core Processor and 16 GB of RAM. The simulation time that will be presented in the Results section reflects the hardware and other hardware will differ in simulation time.

### Aims

This simulation study aims to assess the performance of different missing data handling methods in situations where sensitivity and specificity are estimated for a binary index test with missing values as co-primary endpoints in a diagnostic study. The performance quality of the different missing data handling methods is directly tied to the quality of the pseudo-complete data sensitivity and specificity estimates. Ideally, the estimates should not be biased and $95\%$ of confidence intervals based on these estimates should contain the true sensitivity or specificity value.

### Data-generating mechanism

As previously mentioned, in diagnostic studies with sensitivity and specificity as co-primary endpoints, test outcomes for the index as well as the reference test are in general dichotomous. Moreover, most of the missing data handling methods studied, that will be introduced in a following subsection, perform best or only when the data set includes correlated covariates. Some methods only work when the covariates are categorical, which is why three dichotomous covariates are simulated alongside the index test results. Thus, this corresponds to a cross-sectional diagnostic study following a single-test design with an error-free reference standard, some patient characteristics (covariates) and a binary index test with missing values.

To simulate correlated dichotomous variables it is recommended to simulate continuous data through a multivariate normal model and dichotomize the continuous data accordingly [8, 9]. Thus, two different multivariate normal models were used to generate data for both reference standard groups with the function rmvnorm from the mvtnorm package [13]. The multivariate normal models include continuous index test values and three continuous covariates that are correlated to the index test values. All four variables were dichotomized after combining the simulated data for both reference standard outcome groups. These two models solely differ in respect to the means of the pre-dichotomization index test data. While the population without the target condition had a mean of 0, the population with the target condition had a mean of $\Phi ^{-1}(True\; Specificity) - \Phi ^{-1}(1 - True\; Sensitivity)$ where $\Phi ^{-1}$ represents the inverse cumulative distribution function of the normal distribution. The covariates had means of 0, 5, and 35 and may serve as an example for sex, a biomarker (dichotomized) and age group. To dichotomize the initially continuous data for the index test, a cut-off value of $\Phi ^{-1}(True\; Specificity)$ was specified to ensure that the complete-data sensitivity and specificity values are on average equal to the true sensitivity and specificity (for a derivation of this dichotomization method please see the supplementary material). The remaining covariates were dichotomized through a cut-off at their respective means. MacCallum et al. [14] detail how the correlation coefficient for continuous variables that are dichotomized can be adjusted appropriately, which was done here in order to preserve a correlation of 0.4. This is only a nuisance in this case but might prove to be useful in further research where the effect of different correlation sizes is investigated. More detailed information the data generation, particularly on the derivation of the mean of the index test in the group with the target condition and the dichotomization of the index test can be found in the supplementary material.

In order to simulate missing data for the index test result, the function ampute from the mice R package [15] was used. Among other things, ampute allows for the specification of the missing proportion and of the missingness mechanism (MCAR, MAR, MNAR). In the case of MCAR, every data point has the same probability of being simulated as missing. For MAR and MNAR, the missingness probability of any given data point is dependent on a weighted sum score that is calculated through the covariates. For the purpose of this study, the weighted sum score can be conceptualized through

1 $$\begin{aligned} wss_i = w_1 y_{1,i} + w_2 y_{2,i} + ... + w_m y_{m,i} \end{aligned}$$

where $i$ indicates the case, as in the data row, $wss_i$ is the weighted sum score for a case $i$ and each variable $y$ is weighted by a weight $w$. In this case, the variables include the index test result, the three covariates, and the reference test result. In this study, the weight for the index test was 0 in MAR and 1 in MNAR while the weights for the covariates and the reference standard were 1 in MAR and 0 in MNAR (please see the supplementary material for more details). These are the default settings in the ampute function. The reason for this is that MNAR can be simulated through the missingness of a parameter being dependent on that parameter itself. In contrast, missingness in the index test is dependent on other oberseved variables under MAR. The article by Schouten et al. [16] details the weighted sum score approach in ampute. The ampute function also includes a further parameter $k$ for missingness pattern. This is a nuisance here as there is only one missingness pattern, i.e. only one combination of variables with missing and complete data which is missing values in the index test and completely observed covariates and reference standard. As not all methods compared in this simulation study can handle missing values in more than one variable and as the focus of this article is on missing values in the index test, we decided on this missingness pattern. In reality, missing values may occur in more than one variable (e.g. also in covariates or the reference stadnard). Future research should examine the case of missing values in the index test and covariates and/or the reference standard.

It was already mentioned that the simulated scenarios had varying sensitivity and specificity values, as well as varying missingness proportions and missingness mechanisms. Table 1 showcases all the possible values for all parameters that were varied in the simulation study. We decided on varying these parameters and their values to cover a broad range of scenarios including more realistic scenarios (e.g. proportion of missing values of 0.1 or 0.3 and a prevalence of 0.3) as well as more extreme scenarios (e.g. a proportion of missing values of 0.5 with a prevalence of 0.1).

**Table 1:** Simulation study scenarios

Scenario	Value
Sensitivity	0.7, 0.8, 0.9
Specificity	0.7, 0.8, 0.9
Missingness mechanism	MCAR, MAR, MNAR
Proportion of missings	0.1, 0.3, 0.5
Prevalence of the target condition	0.1, 0.2, 0.4
Sample size	400, 800, 1600

Each condition was varied factorially in the data generation, resulting in $3^6 = 729$ scenarios per imputation method. For each method, there were 1,000 simulations per condition, so 729,000 simulations in total per method.

### Estimands

As already mentioned, the goal is to estimate sensitivity and specificity of the index test through the pseudo-complete data set with their $95\%$ logit confidence intervals.

### Methods in the simulation study

#### Complete case analysis

The Complete Case Analysis (CCA) only considers complete cases. This method is very easy to implement and equivalently popular, but wrongfully so: Only when data is missing completely at random, CCA can ensure unbiased estimates [17]. Even when unbiased, CCA under MCAR suffers from power loss, as the sample size is decreased due to the missing data. Once the missing mechanism is not negligible (MAR or MNAR), CCA is expected to yield biased estimates. Consequently, it is usually advised not to use CCA [18].

#### Worst case scenario

The worst case method (WC), associated with the intention-to-diagnose principle [3], is easily applicable for dichotomous outcomes. Each missing value is treated either as a false-positive or false-negative, depending on the true target condition. It can be expected that the worst case method yields biased results. What the method excels at, however, is determining a lower bound for the estimates.

#### Random hot deck

Random Hot Deck imputation is a method that is very common in, among other cases, survey data [19]. It works by imputing missing values with matching observed values. The observations with the missing value(s) are usually referred to as the recipient, while the observations with the observed data are referred to as the donor. In Random Hot Deck imputation, donors for each recipient are chosen randomly.

One clear advantage of the Random Hot Deck method is that only already observed, and thus plausible, data is used to impute [19]. This is, however, a negligible advantage in the case of dichotomous data, as plausible dichotomous outcomes are essentially predetermined. A disadvantage of the method is that the theory behind it is not very well-developed, and there are no clear guidelines as to when this method should be used. Nevertheless, the Random Hot Deck method is frequently used in practice and therefore, an inclusion into a simulation study to explore its properties should be more than welcome.

In this simulation study, the imputation of missing values with randomly chosen observed values were performed stratified by reference standard group. That is to say, the dataset was split into two subsets according to the reference standard result (one subset for the subjects with and one subset for the subjects without the target condition) and donors for the recipients were chosen randomly in the subsets. After this single imputation, the two subsets were combined to one dataset for further analysis. The Random Hot Deck methods allow for more sophisticated donor election procedures via various distance metrics [19], for example, but this will be a subject of discussion later in this paper.

To implement Random Hot Deck into R, the hotdeck function from the VIM package [20] was used.

#### Multiple imputation by chained equations

##### Multiple Imputation in general

Multiple Imputation is a method for handling missing data that makes use of all possible information in the data set and is derived through Bayesian techniques [21]. It has become one of the most popular methods for handling missing data [22]. In essence, Multiple Imputation works by repeatedly randomly drawing imputation values from a distribution that is predictive of the missing values. This predictive distribution is conditional on the observed data. The number of repetitions in the Multiple Imputation procedure is often denoted by *m*, meaning that Multiple Imputation yields *m* completely imputed data sets [23]. These completely imputed data sets allow for complete data analysis methods to be applied [24]. The results of these analyses are summarized according to “Rubin’s Rules” [25] to allow for accurate p-value and confidence interval estimations. Multiple Imputation allows for more precise estimation of variance than single imputation methods. It is crucial how the model is chosen and constructed [23].

It is often recommended that the imputation model should be more general than the analysis model and must at least include all the variables of the final analysis model [26–28]. This leads to some investigators recommending that almost all variables–associated with the missingness probability and also generally in the data set–are included in the imputation model [23, 29, 30]. Still, it is advised to ensure that all included variables exceed a minimum correlation threshold [31]. Also, it is considered necessary to include the dependent variable of the analysis model in the imputation model [23, 32, 33].

The classical imputation model for the Multiple Imputation procedure as it was introduced by Rubin [21] is the multivariate normal model. While this model has a sound theoretical basis, there are some disadvantages, namely that the method introduces bias if the variables are not actually normally distributed. There is a commonly used approach to dealing with the normal method’s shortcomings: Multiple Imputations by Chained Equations (MICE).

##### Multiple Imputations by Chained Equations

Different from the multivariate normal model, the chained equations model uses a series of univariate models rather than a single large model to impute the variables [31]. In other words, the imputation is on a variable-by-variable basis. The imputation model for each variable is dependent on the variable type and scale [15]. Since diagnostic test results can be modeled via a binary variable, a logistic regression would be the appropriate imputation model choice.

In MICE, the imputation model is used to impute each variable one by one for multiple iterations using a Gibbs sampling procedure until convergence. Usually, MICE only requires a few iterations until convergence. In essence, the imputation process can be described like so: For k different random variables $X_1, X_2, ..., X_k$ where each variable may be partially observed and with iteration counter $t$, the imputation process follows the form of

$$\begin{aligned}&X_1^{t+1}\ \text {from}\ P(X_1|X_2^t, X_3^t, ..., X_k^t)\\&X_2^{t+1}\ \text {from}\ P(X_2|X_1^{t+1}, X_3^t, ..., X_k^t)\\&\vdots \\&X_k^{t+1}\ \text {from}\ P(X_k|X_1^{t+1}, X_2^{t+1}, ..., X_{k-1}^t) \end{aligned}$$

where each random variable $X_1, X_2, ..., X_k$ is imputed with the conditional probability distribution that includes the most recent data. This cycle is run until convergence is met. In the case of only missing index tests, just the first step for $X_1$ is used, as there is only one variable to impute [31].

##### Implementation in R

To implement MICE into R, the mice function from the mice package [15] was used. To function properly, the mice function needs an imputation model. A logistic regression of the following form—with $m = 5$ imputed data sets—was used as the imputation model:

2 $$\begin{aligned} P(Y=1|X_1, X_2, X_3, R) = \frac{e^{\beta _0+\beta _1X_1+\beta _2X_2+\beta _3X_3+\beta _4R}}{1+e^{\beta _0+\beta _1X_1+\beta _2X_2+\beta _3X_3+\beta _4R}} \end{aligned}$$

where $$Y = Index\;test$$, $$X_1,\; X_2,\; X_3 = Covariates$$ and $$R = Refererence\; standard$$.

With a proportion of up to 0.5 of cases missing, $m = 5$ may be slightly too few imputed data sets for robust variance estimation. Therefore, we conducted a small set of additional scenarios with 200 iterations and $m = 50$. The respective results can be found in the supplementary material. Due to a high computing time, it was not possible to use $m = 50$ for the main simulation.

#### Product multinomial framework

When considering a data set with only categorical data—such as our simulated data—four missingness pattern can be identified theoretically. For example, for two categorical variables X and Y, these patterns are: 1) The data point is observed in X and Y, 2) the data point is only observed in X, 3) the data point is only observed in Y, and 4) the data point is not observed in any variable. The latter case cannot be treated at all since there is no information available, but the remaining cases leave information that could be used for imputation. In our simulated study, only the first two patterns can occur as missing values exist in one variable (the index test) only. The Product Multinomial Framework does not consider the study data as classic data sets with different data rows per person, but rather as data tables where each cell corresponds to a full or partial classification and includes the corresponding frequency (see Table 3). An appropriate allocation of the frequencies of the partial classifications to the full classification is equivalent to data imputation.

Poleto et al. [34] propose a method to deal with partially or incompletely classified data that is based on a product multinomial framework. This method is an extension or rather generalization of the multinomial approach of dealing with this type of data by Paulino [35]. Poleto et al. [34] describe Paulino’s approach as “fitting strictly linear and log-linear multinomial models to data generated by MAR and MCAR mechanisms via maximum likelihood (ML) methods and more general functional linear models to data generated by MCAR mechanisms via weighted least squares (WLS) methodology” (p. 110). The authors’ extension of this initial model allows for more general linear and log-linear models to be applied in the maximum likelihood methodology. It is important to note here that the ML methodologies use an expectation maximization (EM) algorithm [36] for imputation.

To summarize, the product multinomial framework approach can use different methodologies. Firstly, frequencies can be allocated through a maximum likelihood approach. Whether the data is considered to be MCAR or MAR has to be specified beforehand, so these methods will be treated separately in the simulation study: maximum likelihood for MCAR (MLMCAR) and maximum likelihood for MAR (MLMAR). Additionally, the frequencies can be fit through a weighted least squares approach. This is only suitable for MCAR, which is why this method will be denoted as WLSMCAR from here.

Poleto offers an R package named ACD (formerly known as Catdata) that implements the procedure based on this method. The ACD package is currently not available in the CRAN repository but can be downloaded from Poleto’s personal website [37].

The functions satMarML and satMcarWLS impute the missing data for the maximum likelihood and weighted least squares approaches respectively, but the data needs to be transformed into a special catdata object beforehand. A catdata object specifies the frequencies for each possible category and the entries which are only partially classified. To transform a data set in the form of Table 2 into a catdata object in the form of Table 3 requires a noticeable effort that should be considered before implementing this method. Note that a catdata object is not actually a table, but rather comparable to a vector, where each vector entry corresponds to one of the table cells in Table 3.

**Table 2:** Example data set for diagnostic studies

Patient ID	Index test	Covariate	Reference test
1	1	1	1
2	1	1	1
3	0	2	1
4	NA	2	1
5	NA	1	1
6	0	2	0
7	0	2	0
8	1	1	0
9	NA	1	0
10	NA	2	0

“NA” indicates missing data

**Table 3:** Transformed data set needed for product multinomial methods

	D = 1		D = 0	
	Covariate = 1	Covariate = 2	Covariate = 1	Covariate = 2
Index test = 1	2	0	1	0
Index test = 0	1	0	0	2
Index test missing	1	1	1	1

Additionally, in the case that a category has a frequency of 0, a very small frequency, such as $10^{-6}$, needs to be substituted, otherwise the expectation maximization algorithm will not work properly and will lead to biased results [34].

Lastly, it should be stressed again that this method only works if all the data (in the imputation model) is categorical. This fact informed the data generation of this study, leading to only dichotomous covariates.

### Performance measures

Morris et al. [9] provide a list of possible performance measures for simulation studies with corresponding Monte Carlo Error estimations, a subset of which will be used to assess the performance of the methods at hand.

Since one of the main aims of the simulation study is to find an imputation method that yields unbiased sensitivity and specificity estimates, the bias is the central performance measure. Bias will be estimated through

3 $$\begin{aligned} Bias = \frac{1}{n_{sim}}\sum \limits _{i=1}^{n_{sim}}(\hat{\theta }_i - \theta ) \end{aligned}$$

where $n_{sim}$ refers to the number of simulations, $\hat{\theta }_i$ refers to the sensitivity or specificity estimate for a given simulation, and $\theta _i$ refers to the true sensitivity or specificity value.

Complementary to unbiasedness, it is necessary to establish which methods offer the most efficient estimators. This is important for establishing a selection rule if different methods are equally unbiased or biased overall or in specific scenarios. One method that combines a measure of bias and a measure of efficiency is the mean squared error (MSE):

4 $$\begin{aligned} MSE = \frac{1}{n_{sim}}\sum \limits _{i=1}^{n_{sim}}(\hat{\theta }_i - \theta )^2. \end{aligned}$$

Another measurement to assess the precision is the empirical standard error (EmpSE):

5 $$\begin{aligned} EmpSE = \sqrt{\frac{1}{n_{sim} - 1}\sum \limits _{i=1}^{n_{sim}}(\hat{\theta }_i - \bar{\theta })^2} \end{aligned}$$

where $\bar{\theta }$ is the mean of $\hat{\theta }_i$.

It is important to note here that the empirical standard error is most useful as a performance measure only in comparison to other methods. In other words, the empirical standard error is useful to find the most efficient methods among multiple ones.

Another aim of this simulation study is to explore the coverage probabilities of the different methods. The coverage probability is estimated through

6 $$\begin{aligned} Coverage = \frac{1}{n_{sim}}\sum \limits _{i=1}^{n_{sim}}{1}(\hat{\theta }_{lower,i} \le \theta \le \hat{\theta }_{upper,i}) \end{aligned}$$

where ${1}$ refers to the index function and $\hat{\theta }_{lower,i}$ and $\hat{\theta }_{upper,i}$ refer to the lower and upper bounds of a confidence interval, respectively. In this study, logit confidence intervals were used. For more information concerning the confidence interval calculation, please refer to the supplementary material. Note that the coverage can only be interpreted in relation to the bias. It is easy to see how biased estimates lead to confidence intervals with less than nominal coverage.

Lastly, for a few selected scenarios that are unbiased and have good coverage, the power will be estimated through

7 $$\begin{aligned} Power = \frac{1}{n_{sim}}\sum \limits _{i=1}^{n_{sim}}{1}(\theta _0 \le \hat{\theta }_{lower,i}) \end{aligned}$$

where $\theta _0$ corresponds to the null hypothesis sensitivity or specificity. A power analysis for methods that are biased and (thus) have less than nominal coverage is futile as low power is to be expected and considering the amount of performance measures that are assessed thus far, further results would only be overwhelming. For the results of the power analyses, please see the supplementary material.

Monte Carlo Error estimation is important in simulation studies because simulation studies are empirical experiments and as such the performance measures are estimations with variability. What follows is a list of Monte Carlo Error estimations for the relevant performance measures:

8 $$\begin{aligned} MCSE_{Bias}&= \sqrt{\frac{1}{n_{sim}(n_{sim} - 1)}\sum \limits _{i=1}^{n_{sim}}(\hat{\theta }_i - \theta )^2}\end{aligned}$$

9 $$\begin{aligned} MCSE_{MSE}&= \sqrt{\frac{\sum \nolimits _{i=1}^{n_{sim}} [(\hat{\theta _i} - \theta )^2 - \widehat{MSE}]^2}{n_{sim}(n_{sim}-1)}}\end{aligned}$$

10 $$\begin{aligned} MCSE_{EmpSE}&= \frac{\widehat{EmpSE}}{\sqrt{2(n_{sim}-1)}}\end{aligned}$$

11 $$\begin{aligned} MCSE_{Coverage}&= \sqrt{\frac{\widehat{Coverage}\cdot (1-\widehat{Coverage})}{n_{sim}}}\end{aligned}$$

12 $$\begin{aligned} MCSE_{Power}&= \sqrt{\frac{\widehat{Power}\cdot (1-\widehat{Power})}{n_{sim}}} \end{aligned}$$

## Results

The simulation study included seven different methods, namely Complete Case Analysis (CCA), Worst Case Scenario (WC), Random Hot Deck (RHD), Multiple Imputation by Chained Equations (MICE), and the Maximum Likelihood Missing Completely At Random (MLMCAR), Maximum Likelihood Missing At Random (MLMAR) and Weighted Least Squares Missing Completely At Random (WLSMCAR) approaches that are based on a product multinomial framework. Each method was applied to the 729,000 data sets, resulting in 5,103,000 final (pseudo-complete) data sets. In total, the entire simulation ran for about 8h 54min.

No method had significant problems which would have rendered creating a pseudo-complete dataset impossible. There were, however, two different simulated data sets that only had index test results of one type present, i.e. all positive or all negative index test results were amputated, which had to be excluded in order to guarantee sensible imputation.

In this section, Nested Loop Plots will serve as visual presentation of the results [38]. Nested Loop Plots prove to be viable for the presentation of results for many different scenarios in simulation studies. The steps of the black step functions at the top of the Nested Loop Plot indicate the scenario it refers to. If a scenario has the values 0.1, 0.2, and 0.3 for example, the lower step function refers to 0.1, the middle step function refers to 0.2 and the upper step function step refers to 0.3. The different simulation parameter are arranged one above the other at the top of the Nested Loop Plot, so that each combination of simulation parameters—and thus every scenario—can be displayed. At the bottom of the Nested Loop Plot, the different methods compared in this study are shown with one line per method. In this way, the methods can be compared for each distinct scenario and a pattern across all scenarios can be displayed.

In the case of this study, these plots help to see that the bias for sensitivity and specificity has a consistent pattern across missingness mechanism: specificity is positively biased, while sensitivity is negatively biased. This holds true for all methods except for worst case scenario. For the remainder of this section, the results for bias and coverage will be categorized by missingness mechanism. At the end of this section, the MSE and MCSE results, as well as the confidence interval lengths, will be described briefly (further information on these performance measures and on the empirical Standard Error can be found in the supplementary material).

### Missing completely at random

#### Bias

Under MCAR, sensitivity (Figure S1 in supplementary material) as well as specificity (Figure S2 in supplementary material) estimates are on average unbiased for all methods except the Worst Case method. For sensitivity estimates, there seems to be a slight increase in bias at a missingness proportion of 0.5 and a prevalence of the target condition of 0.1 whenever the sample size is 400. Especially RHD, MLMCAR and MICE show an increase in bias here. The increase can be explained by the fact that in the case of a missingness proportion of 0.5, a prevalence of the target condition of 0.1 and a total sample size of 400, the average sample size to estimate sensitivity from under MCAR is 20. The specificity estimates only have a slight increase in bias for MICE at a missingness proportion of 0.5.

#### Coverage

Coverage under MCAR is not optimal for all methods (Figure S3 and S4 in supplementary material), meaning that the coverage is consistently below 0.95, especially for higher missingness proportions. While MICE and CCA seem to perform acceptably under MCAR, the other methods perform considerably worse, with WC being the worst performer, followed by RHD and then the Product Multinomial methods.

### Missing at random

#### Bias

**Fig. 1:**
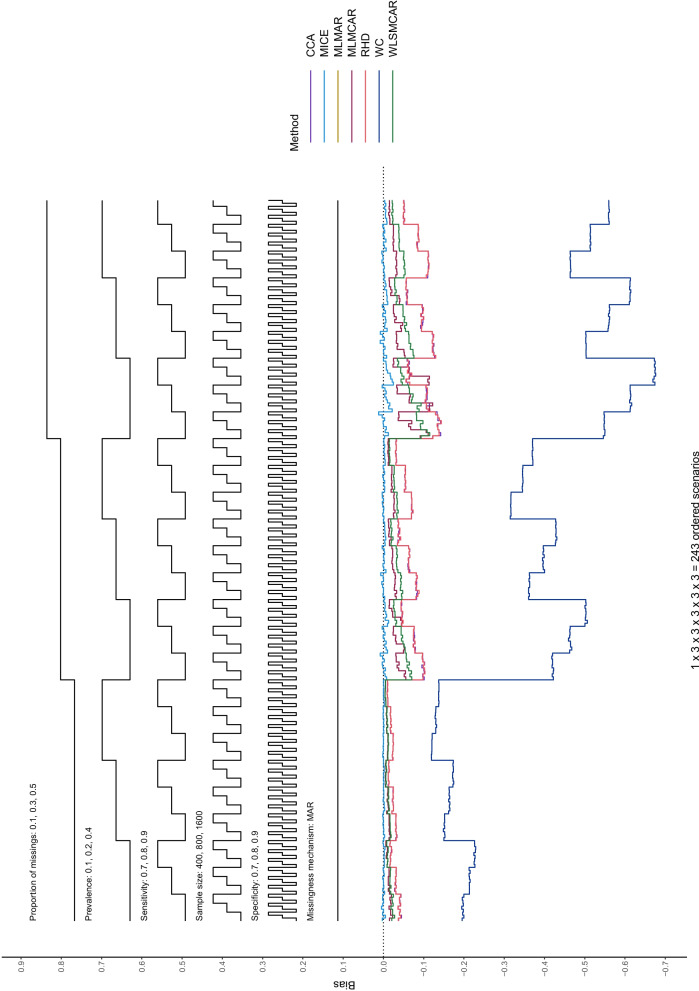
Bias of sensitivity estimates of all methods across the distinct scenarios under MAR

**Fig. 2:**
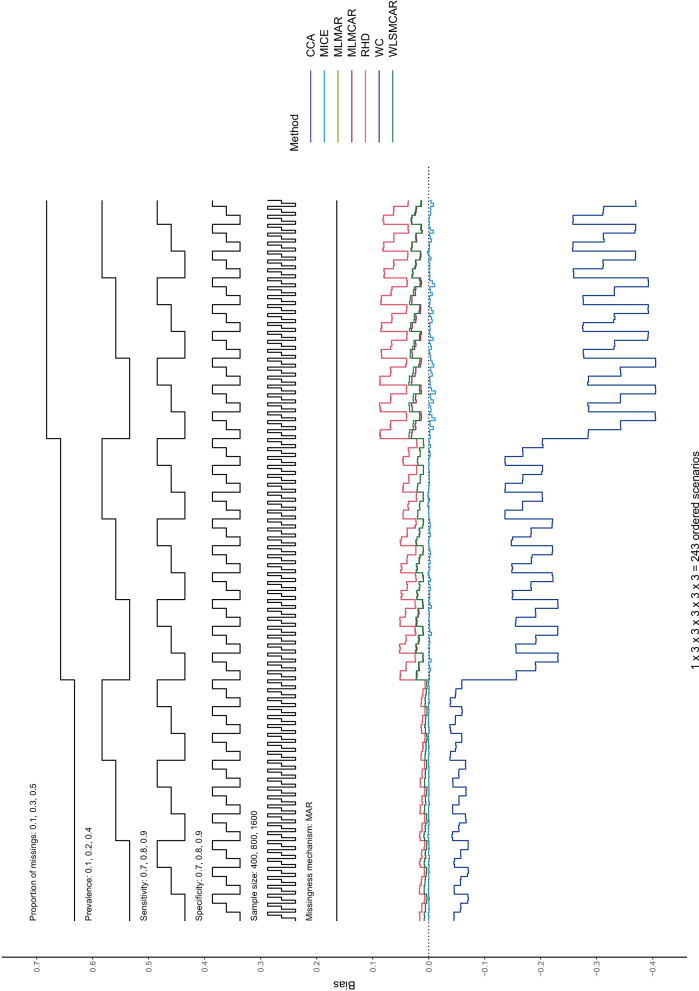
Bias of specificity estimates of all methods across the distinct scenarios under MAR

Different from MCAR, bias starts to become apparent in MAR for all methods (Figs. 1 and 2). Beside WC, RHD and CCA show the most amount of bias, MLMCAR, MLMAR, and WLSMCAR sit in the middle, and MICE only shows a negligible amount of bias. Aside from the missingness mechanism, the main driver behind the bias seems to be the missingness proportion: An increase in missingness proportion results in an increase in bias. With a missingness proportion of 0.1, all methods (except for WC) show small bias. With a missingness proportion of 0.5, MICE is the only methods that is almost unbiased, even though more bias can be seen here than for lower missingness proportions. Sample size and prevalence of the target condition do not seem to affect the bias. When comparing $m = 5$ to $m = 50$ imputations, there does not seem to be a substantial difference in terms of bias (see Figures S9 – S12 in the supplementary material).

It can also be seen that the bias pattern of specificity (Fig. 2) under MAR is virtually the same as the bias pattern of sensitivity (Fig. 1) in MAR, the biggest difference being that specificity estimates have a consistently positive and sensitivity estimates have a consistently negative bias. Further, the bias for sensitivity estimates is slightly larger. This similarity in the bias pattern seems to further suggest that sample size does not affect the bias notably, as the sample size for sensitivity estimates is smaller in this study.

#### Coverage

**Fig. 3:**
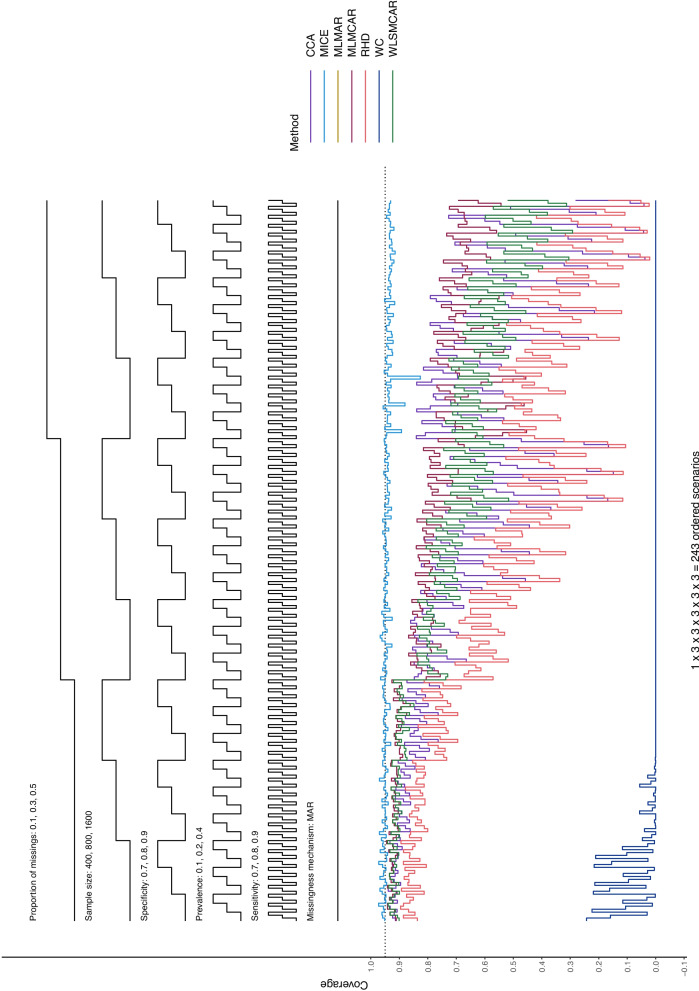
Logit coverage probability for sensitivity estimates of all methods across the distinct scenarios under MAR

**Fig. 4:**
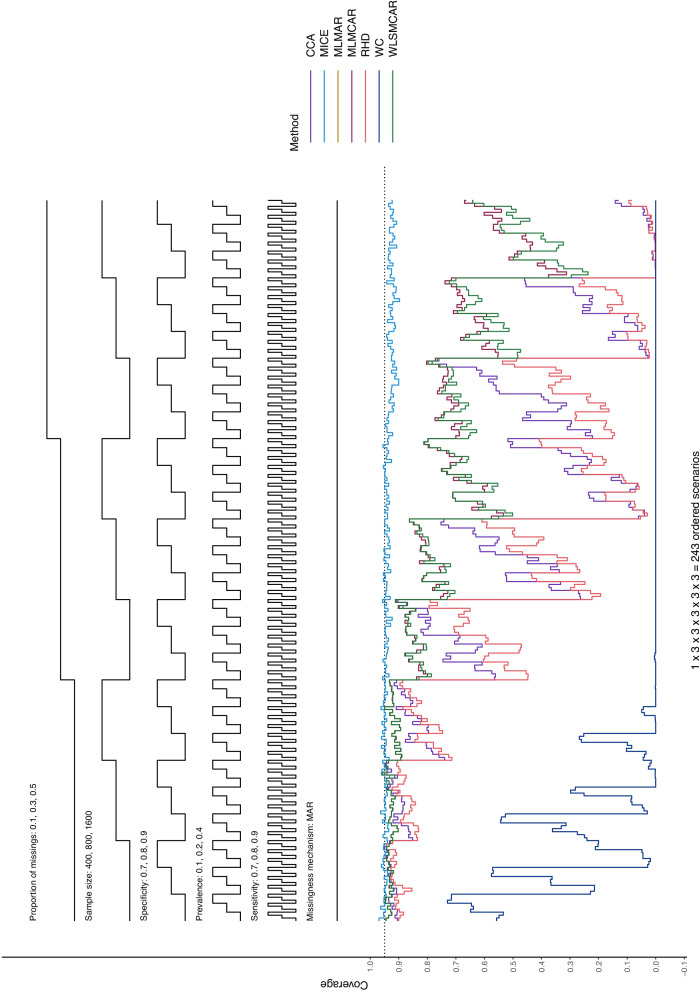
Logit coverage probability for specificity estimates of all methods across the distinct scenarios under MAR

Under MAR (Figs. 3 and 4), MICE is the only method that seems to perform well concerning coverage, with some small outliers for sensitivity estimates when the missingness proportion is high and the sample size is small. All other methods show lacking coverage, worsening with an increasing proportion of missings, and for lower specificity for specificity estimates. It is important to note, however, that MICE’s coverage is consistently slightly below 0.95 for a missingness proportion of 0.5 (using $m = 50$ imputations leads similar but slightly better coverage, especially for high missingness, as can be seen in Figures S17 – 20). While sensitivity and specificity show similar patterns, sensitivity seems to have better coverage overall. For all other methods, the coverage is very volatile, and it can only be said—with some certainty—that an increase in the proportion of missingness leads to lower coverage.

### Missing not at random

#### Bias

Compared to the bias under MCAR and MAR, the bias for MNAR is more severe (Figure S5 and S6 in supplementary material). While the bias with a missingness proportion of 0.1 is fairly small, it gets more extreme with increasing missingness. Overall, MICE seems to have the smallest bias, but its bias is very close to the bias of the other methods, different from MCAR and MAR where MICE was unbiased compared to the other methods. Similarly, there seems to be no difference in the performance of MICE when using $m = 50$ compared to $m = 5$ (see Figures S13 – S16 supplementary material). When it comes to the other methods, RHD has the largest bias, with all the other methods being in the middle, except for the worst case method.

#### Coverage

For MNAR (Figure S7 and S8 in supplementary material), coverage for all methods is far from ideal. Even at a missingness proportion of 0.1, all methods show a bad performance, which only worsens with an increasing missingness proportion. Again, sensitivity and specificity show similar patterns, but sensitivity has better coverage overall. Choosing $m = 5$ or $m = 50$ for MICE does not make a difference (Figures S21 – S24 in supplementary material).

### Confidence interval lengths

Confidence interval lengths tend to be shorter for specificity estimates than for sensitivity estimates (Table 4) because sensitivity is estimated on a smaller sample size than specificity for prevalences of the target condition below 0.5. The confidence interval lengths of CCA and MICE are clearly longer than the lengths of most other methods. Only WC seems to have long average confidence interval lengths, too. As for the other methods, their confidence interval lengths are similarly short.

**Table 4:** Logit confidence interval lengths for sensitivity and specificity estimates

	Sensitivity			Specificity		
Imputation Method	Min	Median	Max	Min	Median	Max
CCA	0.0398	0.1683	0.8823	0.0085	0.0643	0.1890
WC	0.0421	0.1450	0.3003	0.0318	0.0729	0.1258
RHD	0.0281	0.1293	0.3003	0.0048	0.0543	0.1258
MICE	0.0367	0.1519	0.9882	0.0102	0.0700	0.5099
MLMCAR	0.0335	0.1279	1	0.0051	0.0563	1
MLMAR	0.0335	0.1279	1	0.0051	0.0563	1
WLSMCAR	0.0335	0.1287	1	0.0051	0.0563	1

### Mean squared error

In general, the MSE seems to behave very similarly to the bias, meaning that for scenarios where bias is low, MSE is also low, and for scenarios where bias is high, MSE is also high. Similarly, aside from the missingness mechanism, the proportion of missingness seems to be the main driver for an increase in MSE. Please see the supplementary material for more details.

### Monte Carlo standard error

MCSE values are very low, as can illustratively be seen in Table 5 for bias and coverage, indicating precise estimation. For all MCSE tables, please see the supplementary material.

**Table 5:** Monte Carlo error for bias and coverage

	Bias			Coverage		
Measure of Accuracy	Min	Median	Max	Min	Median	Max
Sensitivity	0.0004	0.0013	0.0085	0	0.0091	0.0158
Specificity	0.0002	0.0006	0.0017	0	0.0080	0.0158

## Discussion

The results highlight that MICE outperforms all methods relatively consistently, especially when it comes to coverage and bias. For bias under MCAR, most methods perform reasonably well. Only when sample sizes are very small (prevalence of the target condition of 0.1 and missingness proportion of 0.5 for sensitivity estimates with a total sample size of 400, meaning an effective sample size of 20), RHD, MLMCAR and MICE show some slight bias. This seems to be too small of a sample size for these methods to function perfectly. Most methods start to show bias under MAR. However, MICE is only either unbiased for low missingness proportions or not relevantly biased for higher missing proportions. Other methods show an increase in bias for an increase in missingness proportion under MAR. In MNAR, all methods are very biased, especially for high missingness proportions.

For coverage, only MICE performs well, even in MCAR where most methods are unbiased. An explanation for the coverage probability being too low despite unbiasedness for RHD, MLMCAR, MLMAR, and WLSMCAR is that these methods underestimate the variance, which is indicated by the corresponding short confidence interval lengths as can be illustratively seen in Table 4 (for all confidence interval lengths, please see the supplementary material). Single imputation approaches are known to underestimate variance [19, 39]. However, Andridge and Little [19] recommend using other types of confidence intervals, such as bootstrap-based ones and RHD combined with MI, which results in larger confidence interval lengths and achieve a better coverage. In contrast, CCA has a lower sample size as it only includes complete cases, resulting in variance that is not underestimated. Additionally, Multiple Imputation as a procedure, which MICE is based on, was specifically designed not to underestimate variance.

Having MICE as the clear outperformer in this simulation study is helpful because MICE already is a popular imputation method and readily available in common statistical software such as R, SAS, and SPSS. There should be caution, however, before MICE can be declared as the best possible method with certainty for multiple reasons. MICE, as a multiple imputation approach, can be considered to have an advantage over single imputation methods. Nevertheless, the product multinomial methods (MLMCAR, MLMAR, WLSMCAR) show somewhat promising results, and it is not clear whether these methods could be improved upon with multiple imputation and corresponding pooling guidelines. Secondly, MICE, alongside the other (improved) methods, needs to undergo further stress tests to determine whether it performs well in scenarios not considered here. As our supplementary results show that using a higher number of imputation results in higher coverage probability under MAR, we recommend to adhere to common recommendations regarding the number of imputations for MICE. For instance, White et al. [23] strengthen the recommendation to use as many imputations as the proportion of missing values.

CCA and WC did not offer any information that was not clear before conducting the simulation study, but rather illustrated the need for more sophisticated missing data handling methods, especially in light of the fact that most researchers in diagnostic studies only apply complete case analyses. Additionally, it is not clear how useful WC is as a sensitivity measure in a simulation study, since the performance measures for WC were far off all the other methods.

Although RHD did not perform perfectly, it did perform decently, considering that donor selection was completely random in the two disease groups. If the donor selection were instead informed by more sophisticated distance measures, for example maximum deviation or Mahalanobis distance [19], RHD could show more promising performance. Additionally, as mentioned before, RHD is a method that has seen a lot of application in survey studies, which often deal with nominal or ordinal variables. In a scenario where an index test that is not dichotomous, and instead ordinal, has missing data, RHD (with a sophisticated distance measure) might therefore be a worthwhile consideration and needs to be examined accordingly.

The product multinomial framework based methods (MLMCAR, MLMAR, and WLSMCAR) performed reasonably well but unfortunately did not perform as well as expected considering their extensive theoretical foundation [34]. Poleto et al. [34] showed these methods’ practical usability in a study that considered multiple index tests where missings occurred in each index test. It could therefore be presumed that these methods perform better in situations where either multiple index tests or, equivalently, multiple dichotomous variables in the data set are missing. Also, since the index tests in the example study of Poleto et al. [34] related to the same underlying disease, it can be presumed that the test results were highly correlated. This could mean that a possible ideal circumstance for these methods is having highly correlated index tests or covariates to be imputed.

The product multinomial framework offers two more methods that were not included in this simulation study, namely a weighted least squares approach able to handle MNAR data [40] and a Bayesian extension of the framework [41]. Due to technical difficulties, these methods could not be implemented in this study.

The difficulty of implementation of (new) missing data methods is an important limiting factor [5]. Of the methods compared in this study, CCA and WC were the easiest to implement, which might be apparent from the simplicity of their procedures as a whole. RHD was fairly easy to implement, too, given that an appropriate R package was used, as there are a number of R packages that include RHD as a procedure with differing ease of implementation. Implementing MICE is challenging since a considerable amount of statistical understanding of MICE is needed. Nonetheless, extensive literature about multiple imputation and its application is available [23, 42]. The product multinomial methods (MLMCAR, MLMAR, WLSMCAR) are the hardest to implement. Understanding the steps that are even needed to prepare the imputation and the interpretation of the result presentations the methods provide require considerable time and effort, which might repel potential users.

The missingness mechanism seems to be one of the main concerns when choosing an appropriate imputation method as it has a large influence on the quality of imputation. While it might be fairly straightforward to simulate a certain missingness mechanism in a simulation study, determining whether data is MCAR, MAR, or MNAR is not trivial in practice. However, domain knowledge of researchers can help to in making assumptions about possible missingness mechanisms, and, therefore, a good collaboration between clinicians and statisticians is essential. Furthermore some statistical methods may assist in determining the missingness mechanism, such as Little’s MCAR test [43, 44] for multivariate normally distributed data. Statistical tests for determining MCAR in binary data—as in our case here—are not that common. Most suitable for binary data may be a MCAR test by Fuchs [45] or by Berrett and Samworth [46] with the latter also being available as R package. Nonetheless, we encourage the readers to look for the most suitable MCAR test for their data (an overview of existent tests is provided by Aleksić [47]) and not to rely solely on statistical test but also on their theoretical assumptions. Additionally, Lee et al. [48] propose that data examination is another practical tool. Further, van Buuren et al. [31] claim that the inclusion of more rather than less predictor variables in the imputation model (for MICE, at least) ensures that the MAR assumption is more likely to be fulfilled. Still, van Buuren et al. [31] emphasize that not every available variable in a data set should be made part of the imputation model, as this would lead to giant models with hundreds of variables. Instead, it is proposed, as a rule of thumb, that the best 15 variables should be chosen. Variable selection strategies for the imputation model in MICE are outlined in van Buuren et al. [31] and Graham [27].

The results of this simulation study are partly in line with a recent simulation study on handling missing values in a continuous index test [6]. In both studies, MICE showed biased results if the effective sample size is small (small sample size, low prevalence of the target condition and high proportion of missing values). Nonetheless, MICE still outperformed the other methods in these scenarios in this simulation study, whereas it was superseded by the CCA and an augmented inverse probability weighting approach when estimating the AUC [6]. Therefore, this simulation study would cautiously recommend using MICE when missing values occur in a binary index test and sensitivity and specificity are estimated. In contrast, CCA and the augmented inverse probability weighting approach should be favored for estimating the AUC if the sample size is small and MICE (and joint modelling MI) if the sample size is large [6].

Some limitations have to be noted. Despite comprising a large set of scenarios, this study lacks the examination of varying correlation structures between the index test and covariates and of including continuous covariates. Further research is needed to investigate these additional scenarios. As one reviewer pointed out, the data generation follows a probit-type construction whereas a logistic regression is used as imputation method in MICE. Despite probit and logit links being similar, this is kind of a mismatch between data generation and analysis and has to be kept in mind when interpreting the results. Unfortunately, there is no probit based imputation available for MICE in R. Quartagno and Carpenter [49] compared MICE with joint modelling MI where the former uses a logistic regression imputation (in congruence with their data generation) model and the latter implicit probit-link functions. Nonetheless, both MI approaches performed quite equally well. Therefore, we assume that this misspecification between our data generation and imputation model has only a negligible effect on the results. As MICE clearly outperforms the other methods and behaves quite well across the scenarios, we would assume that this misspecification does not have a substantial impact on our conclusions. Furthermore, the focus was on missing values in the index test only and, thus, the results cannot be transferred to cases where missing values exist in both the index test and the reference standard. Stahlmann et al. [5] provide an overview of methods that can be applied to handle missing values in both the index test and the reference standard.

Despite these limitations, this simulation study provides the first systematic comparison of methods to handle missing values in a binary index test. It includes a broad set of scenarios and methods, the latter of which were selected on the basis of a recent scoping review. Moreover, the MCSE are on average 0.002 for the bias in sensitivity and <0.001 for specificity. This indicates precise bias estimates.

## Conclusion

This simulation study compared different methods in estimating sensitivity and specificity for a binary index test with missing values. While most methods perform well for MCAR, MICE still outperformed the other methods across all scenarios, particularly regarding bias and coverage probability. Nonetheless, some caution is necessary when using MICE for a small sample with low prevalence of the target condition and high proportion of missing values.

Sensitivity and specificity estimates have clear bias patterns in this simulation study, with sensitivity showing negative bias and specificity showing positive bias. As of now, it is not clear why these bias patterns exist and whether they are reproducible in further simulation studies.

For future research, the following additional scenarios could be considered: Different correlation structures for the covariates (low correlation, high correlation, differing correlation), missing values in not only the index test but also in covariates and reference test, continuous covariates (where applicable), imperfect reference tests, and exclusion of the reference test in the imputation model. Lastly, it is necessary to put the findings into practice and assess the methods’ performance in example studies.

## Additional file

**Additional file 1.** Supplementary Material 1. The file ‘Supplements: Comparison of methods to handle missing values in a binary index test in a diagnostic accuracy study – a simulation study’ contains additional figures, tables, and results for this study.

## Data Availability

The simulated data and R programs are available at our GitHub repository (https://github.com/MisEstiDiag/missing-data-sensitivity-specificity-estimates, latest version: Juljugin, D., Stahlmann, K., Zapf, A.: Code and Data 2.0 (v2.0). https://doi.org/10.5281/zenodo.19087135)
