# Supplementary material for: Comparison of methods to handle missing values in a binary index test in a diagnostic accuracy study – a simulation study
Source: BMC Med Res Methodol. 2026 Apr 30;26:99. doi: 10.1186/s12874-026-02865-6 (PMC13130772; doi:10.1186/s12874-026-02865-6)
Supplement: Supplementary file 1 — Additional file 1. Supplementary Material 1. The file ‘Supplements: Comparison of methods to handle missing values in a binary index test in a diagnostic accuracy study – a simulation study’ contains additional figures, tables, and results for this study. [file 12874_2026_2865_MOESM1_ESM.pdf]

Supplementary Material for "Comparison of methods to handle missing values in a binary index test in a diagnostic accuracy study – a simulation study"

Dennis Juljugin[1], Katharina Stahlmann[1*†], Antonia Zapf[1†]

[1*]Institute for Medical Biometry and Epidemiology, University Medical Center Hamburg-Eppendorf, Hamburg, Germany.

*Corresponding author(s). E-mail(s): k.stahlmann@uke.de;
†Shared senior authorship.

1 Nested Loop Plots for Bias and Coverage

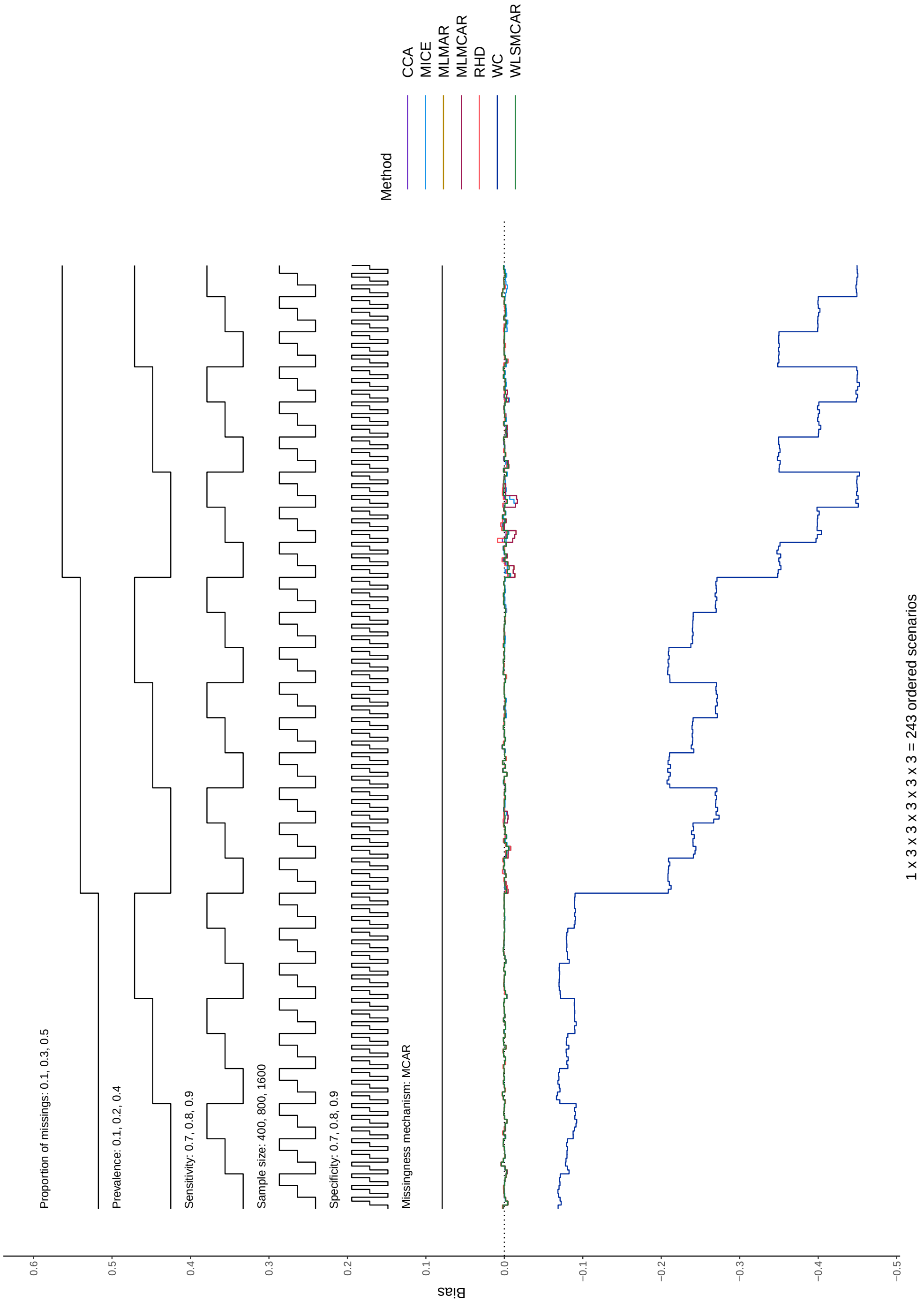

Fig. S1 Bias of sensitivity estimates of all methods across the distinct scenarios under MCAR

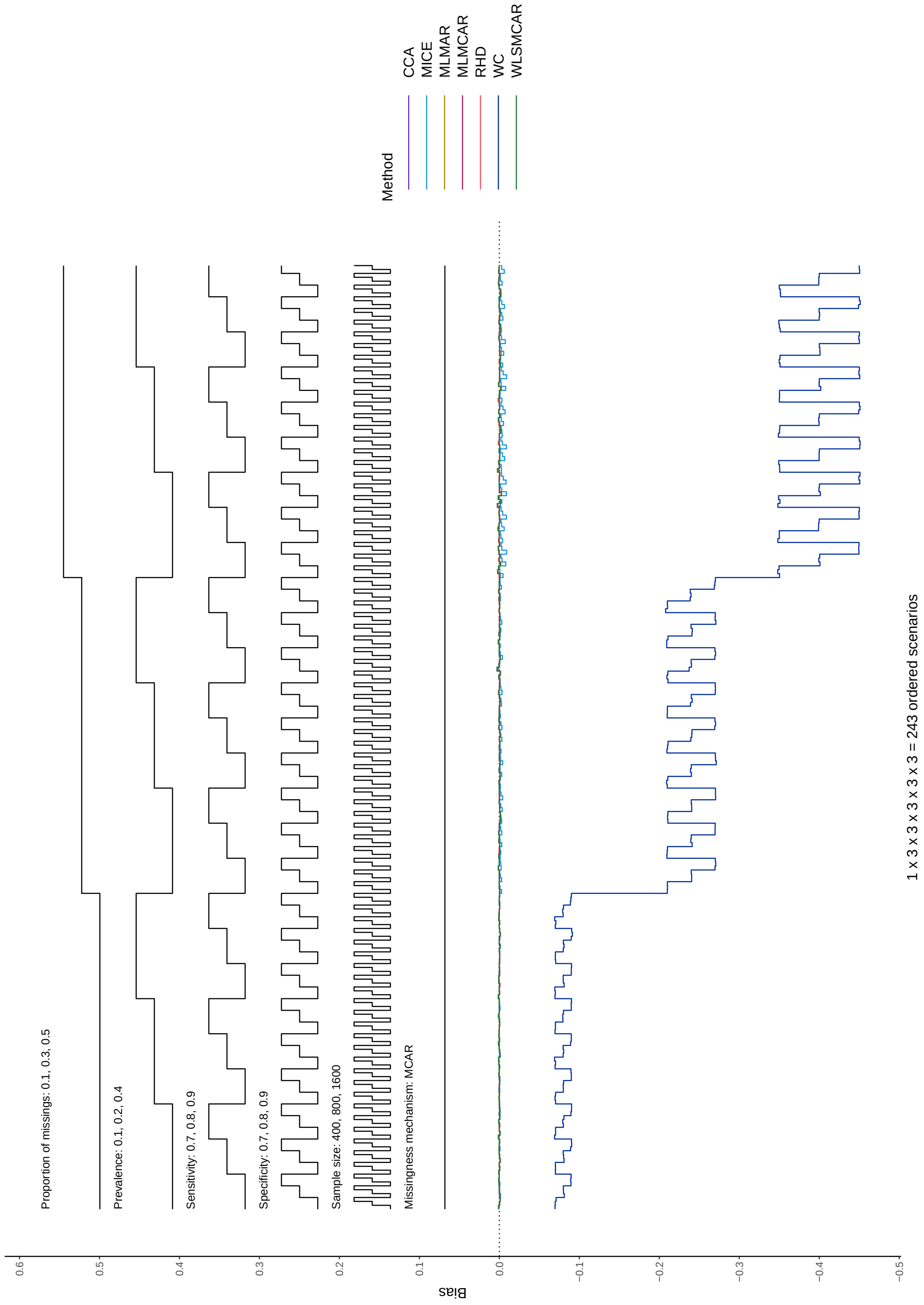

Fig. S2 Bias of specificity estimates of all methods across the distinct scenarios under MCAR

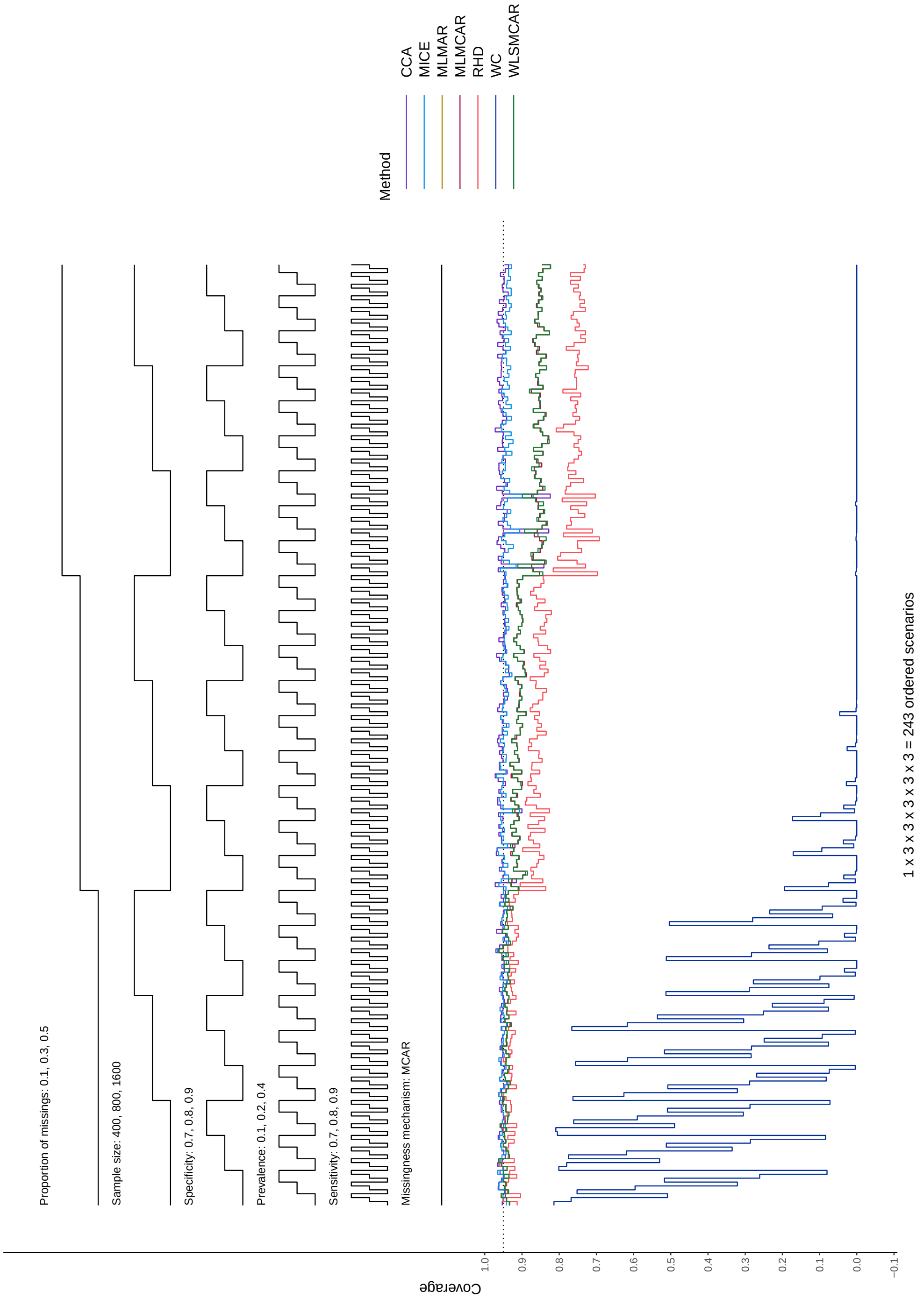

Fig. S3 Logit coverage probability for sensitivity estimates of all methods across the distinct scenarios under MCAR

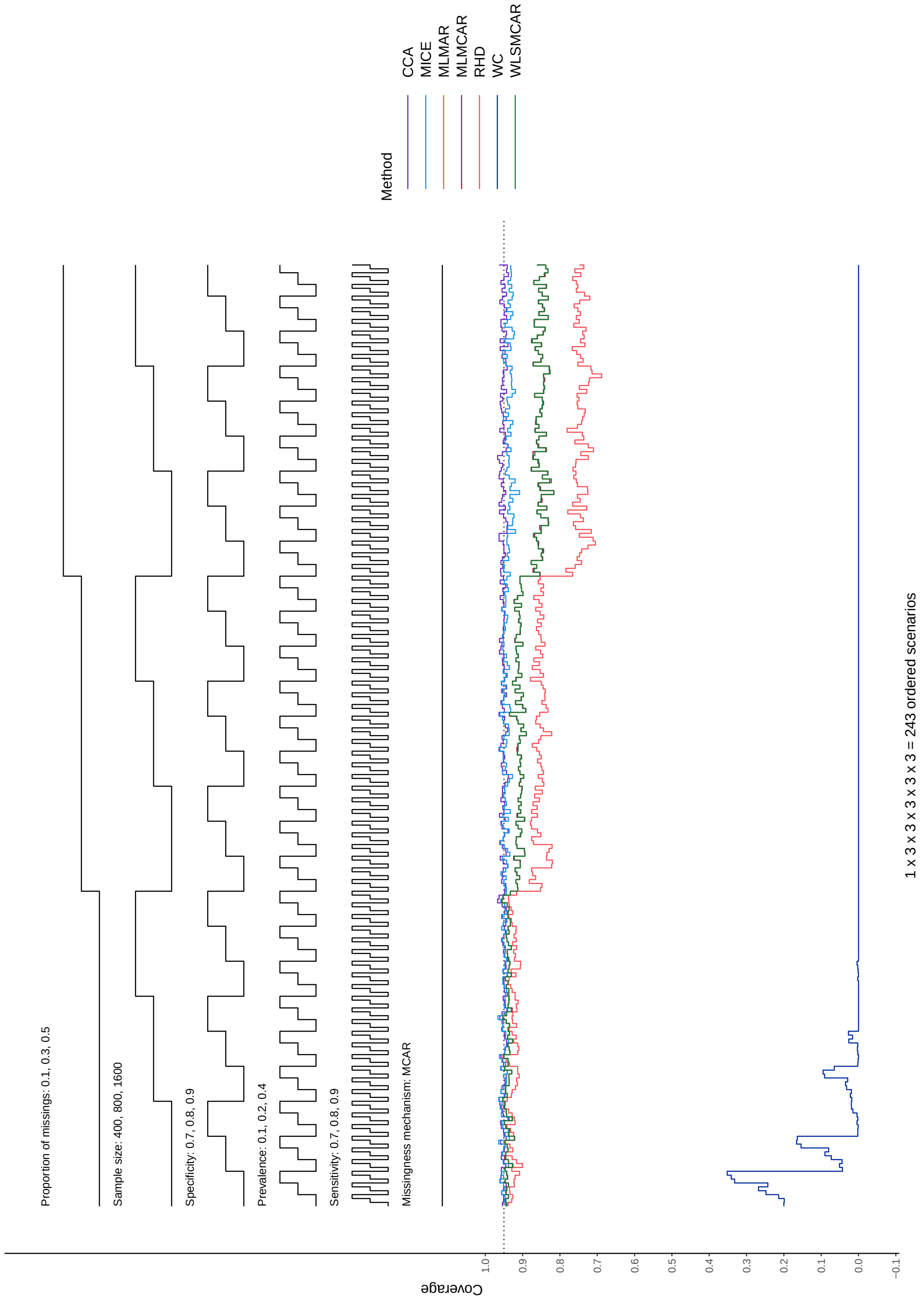

Fig. S4 Logit coverage probability for specificity estimates of all methods across the distinct scenarios under MCAR

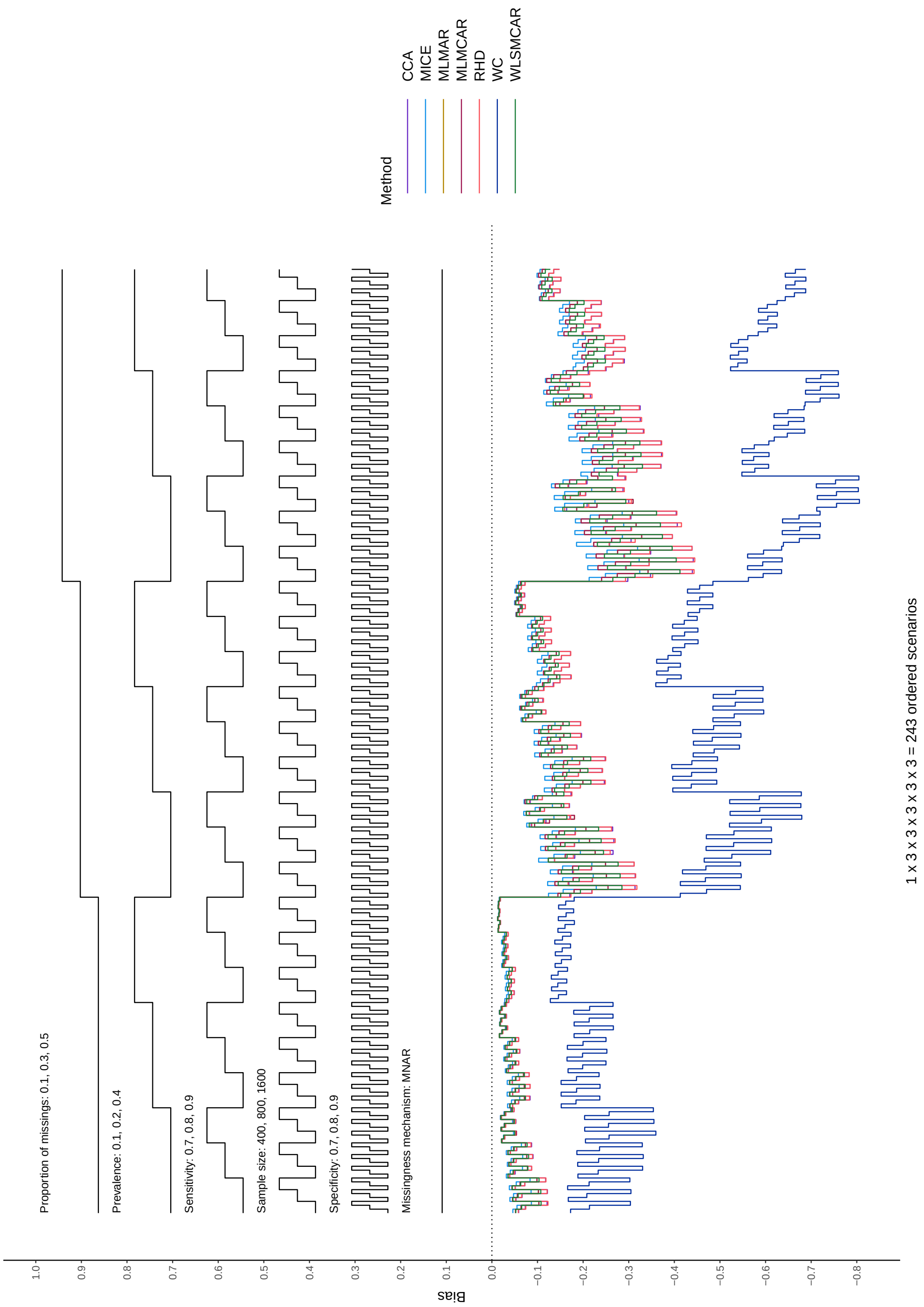

Fig. S5 Bias of sensitivity estimates of all methods across the distinct scenarios under MNAR

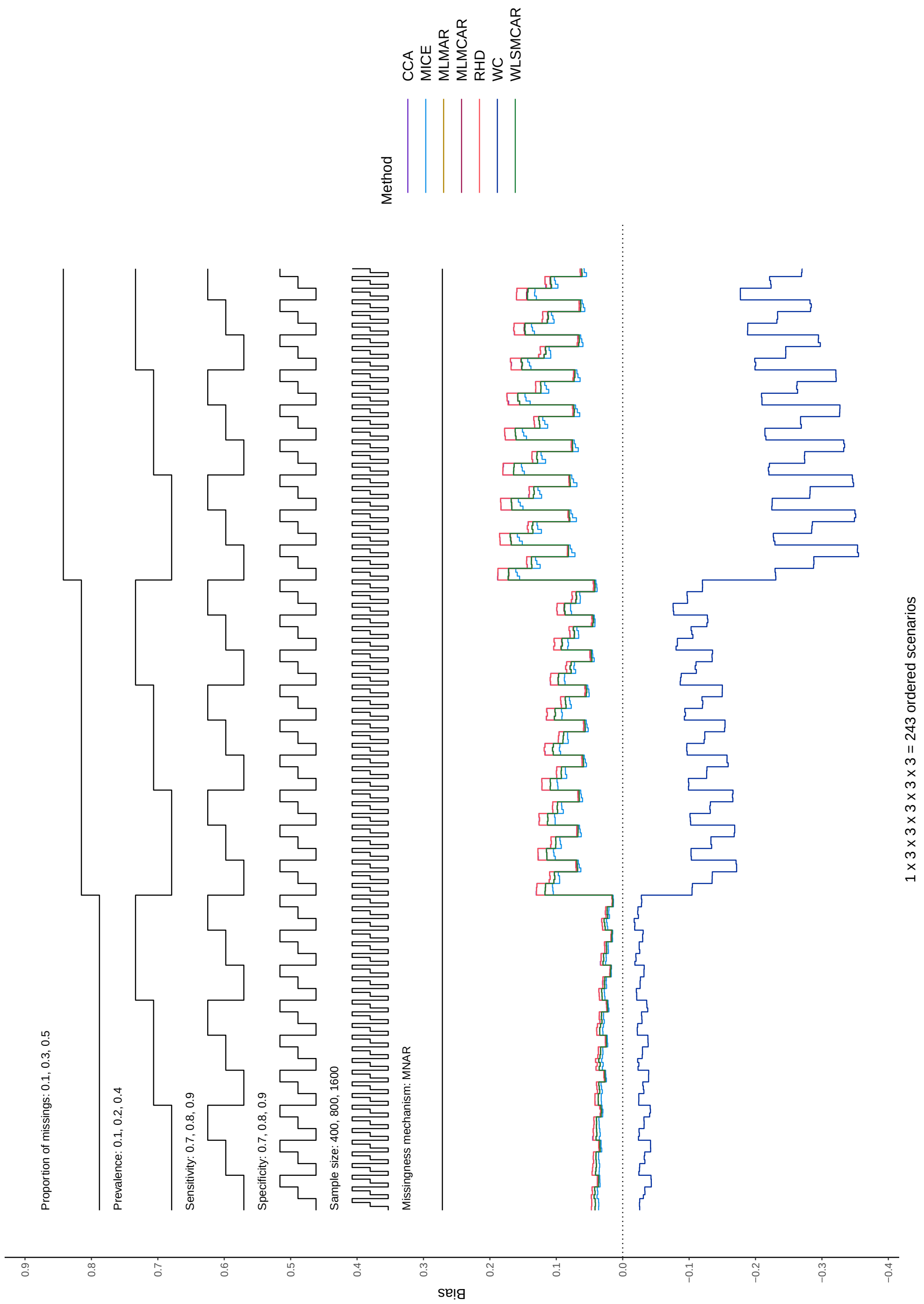

Fig. S6 Bias of specificity estimates of all methods across the distinct scenarios under MNAR

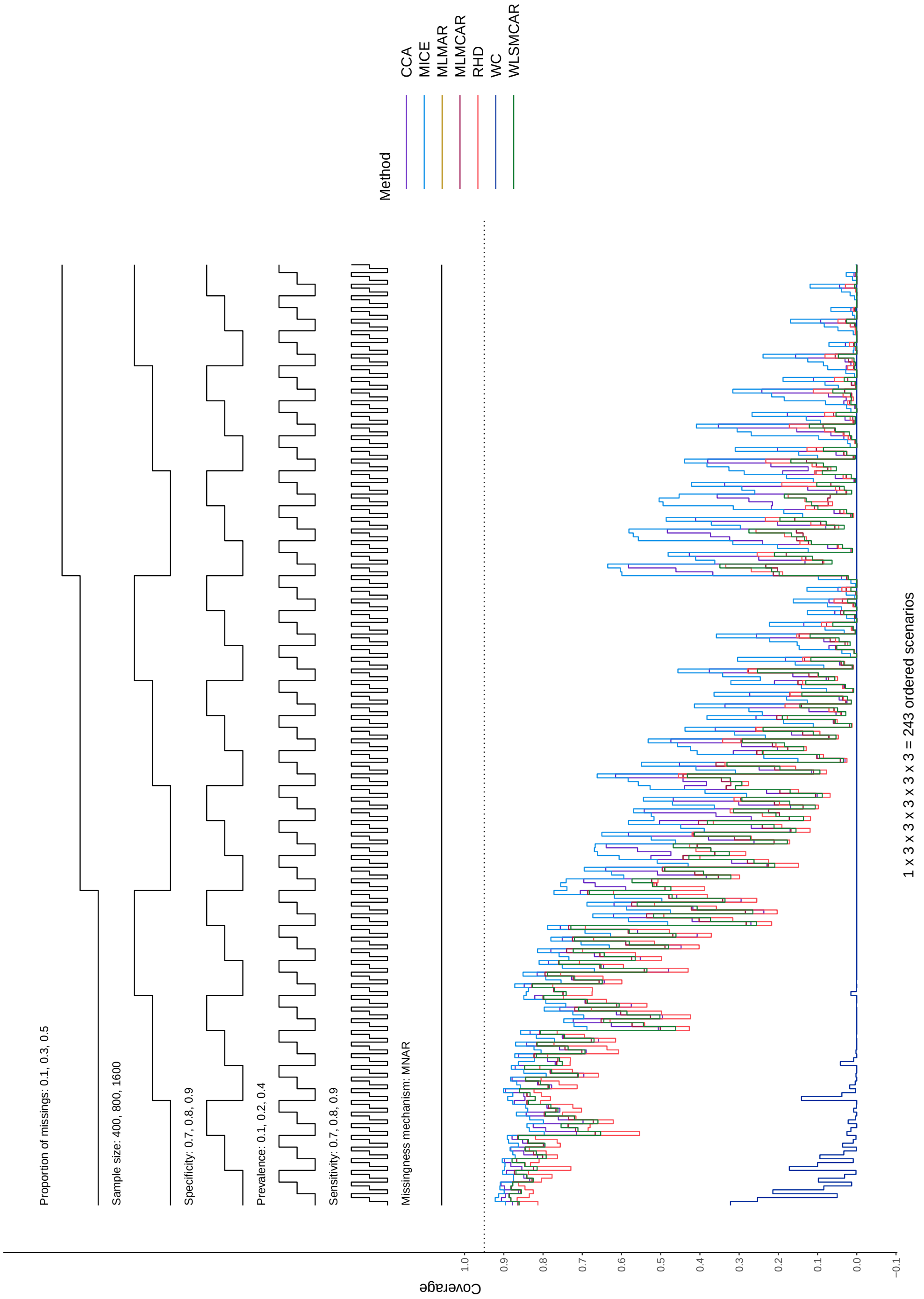

Fig. S7 Logit coverage probability for sensitivity estimates of all methods across the distinct scenarios under MNAR

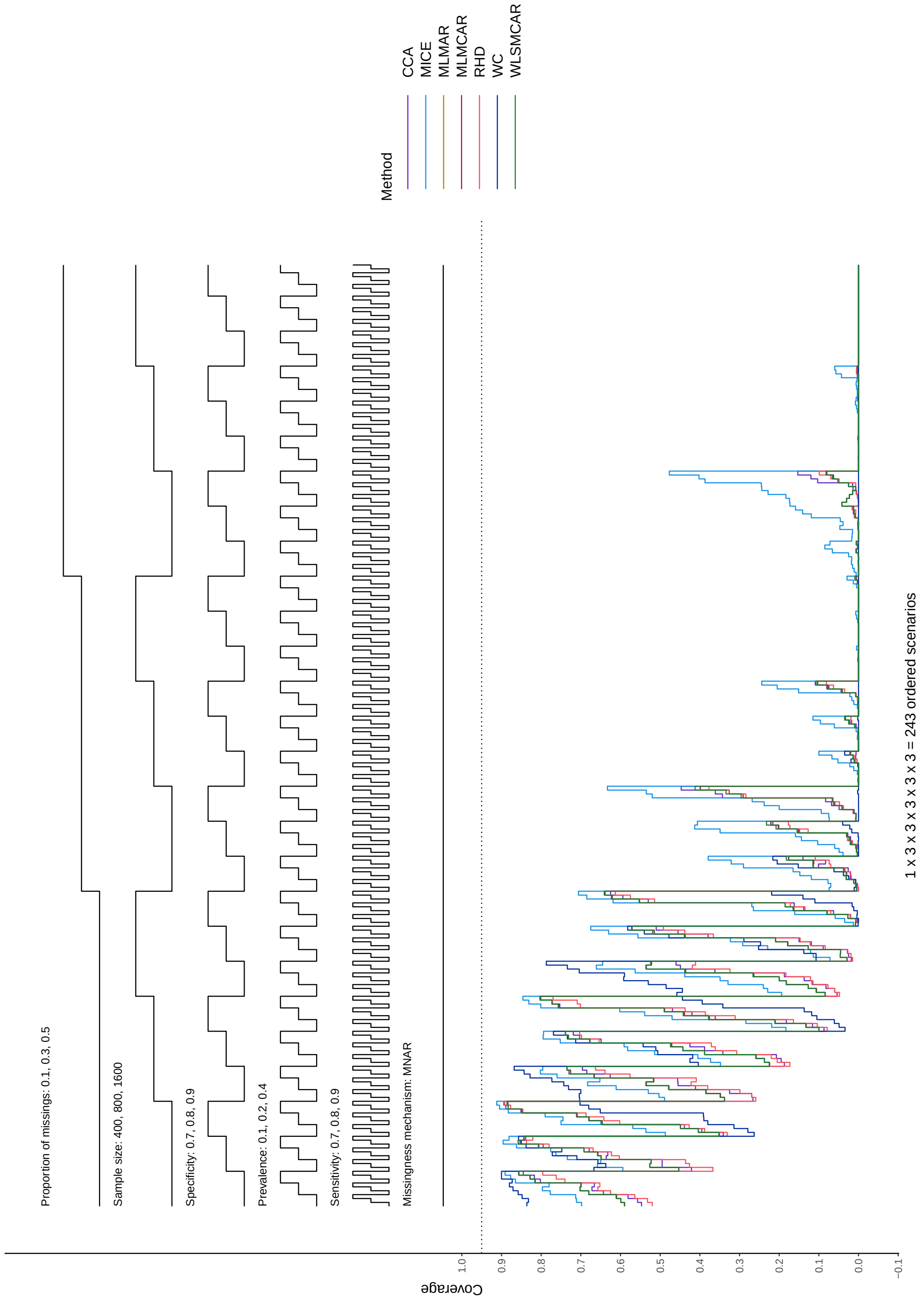

Fig. S8 Logit coverage probability for specificity estimates of all methods across the distinct scenarios under MNAR

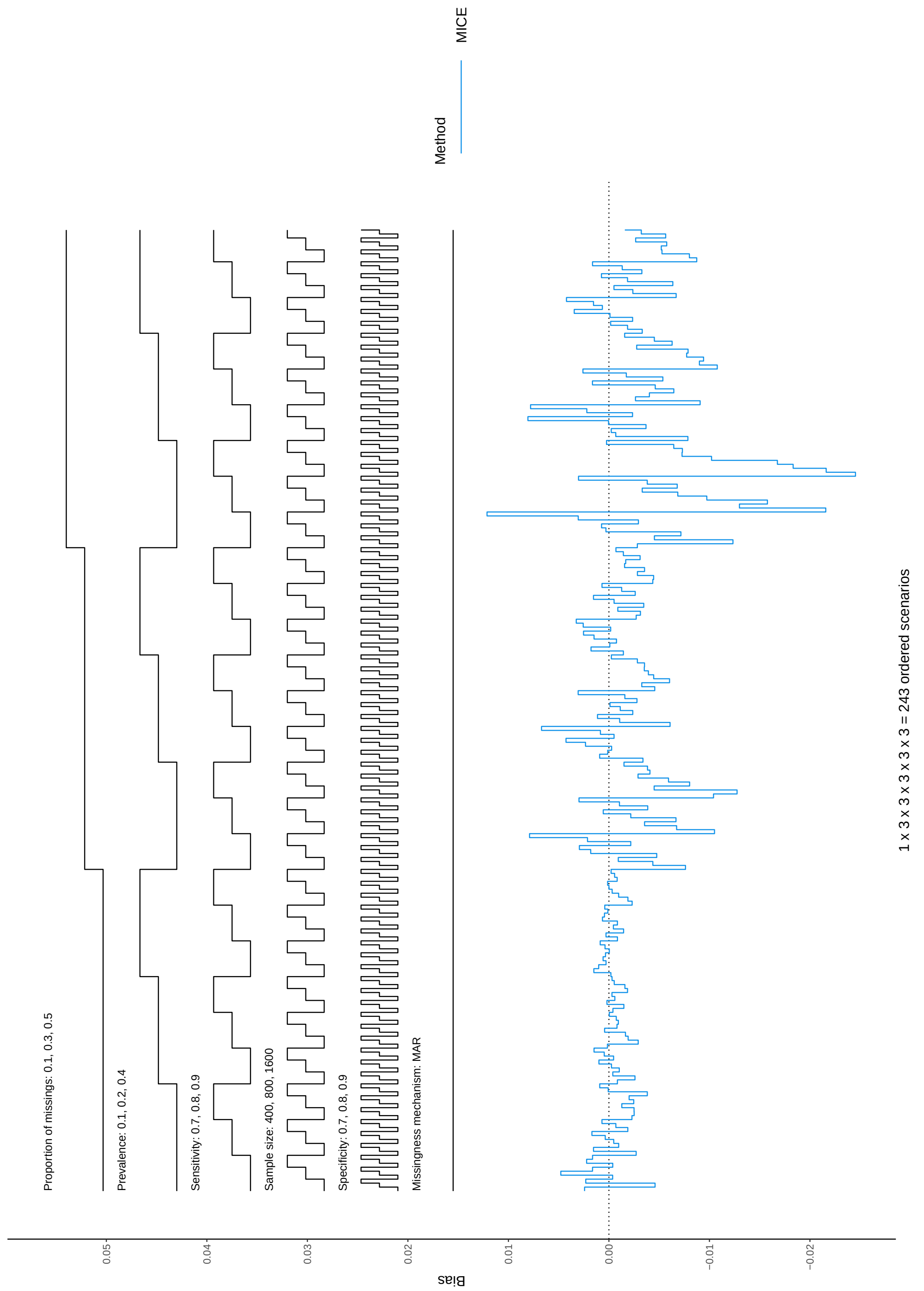

Fig. S9 Bias of sensitivity estimates of MICE with m = 5 across the distinct scenarios under MAR

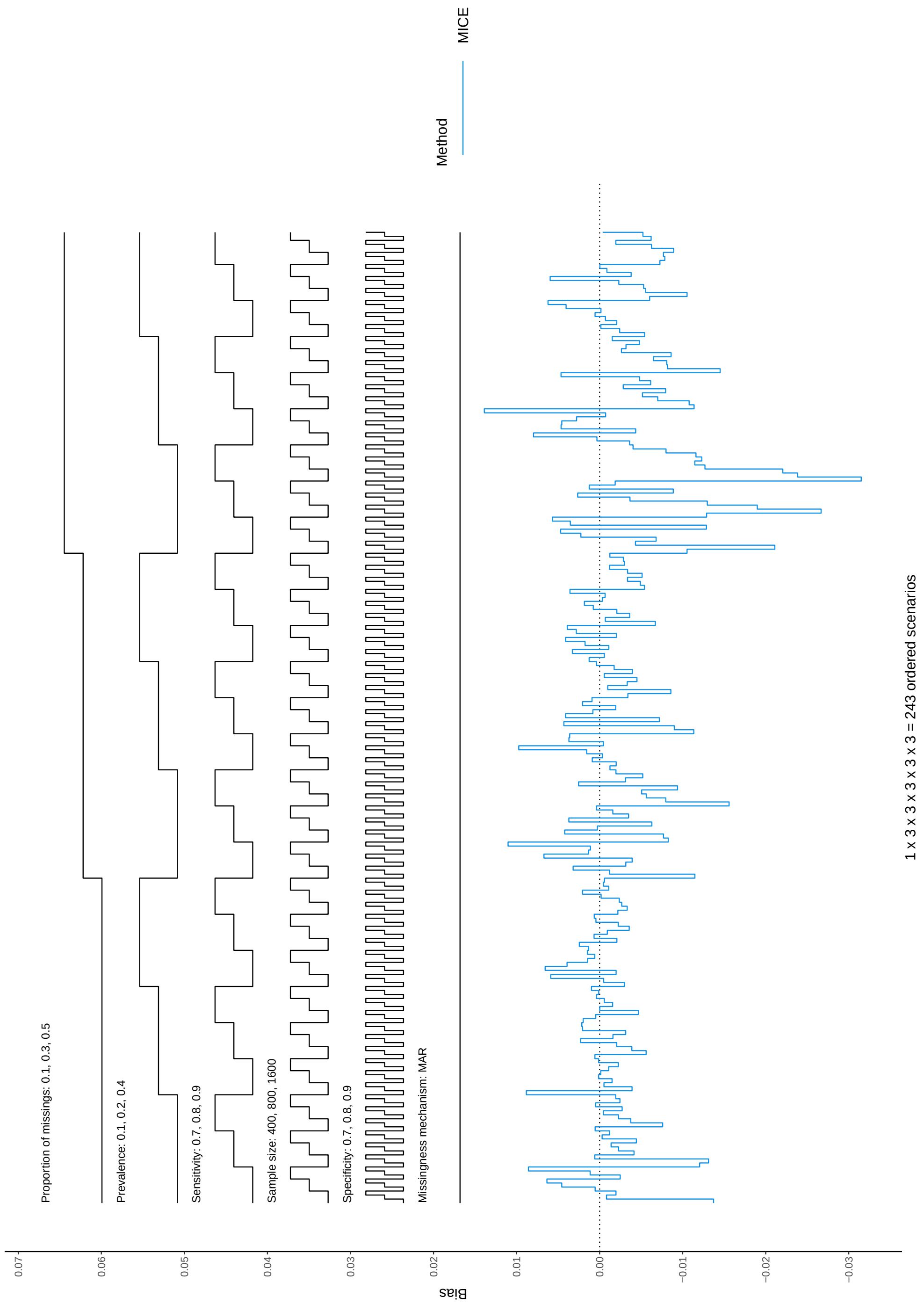

Fig. S10 Bias of sensitivity estimates of MICE with m = 50 across the distinct scenarios under MAR

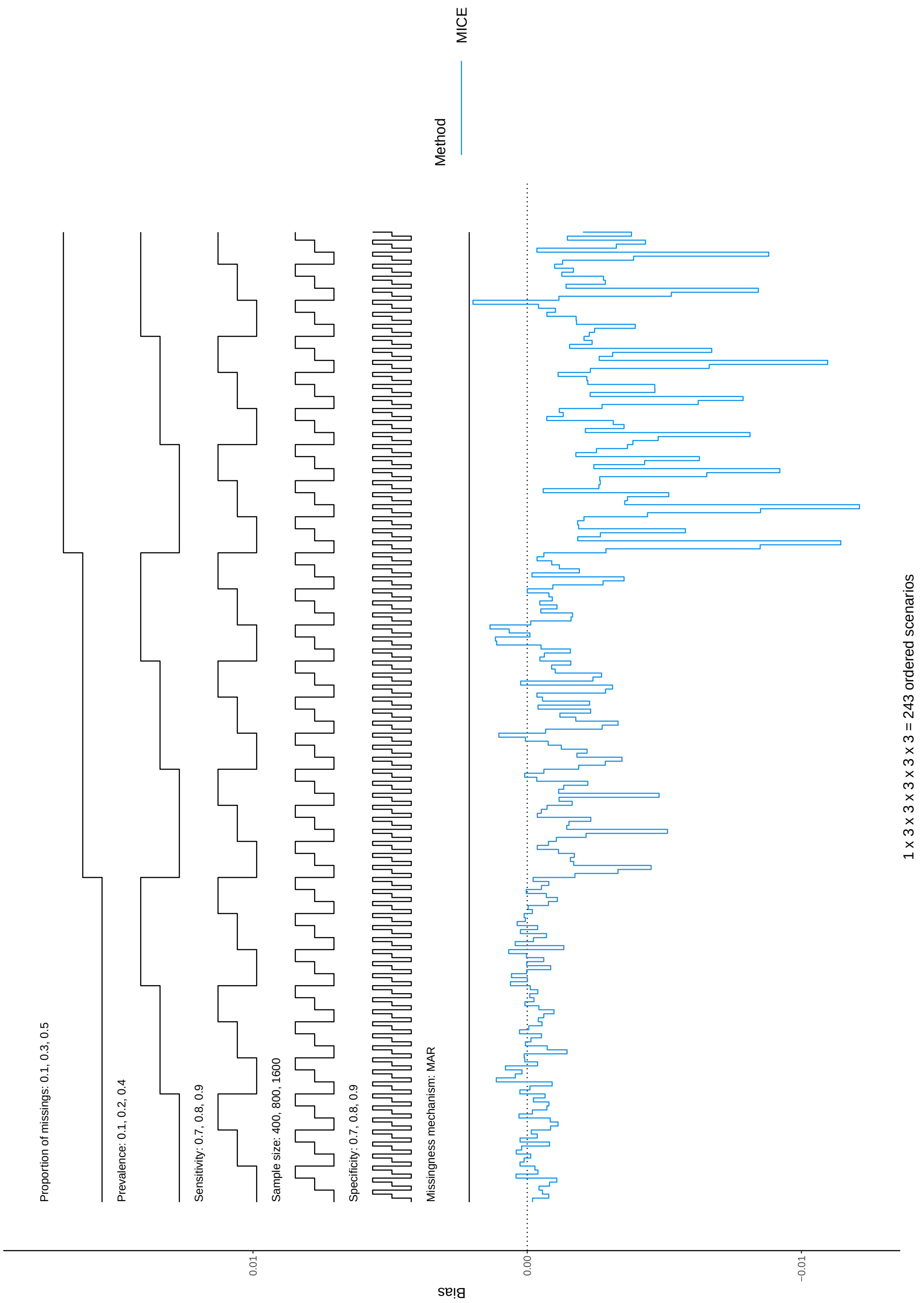

Fig. S11 Bias of specificity estimates of MICE with m = 5 across the distinct scenarios under MAR

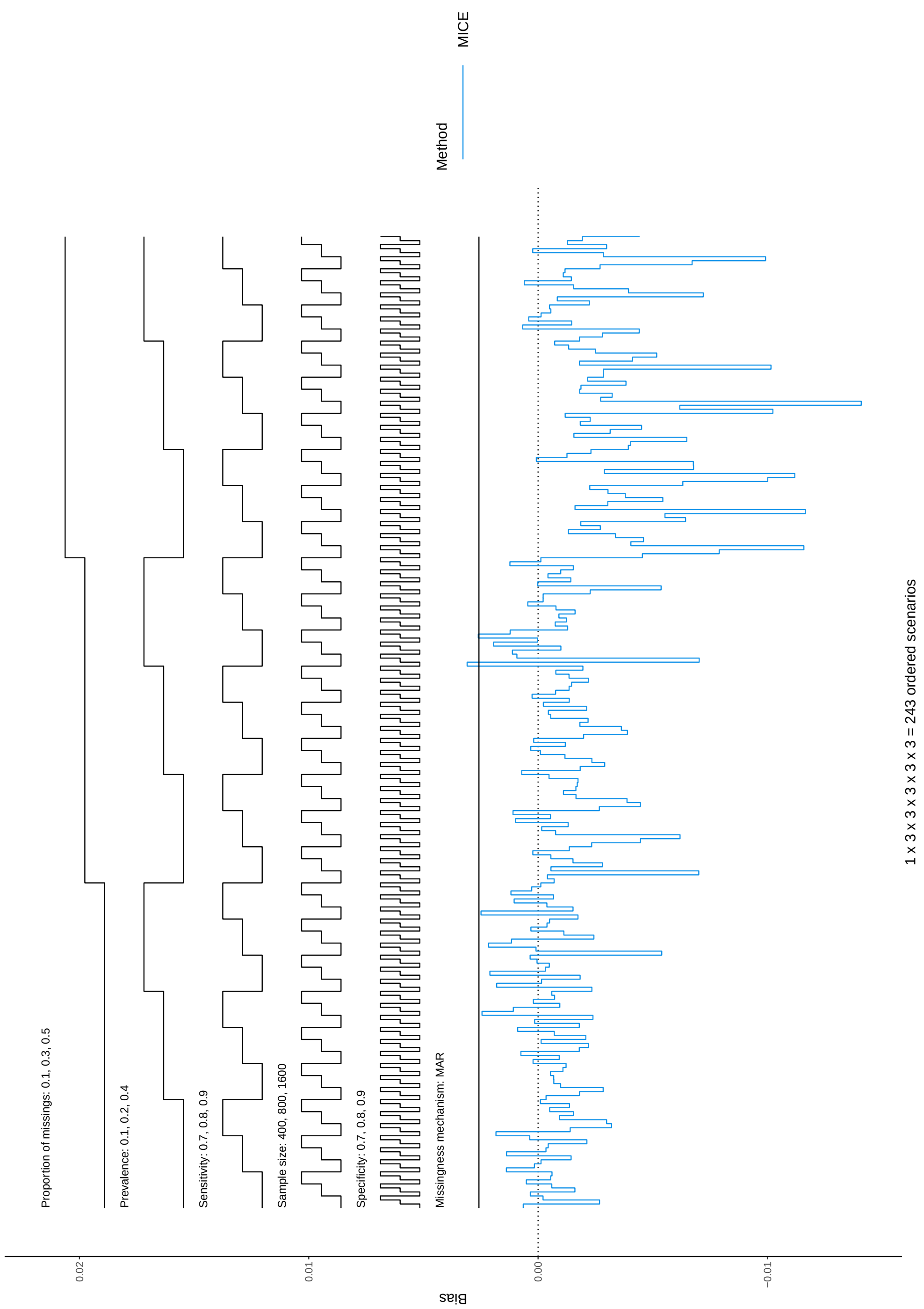

Fig. S12 Bias of specificity estimates of MICE with m = 50 across the distinct scenarios under MAR

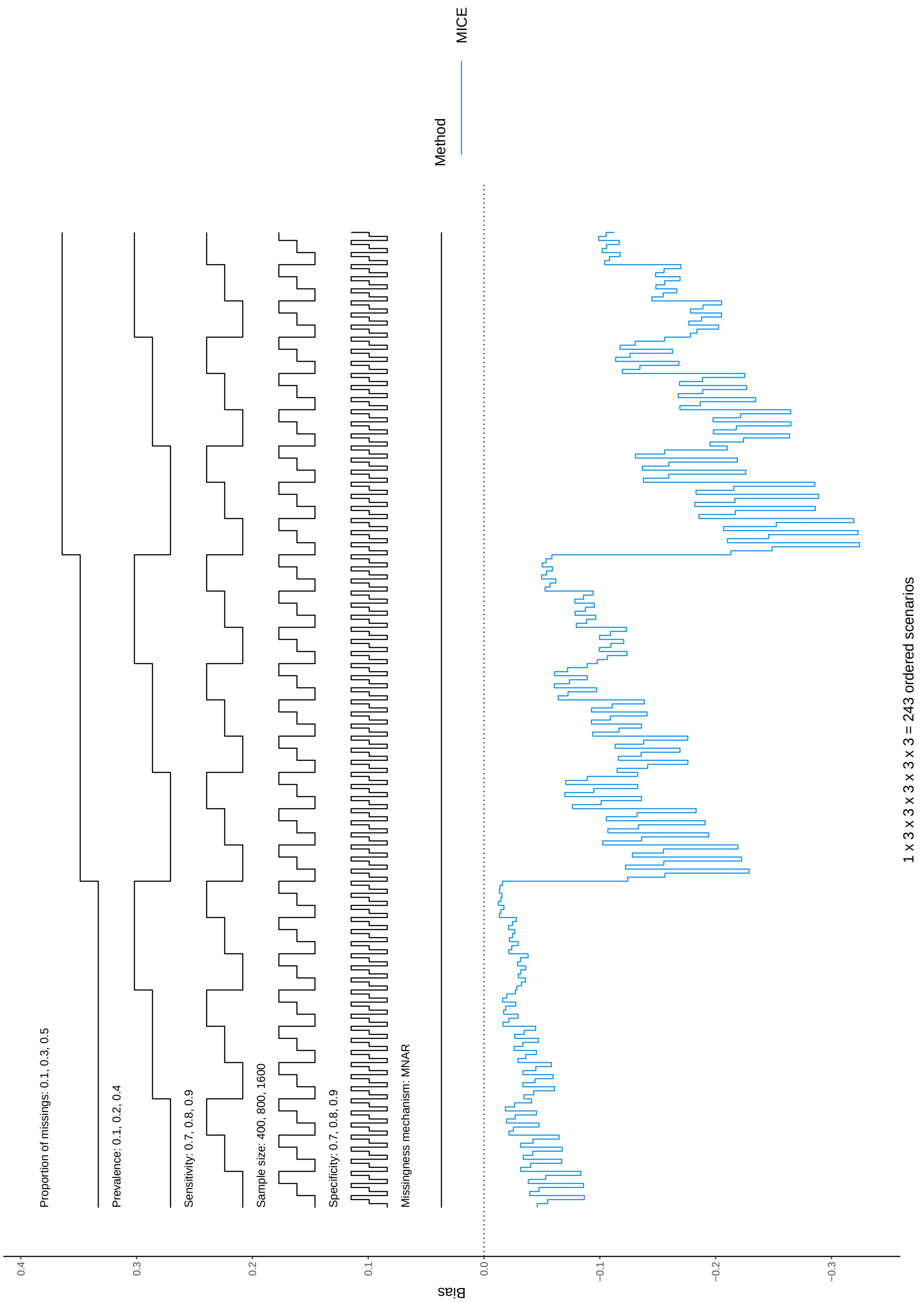

Fig. S13 Bias of sensitivity estimates of MICE with m = 5 across the distinct scenarios under MNAR

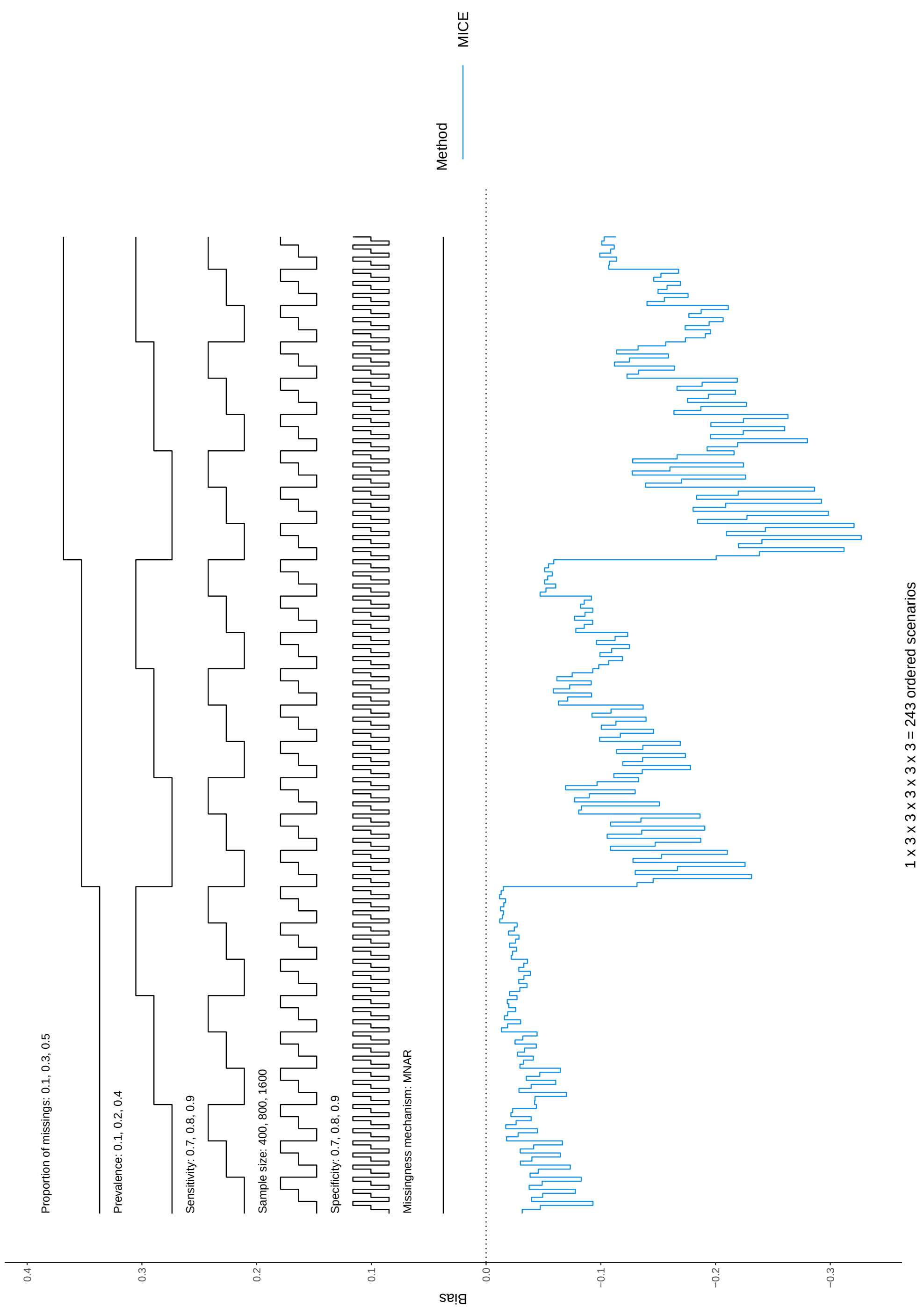

Fig. S14 Bias of sensitivity estimates of MICE with m = 50 across the distinct scenarios under MNAR

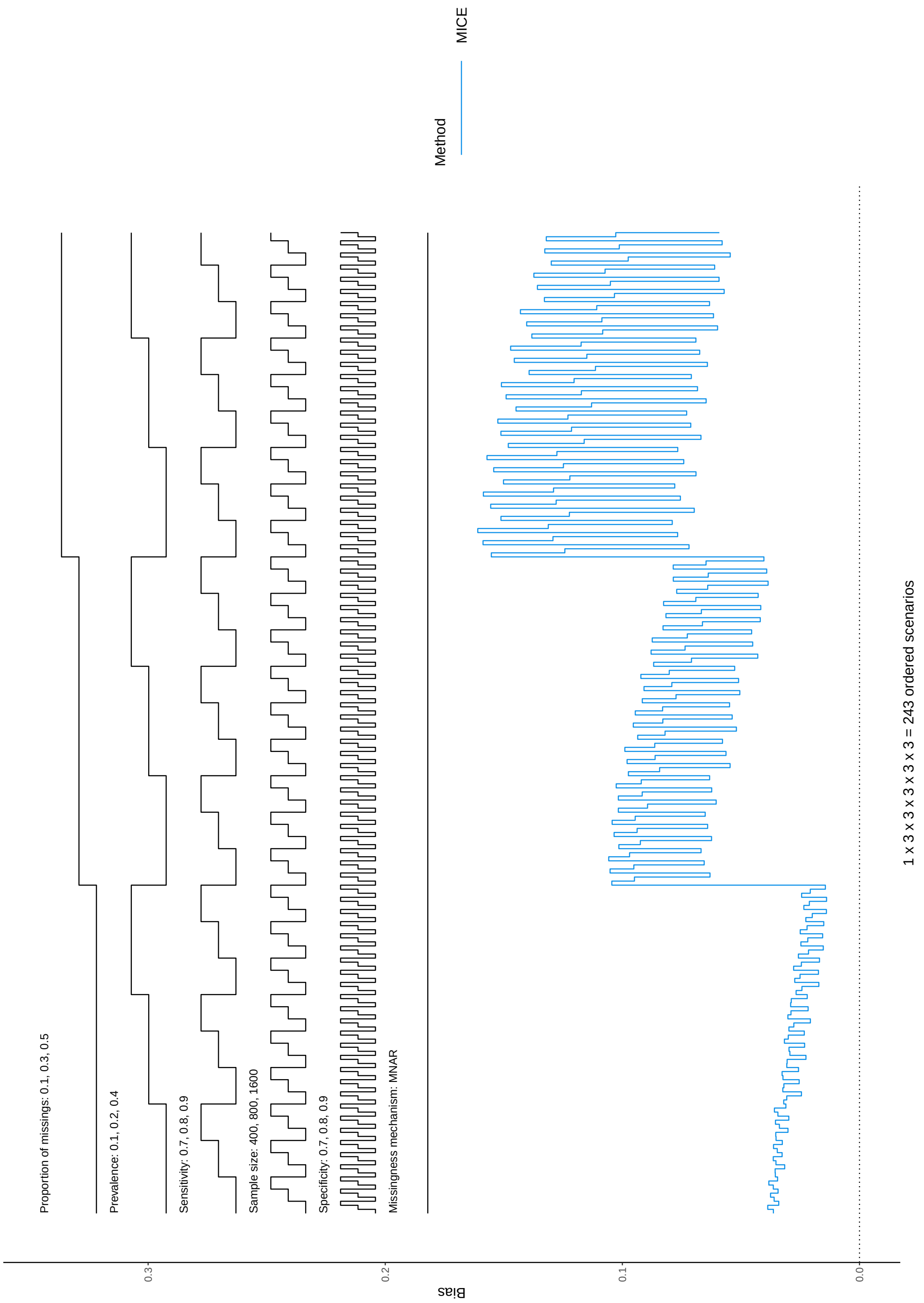

Fig. S15 Bias of specificity estimates of MICE with m = 5 across the distinct scenarios under MNAR

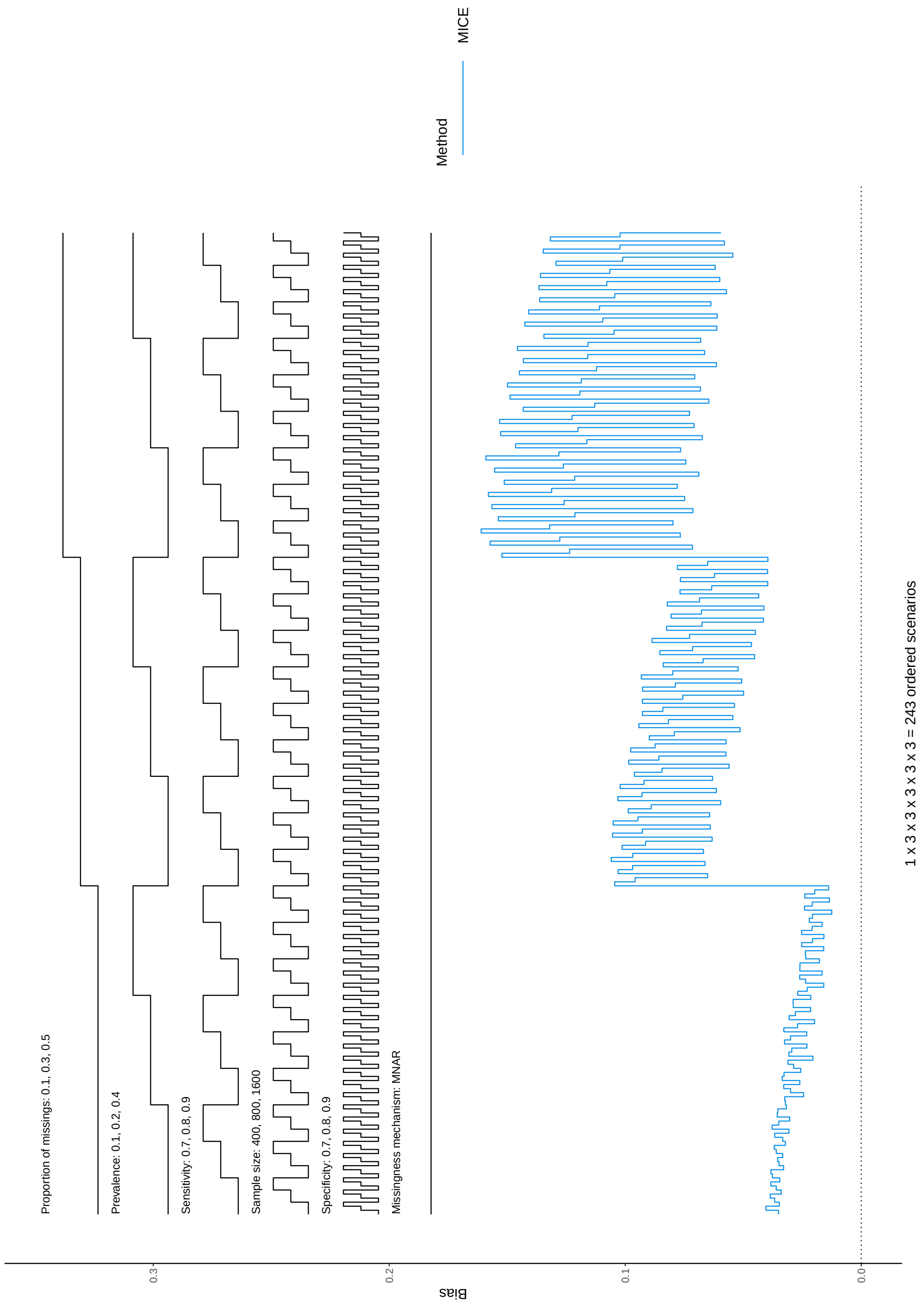

Fig. S16 Bias of specificity estimates of MICE with m = 50 across the distinct scenarios under MNAR

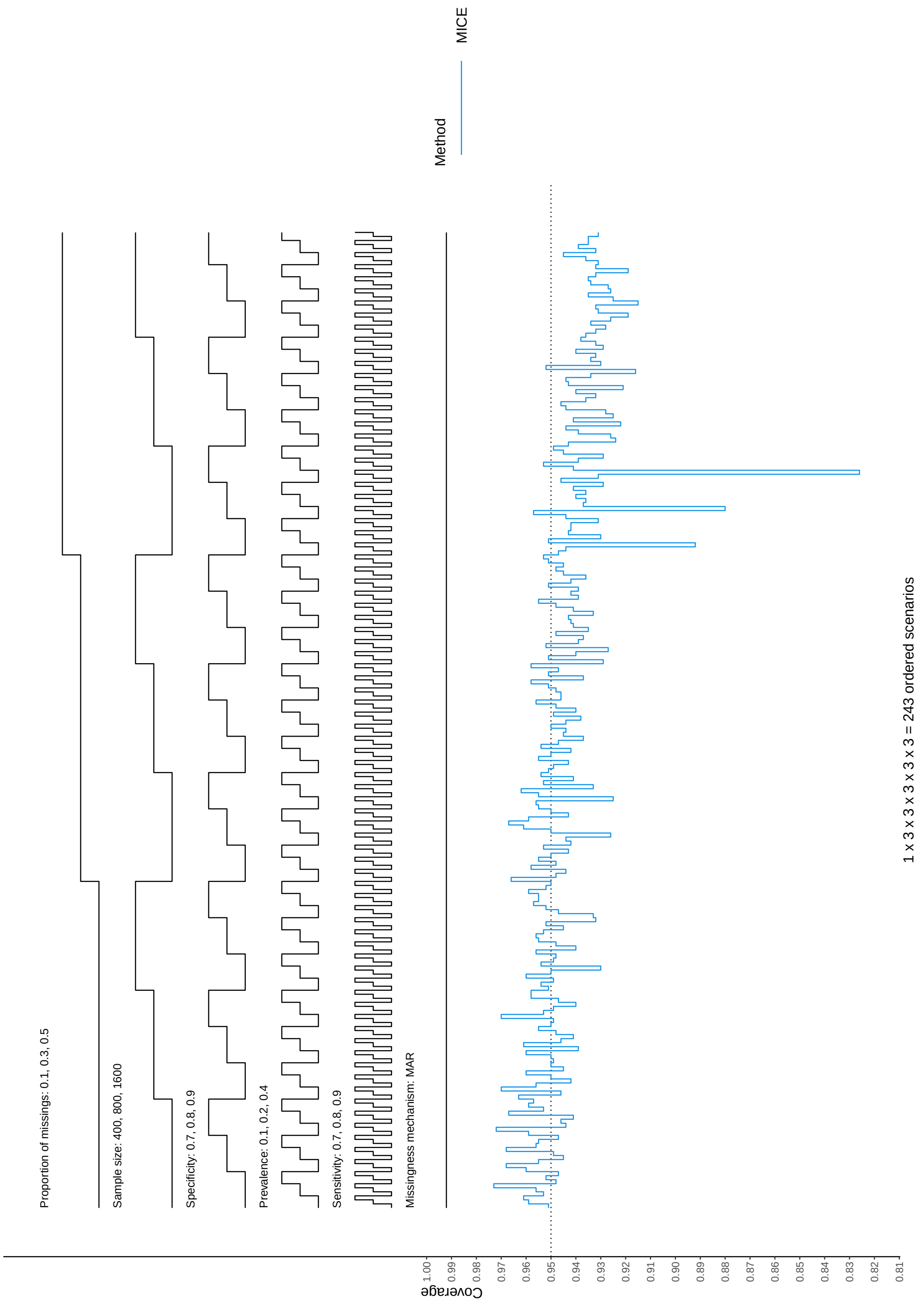

Fig. S17 Logit coverage probability for sensitivity estimates of MICE with m = 5 across the distinct scenarios under MAR

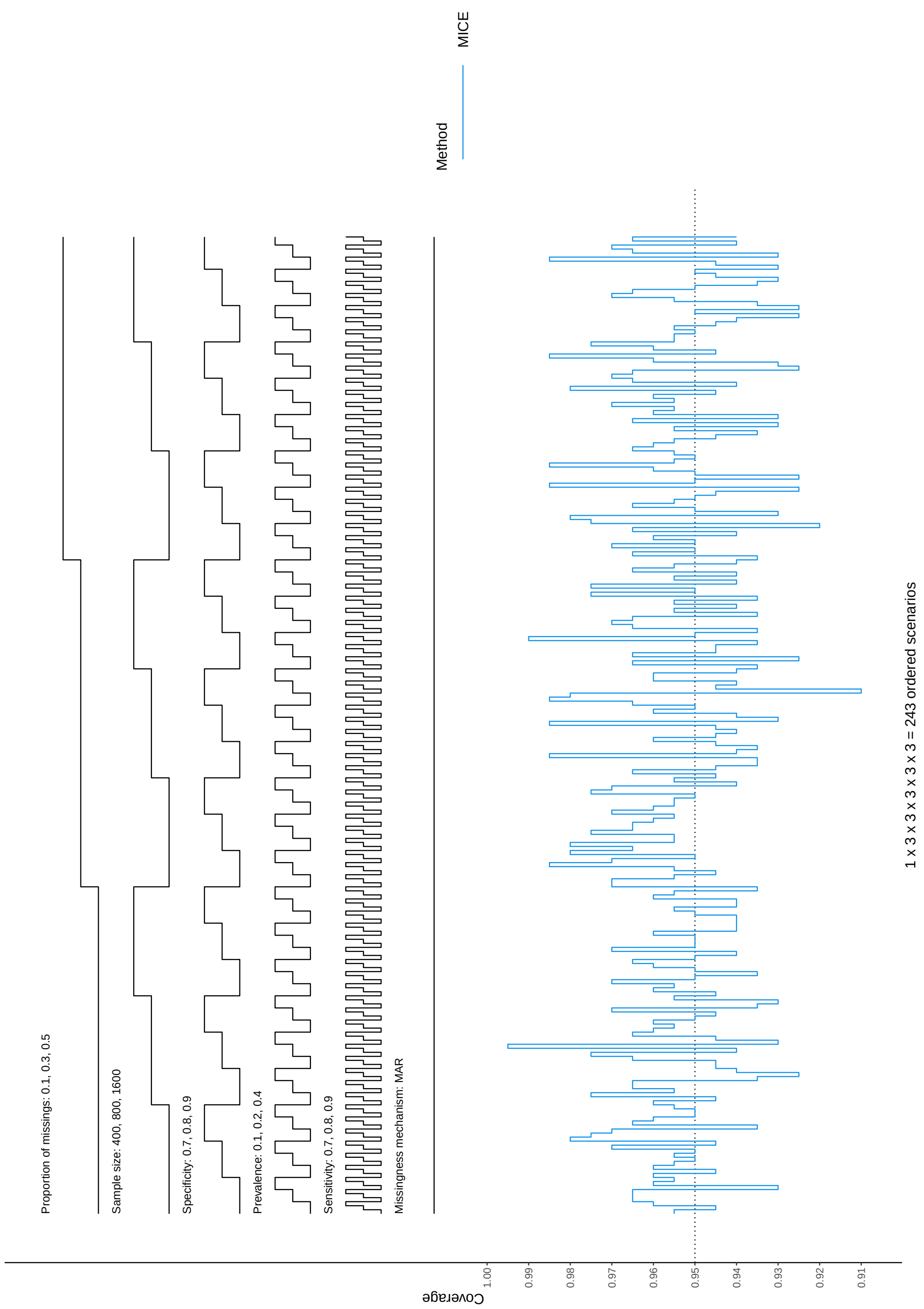

Fig. S18 Logit coverage probability for sensitivity estimates of MICE with m = 50 across the distinct scenarios under MAR

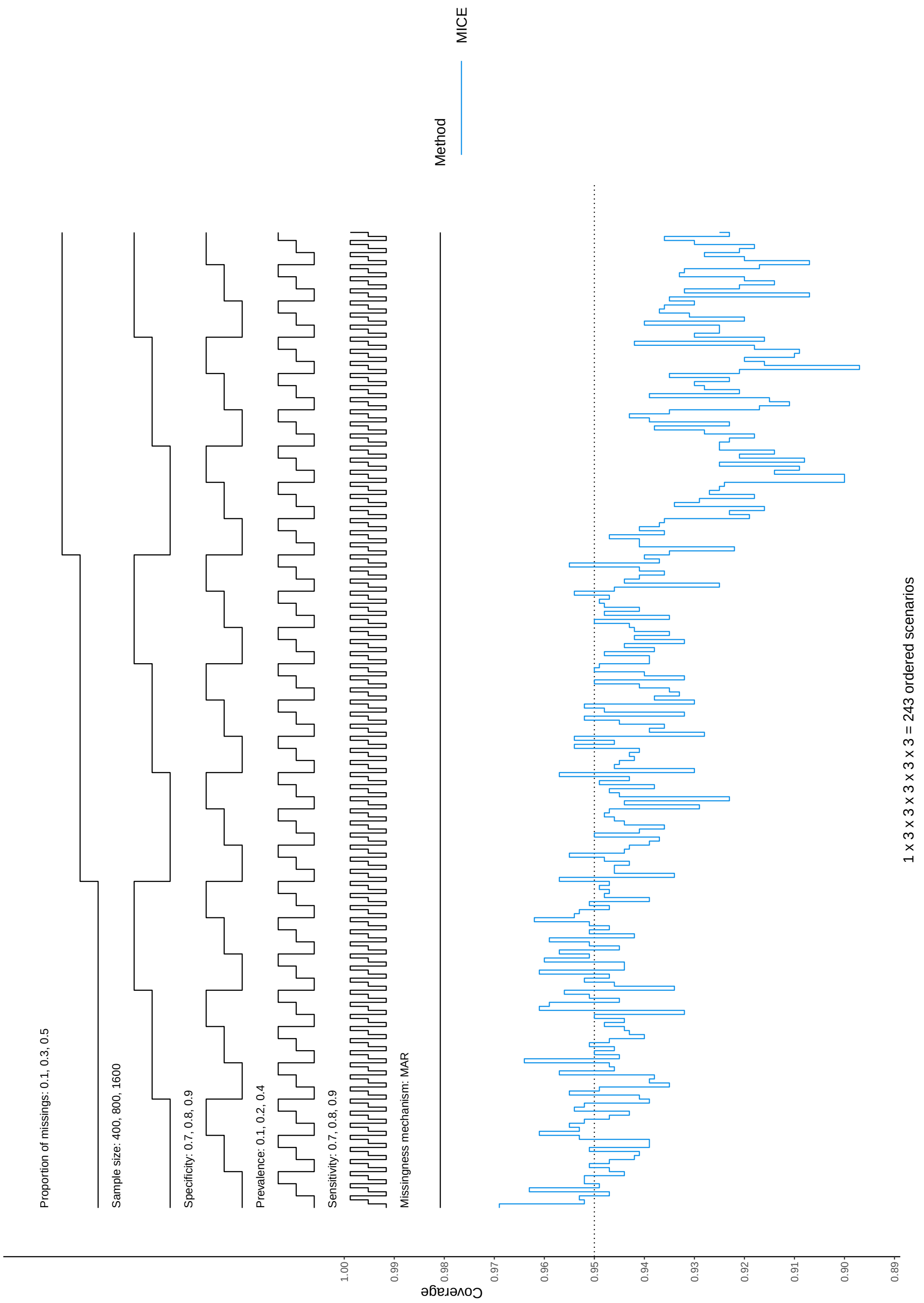

Fig. S19 Logit coverage probability for specificity estimates of MICE with m = 5 across the distinct scenarios under MAR

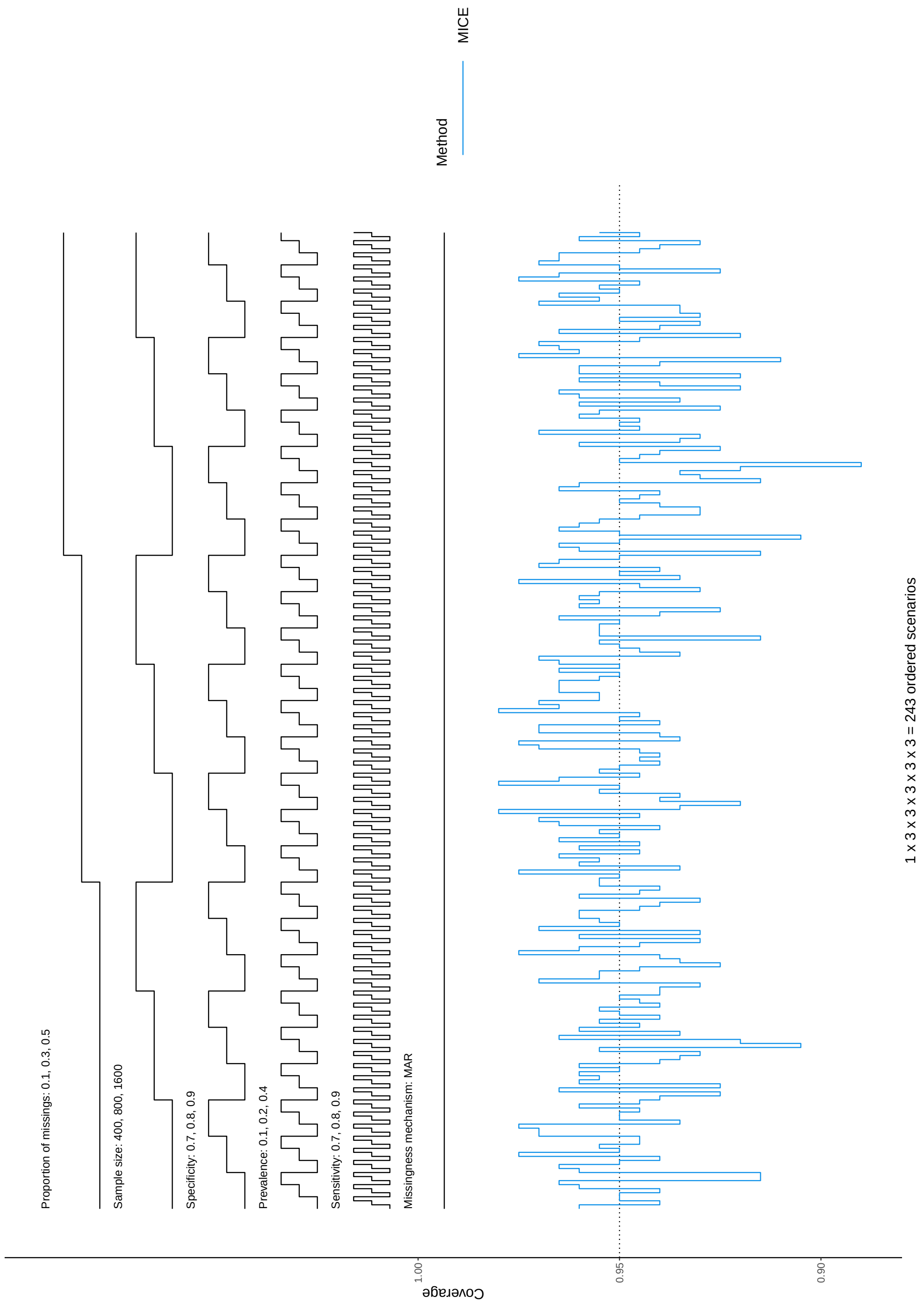

Fig. S20 Logit coverage probability for specificity estimates of MICE with m = 50 across the distinct scenarios under MAR

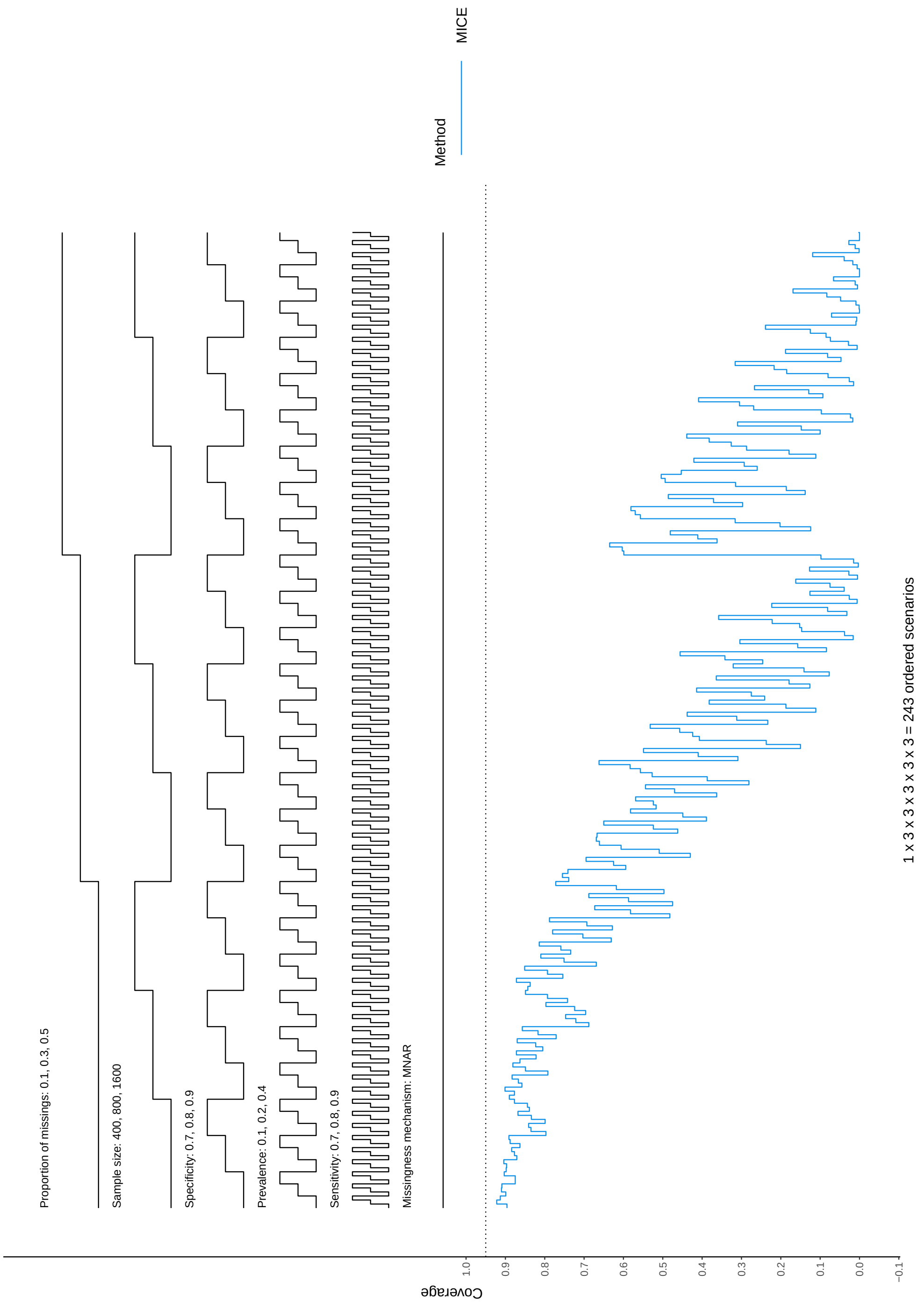

Fig. S21 Logit coverage probability for sensitivity estimates of MICE with m = 5 across the distinct scenarios under MNAR

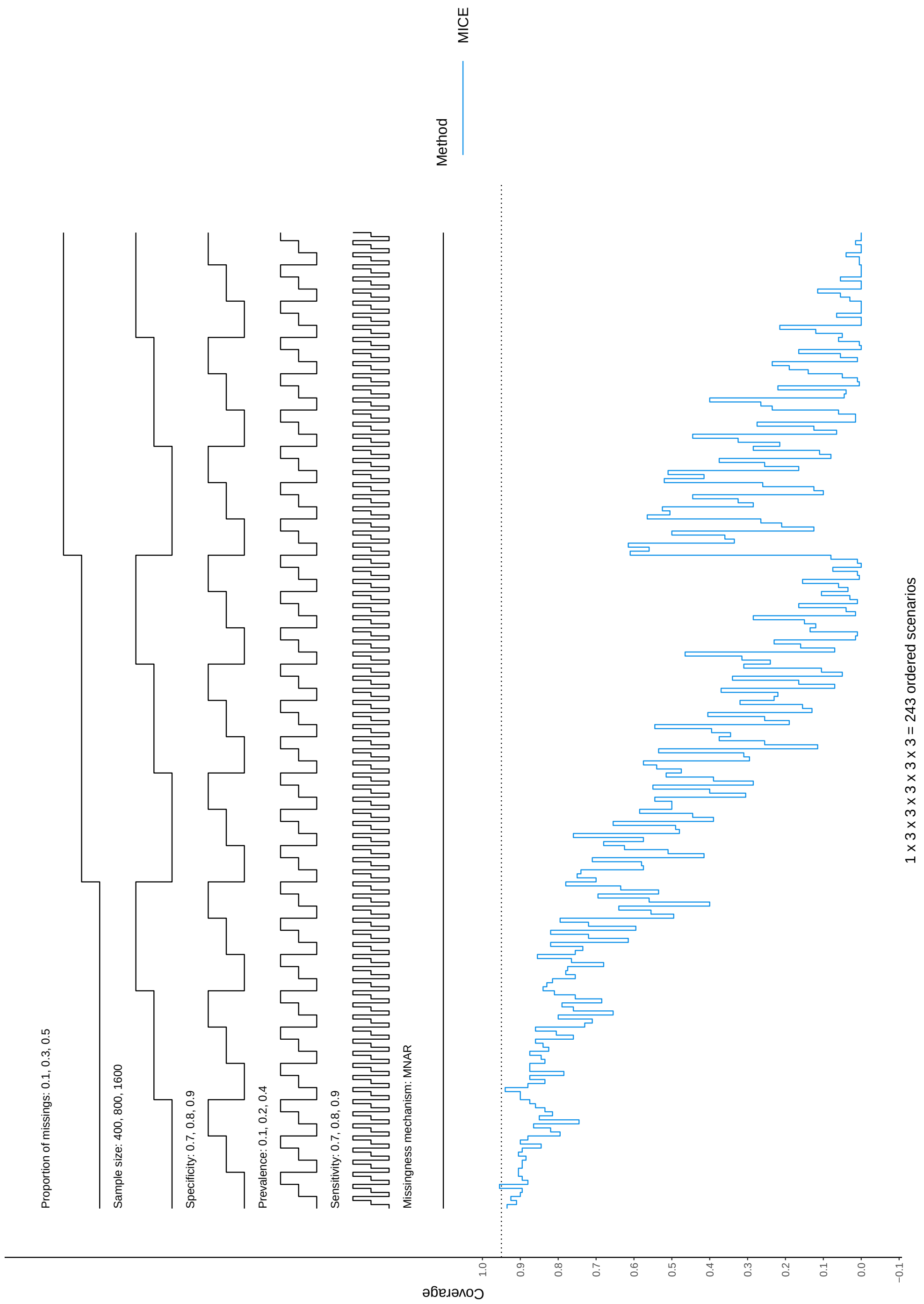

Fig. S22 Logit coverage probability for sensitivity estimates of MICE with m = 50 across the distinct scenarios under MNAR

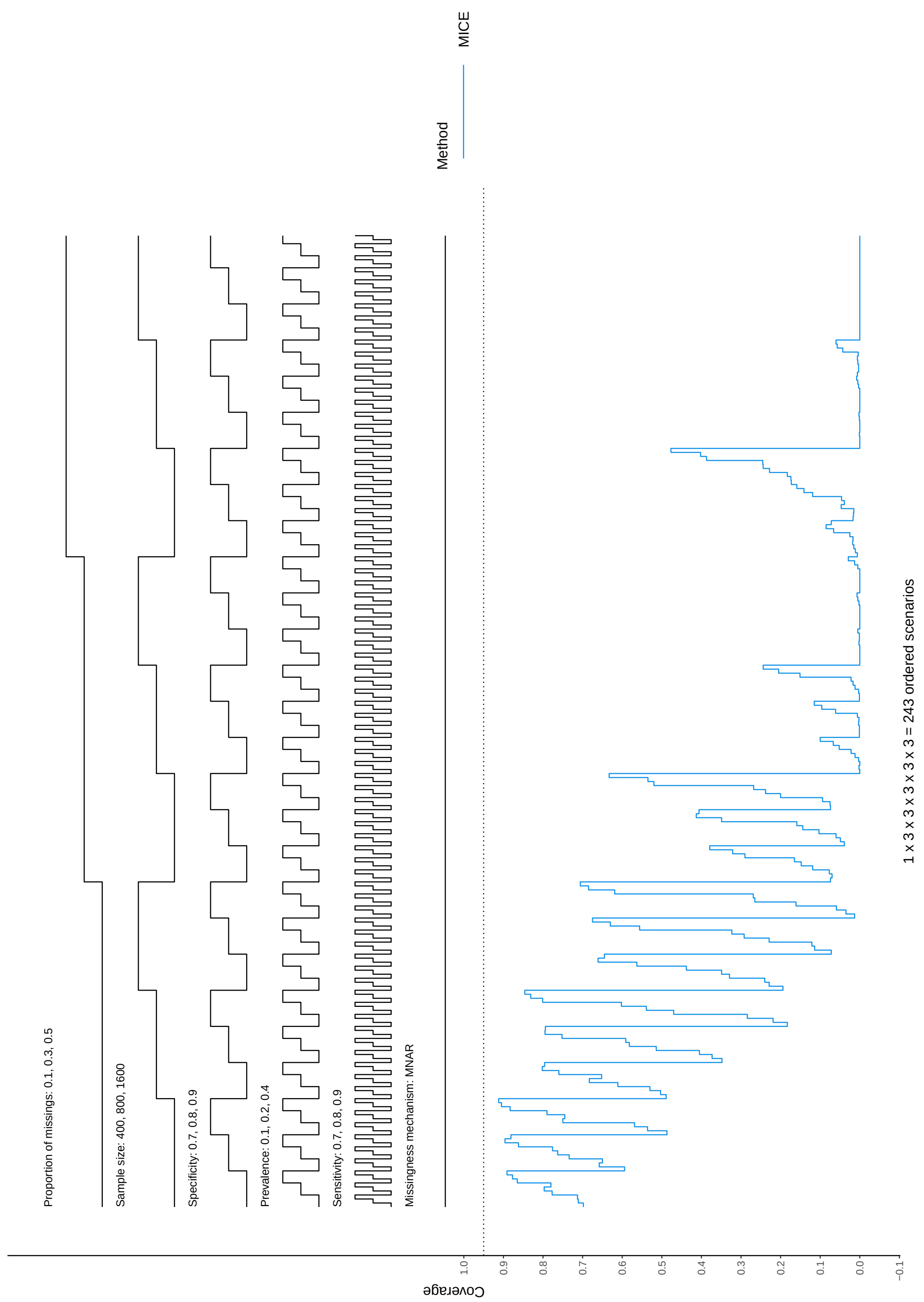

Fig. S23 Logit coverage probability for specificity estimates of MICE with m = 5 across the distinct scenarios under MNAR

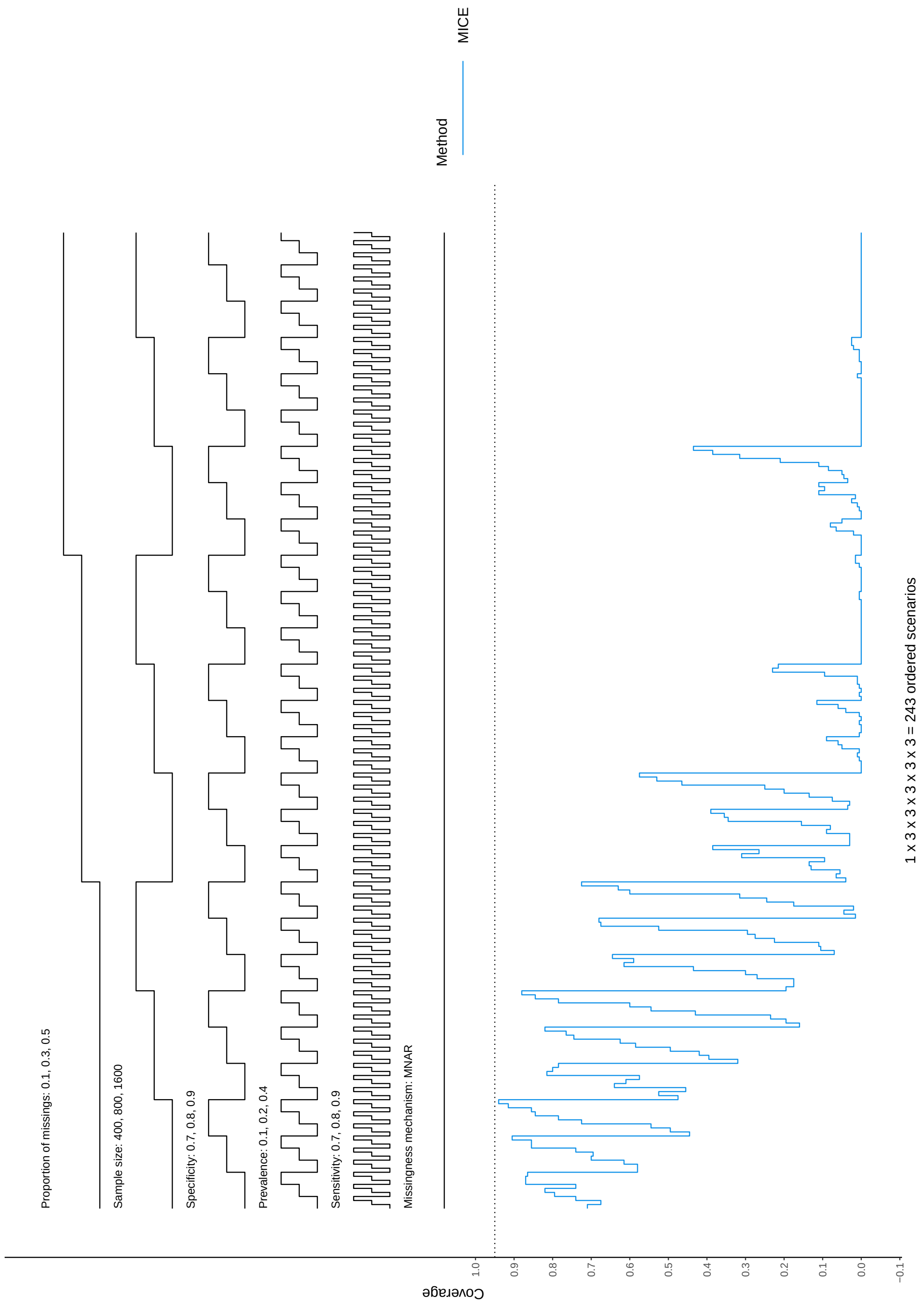

Fig. S24 Logit coverage probability for specificity estimates of MICE with m = 50 across the distinct scenarios under MNAR

2 Monte Carlo Standard Errors

Table 1 Monte Carlo Error for Bias

Measure of Accuracy	Average	Median	Minimum	Maximum
Sensitivity	0.0015	0.0013	0.0004	0.0085
Specificity	0.0006	0.0006	0.0002	0.0017

Table 2 Monte Carlo Error for Coverage

Measure of Accuracy	Average	Median	Minimum	Maximum
Sensitivity	0.0089	0.0091	0	0.0158
Specificity	0.0077	0.0080	0	0.0158

Table 3 Monte Carlo Error for MSE

Measure of Accuracy	Average	Median	Minimum	Maximum
Sensitivity	0.0004	0.0002	6.6247e-06	0.0063
Specificity	7.4780e-05	3.3889e-05	3.0424e-06	0.0009

Table 4 Monte Carlo Error for EmpSE

Measure of Accuracy	Average	Median	Minimum	Maximum
Sensitivity	0.0011	0.0009	0.0003	0.0060
Specificity	0.0004	0.0004	0.0001	0.0012

Table 5 Monte Carlo Error for Power

Measure of Accuracy	Average	Median	Minimum	Maximum
Sensitivity	0.0067	0.0052	0	0.0158
Specificity	0.0021	0	0	0.0158

Table 6 Monte Carlo Error for Bias (MICE with M = 50)

Measure of Accuracy	Average	Median	Minimum	Maximum
Sensitivity	0.0034	0.0028	0.0009	0.0111
Specificity	0.0013	0.0012	0.0004	0.0030

3 Mean Squared Error (MSE)

In Figure S25 it can be seen that the MSE pattern for sensitivity under MCAR has similarities to the bias pattern for sensitivity under MCAR: The MSE values for all methods except the worst case method are relatively low, but tend to increase with an increase in the proportion of missings. Additionally, MSE values tend to be higher for smaller prevalences, which can be explained by the fact that lower prevalences lead to lower sample sizes for sensitivity estimation.

Figure S26 illustrates that the MSE values for specificity estimates are low for all methods except for the worst case method. This indicates that specificity estimates under MCAR not only have low bias but also high precision, higher than that for sensitivity estimates. This can be explained by the fact that the sample size that specificity is estimated on is larger than the sample size that sensitivity is estimated on.

The MSE pattern for sensitivity under MAR is similar to the MSE pattern for sensitivity under MCAR (Figure S27), with slightly larger MSE values. High missingness and low prevalence indicate higher MSE. For specificity, the MAR MSE pattern differs from the MCAR MSE pattern (Figure S28): MSE increases with higher missingness rates.

Figure S29 and Figure S30 show the MSE for sensitivity and specificity under MNAR. For a missingness proportion of 0.1, the MSE is relatively low. Combined with the low bias, most methods seem to perform relatively well even under MNAR when there are only few missings. Other than that, the MSE under MNAR is noticeably

Table 7 Monte Carlo Error for Coverage (MICE with M = 50)

Measure of Accuracy	Average	Median	Minimum	Maximum
Sensitivity	0.0088	0.0075	0	0.0158
Specificity	0.0069	0.0069	0	0.0158

higher for both sensitivity and specificity compared to MCAR and MAR. Monte Carlo Errors are fairly low here, indicating a precise estimation (Table 3).

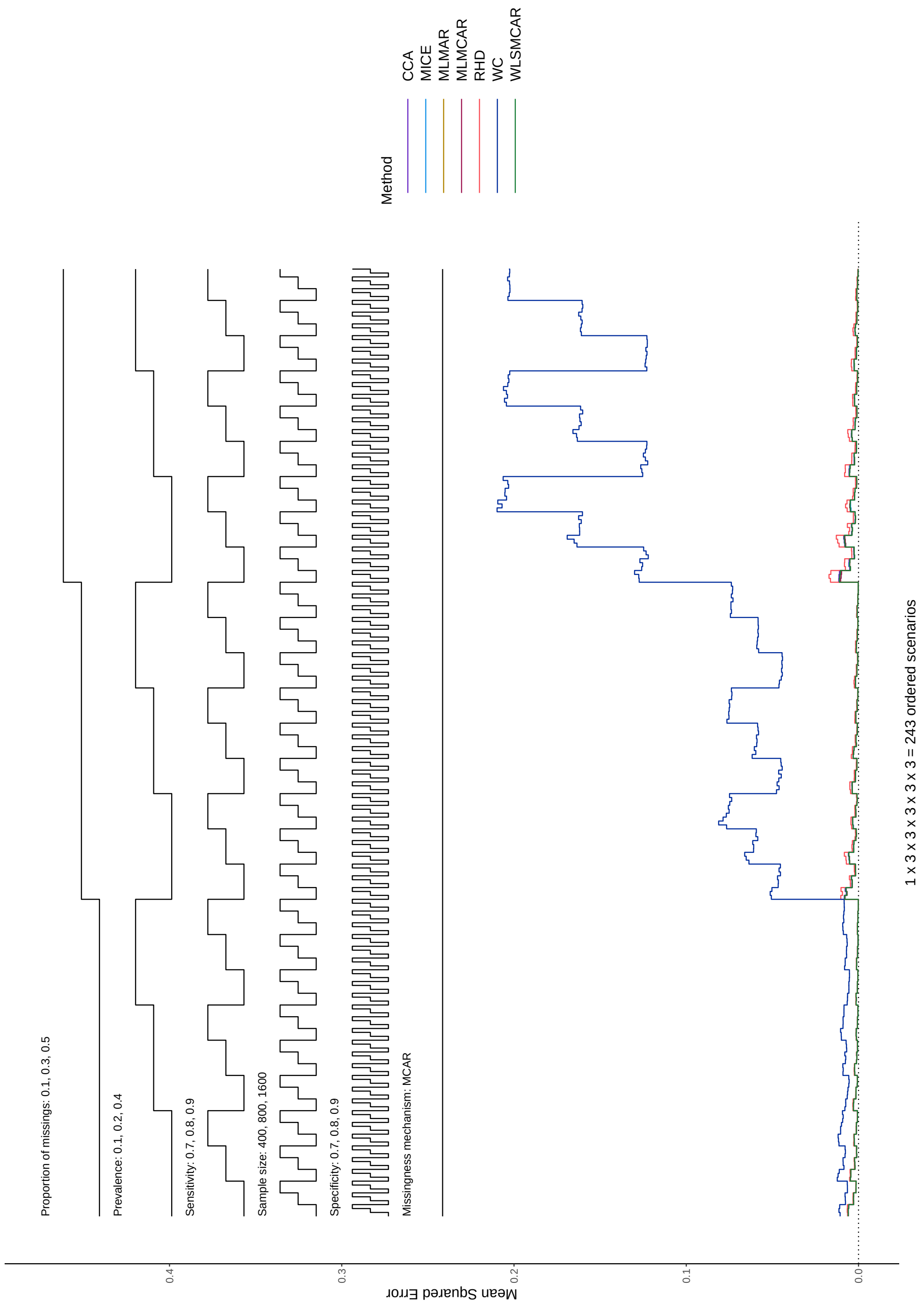

Fig. S25 MSE for sensititvity estimates of all methods across the distinct scenarios under MCAR

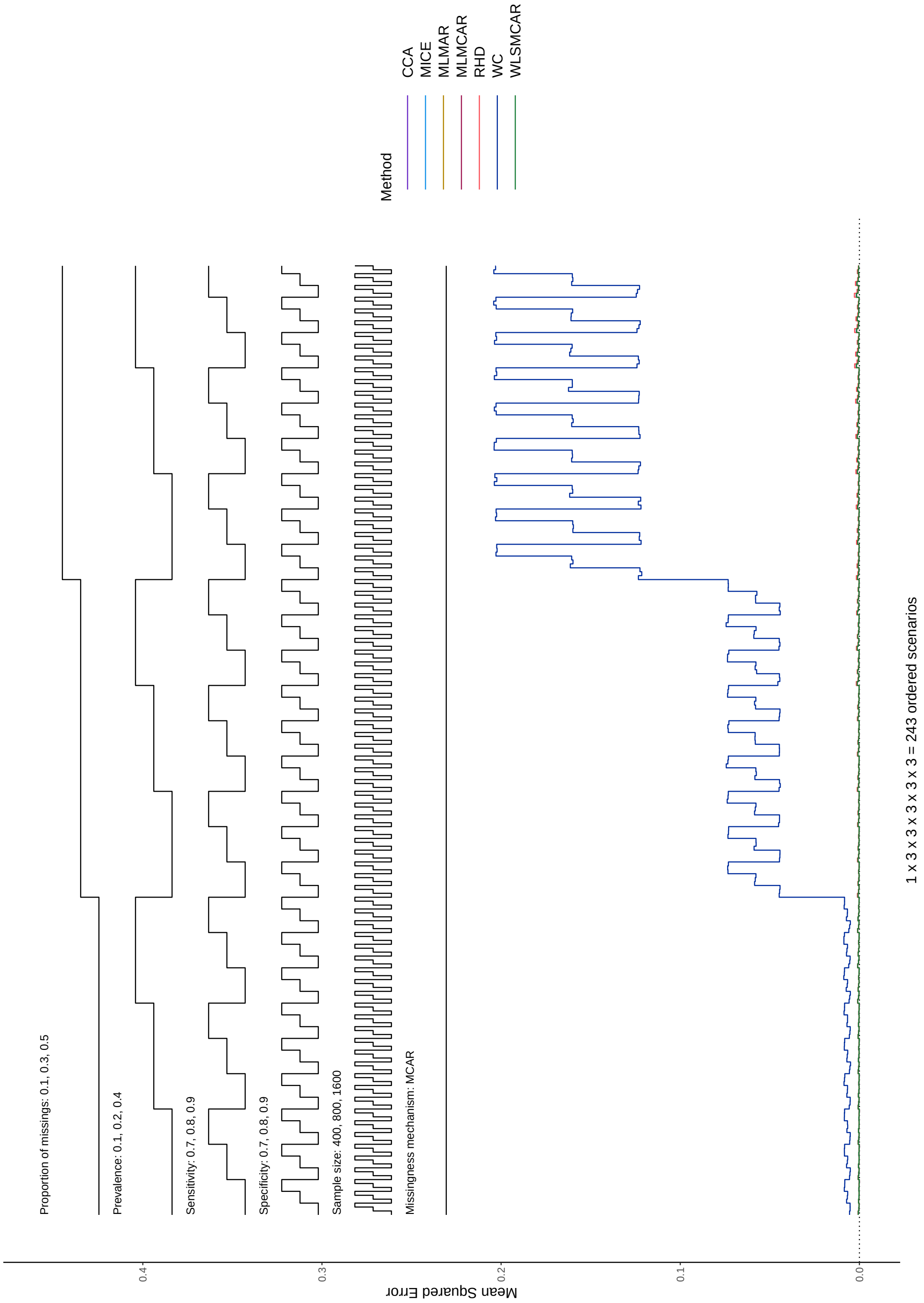

Fig. S26 MSE for specificity estimates of all methods across the distinct scenarios under MCAR

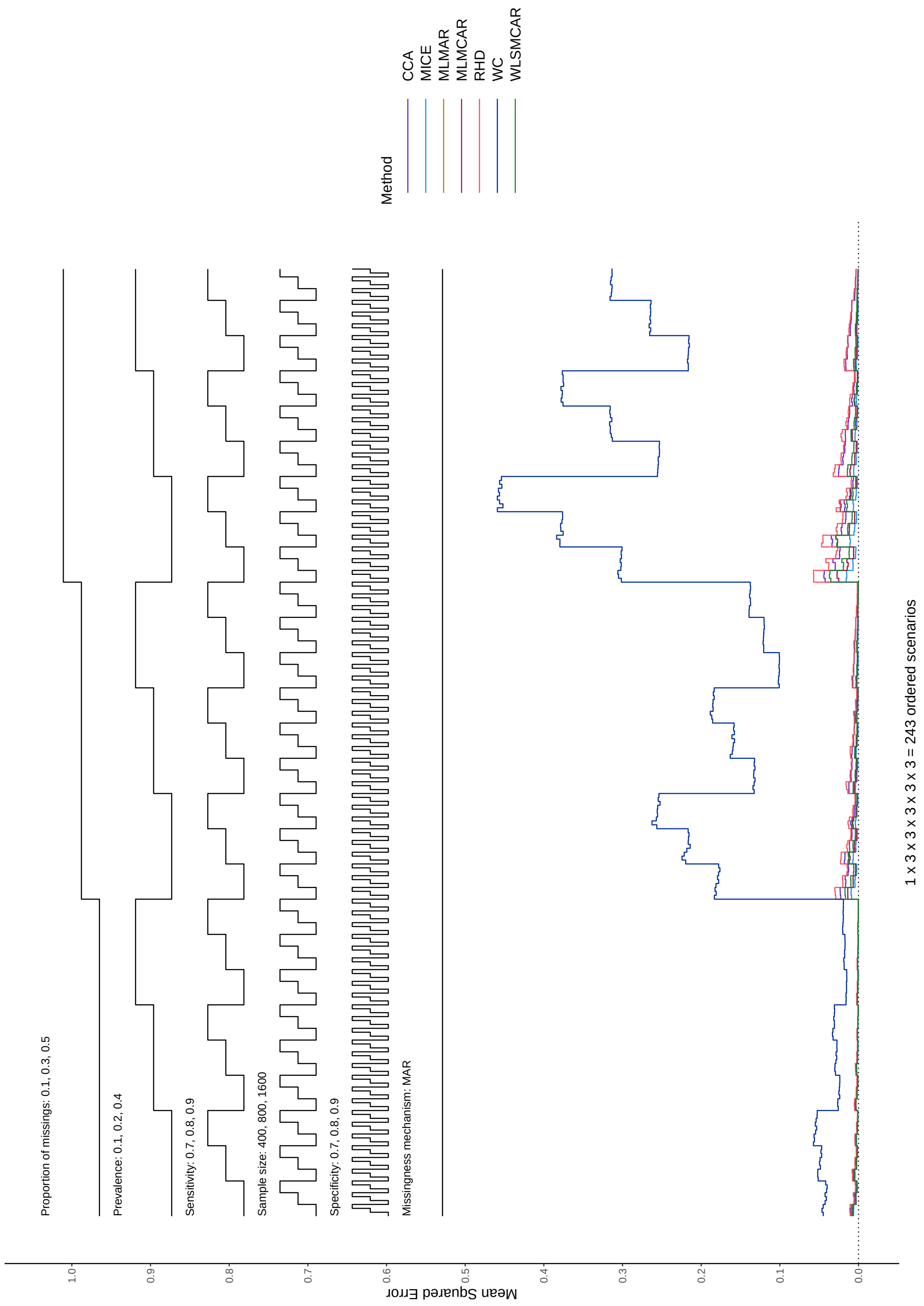

Fig. S27 MSE for sensititvity estimates of all methods across the distinct scenarios under MAR

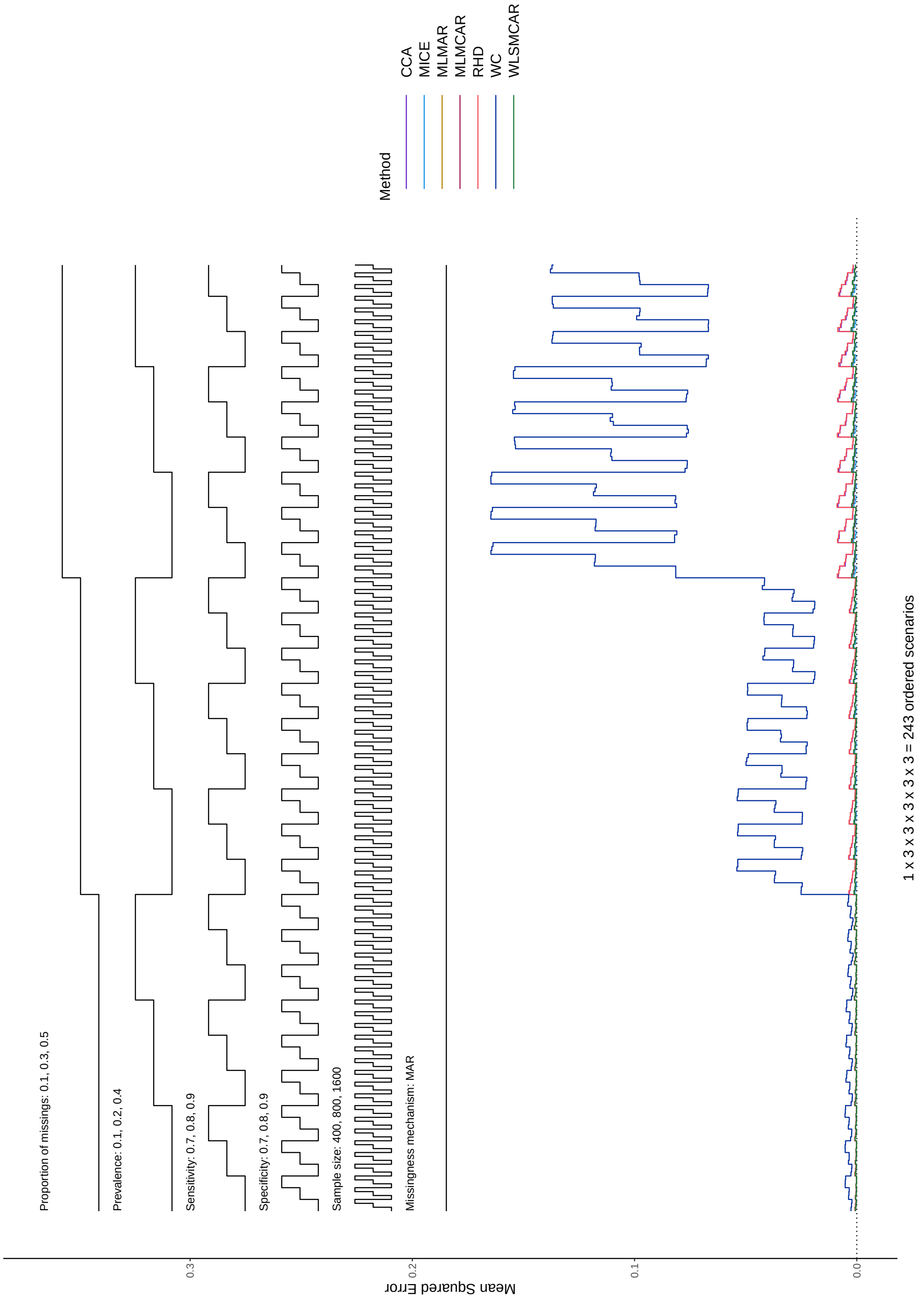

Fig. S28 MSE for specificity estimates of all methods across the distinct scenarios under MAR

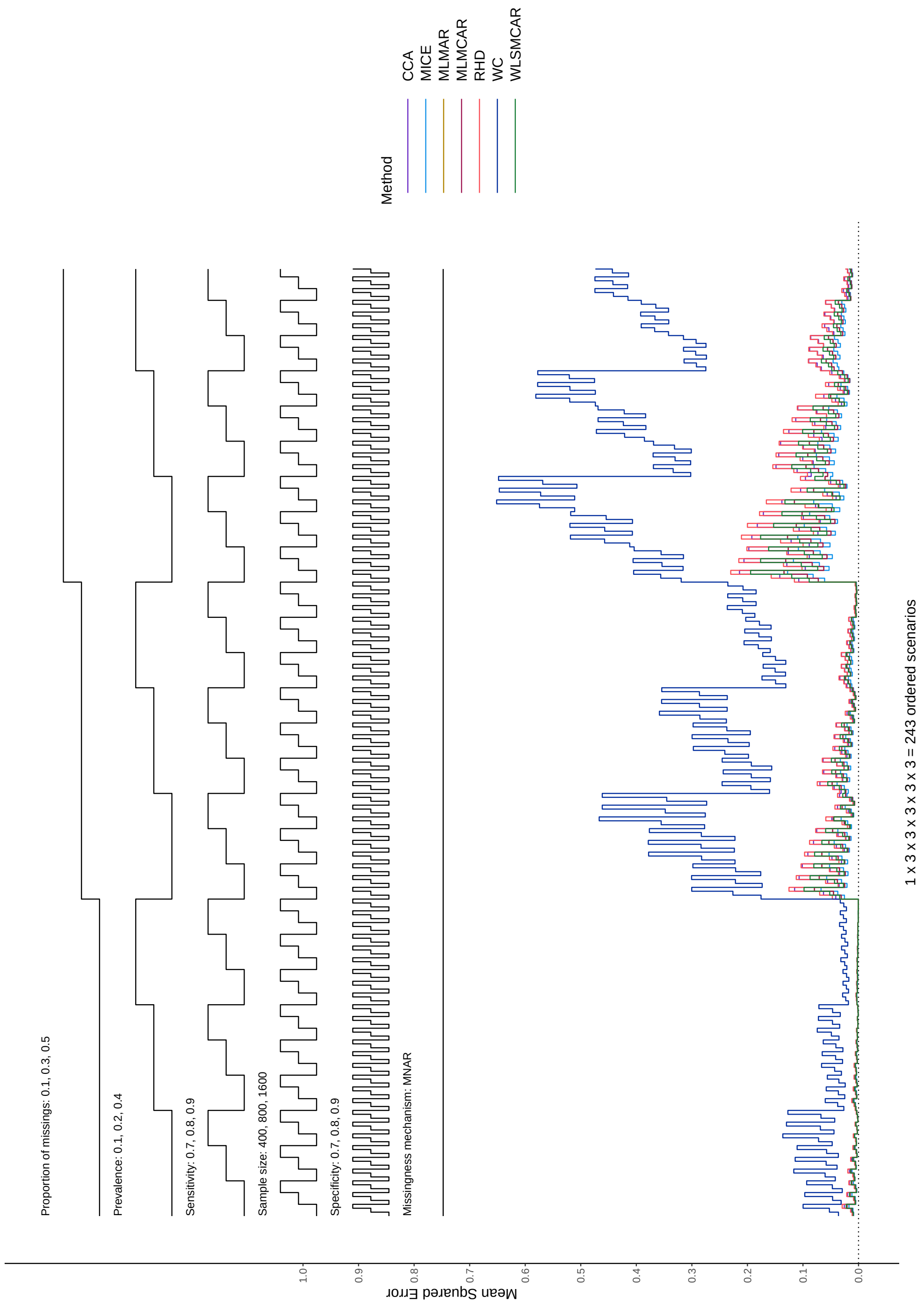

Fig. S29 MSE for sensititvity estimates of all methods across the distinct scenarios under MNAR

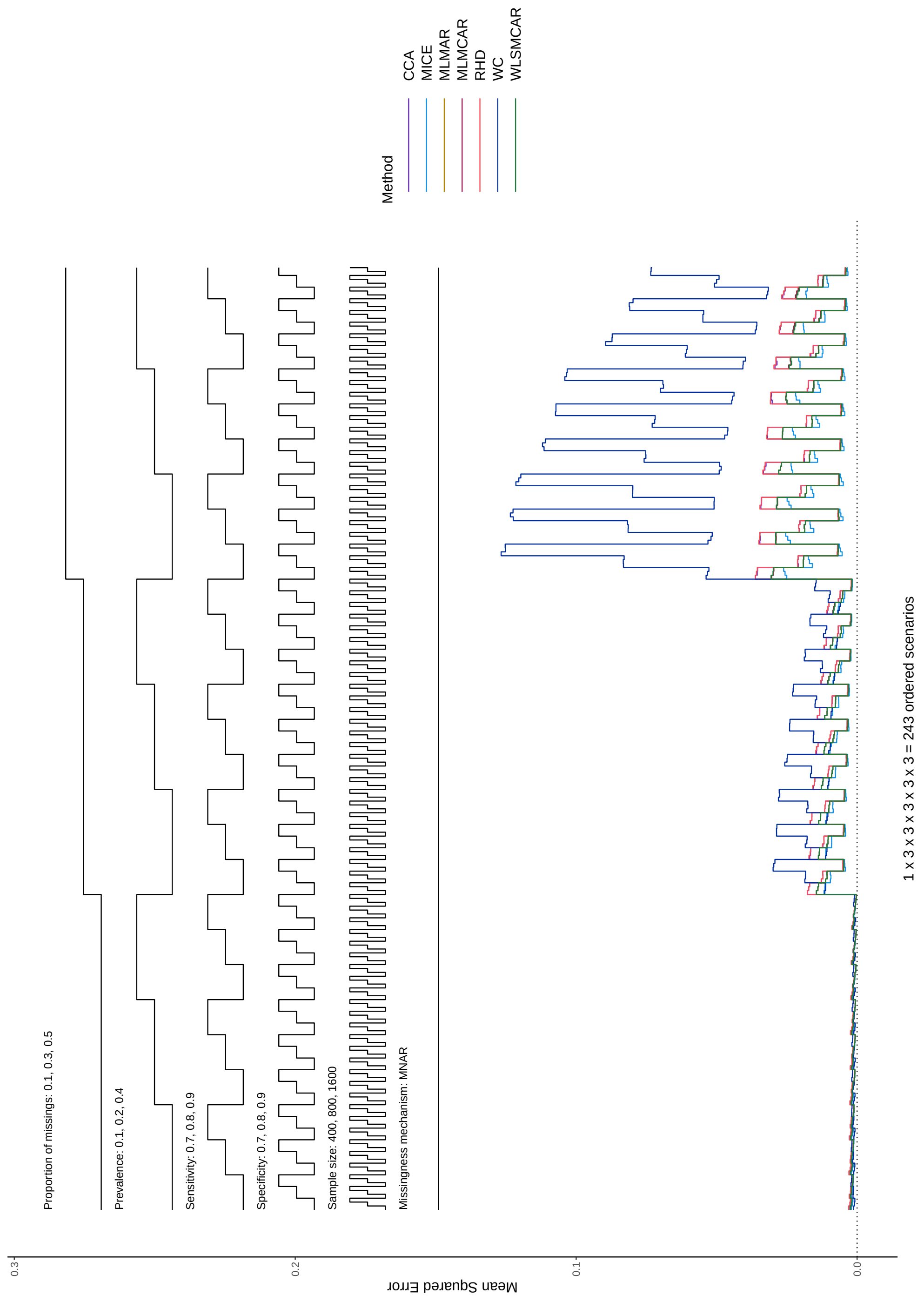

Fig. S30 MSE for specificity estimates of all methods across the distinct scenarios under MNAR

4 Empirical Standard Error

Across sensitivity and specificity, as well as all missingness mechanisms it can be seen that the empirical standard error tends to increase with an increase in the proportion of missings and tends to decrease with an increase in sample size (Figure S31, Figure S32, Figure S33, Figure S34, Figure S35, Figure S36). There are, however, differences that cannot necessarily be accounted for any parameter changing, instead, it speaks to the general volatility of the empirical standard error between scenarios. Additionally, while RHD tends to perform worst as it is the method with the highest empirical standard error out of every method, there is no clear best performer here. It is also not clear whether standard error differences in the magnitude of 0.01 are meaningful enough to serve as an argument for one method outperforming the other.

Again, the Monte Carlo Errors support the claim that the estimates in the simulation were efficiently estimated (Table 4).

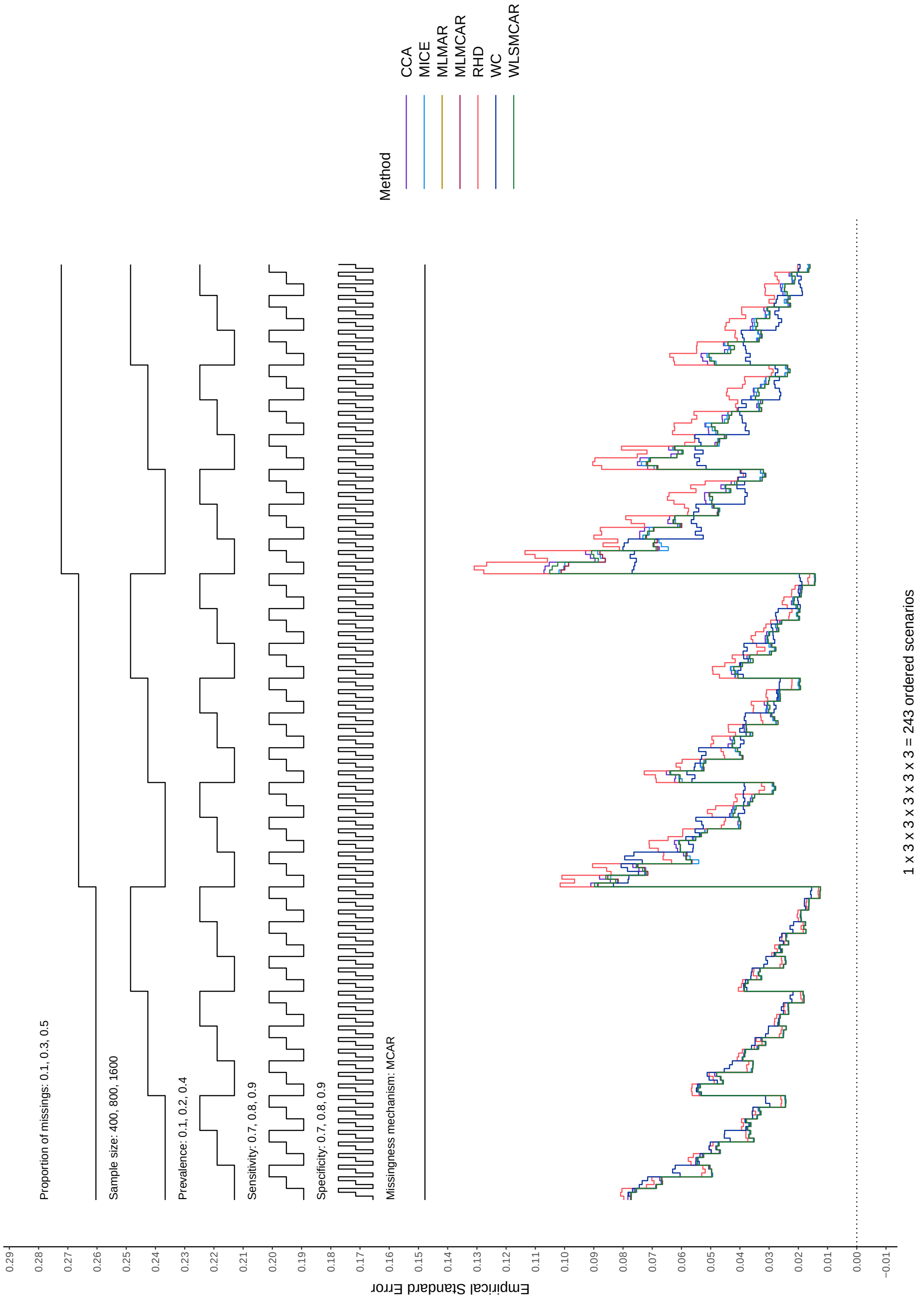

Fig. S31 EmpSE for sensitivity estimates of all methods across the distinct scenarios under MCAR

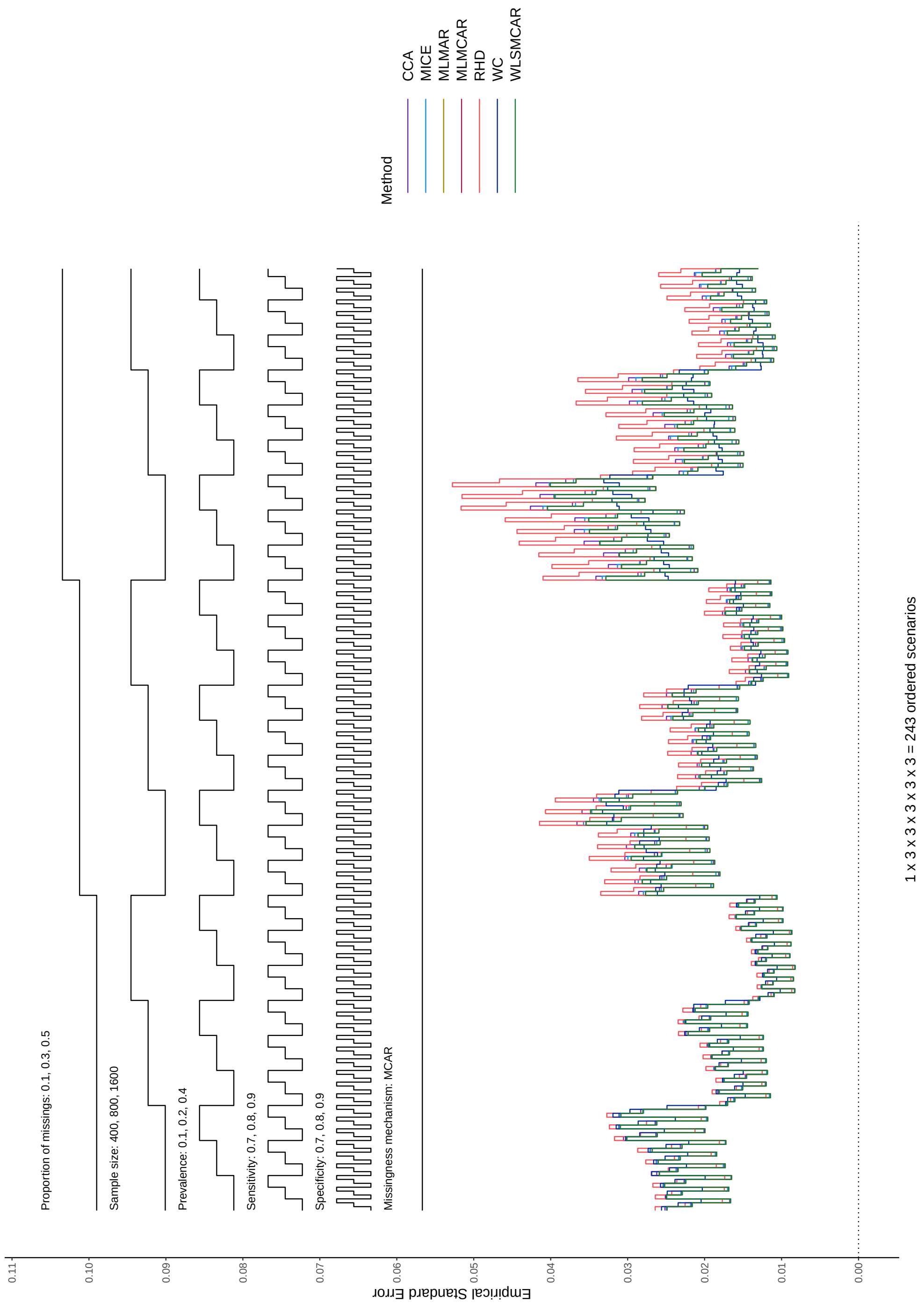

Fig. S32 EmpSE for specificity estimates of all methods across the distinct scenarios under MCAR

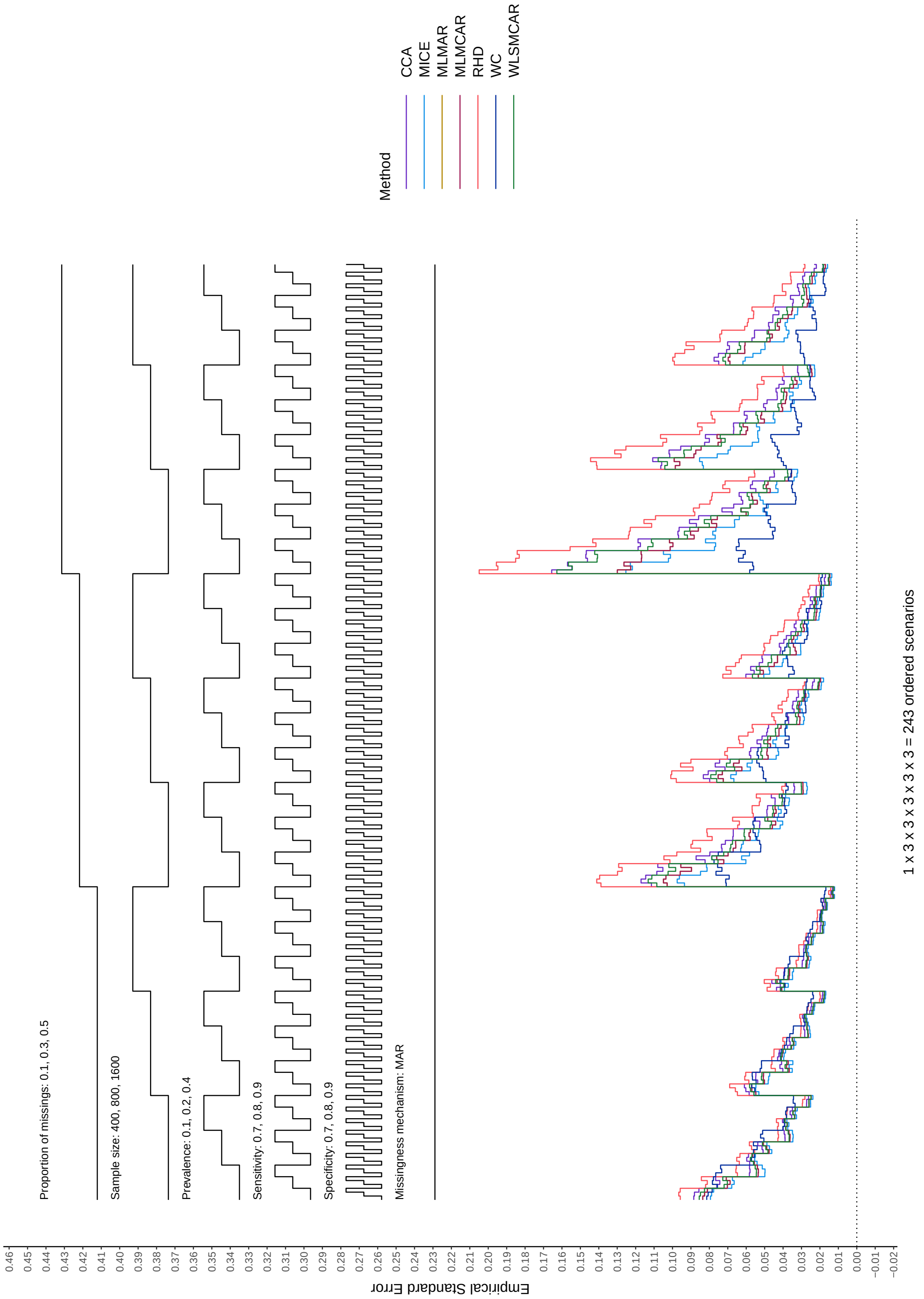

Fig. S33 EmpSE for sensitivity estimates of all methods across the distinct scenarios under MAR

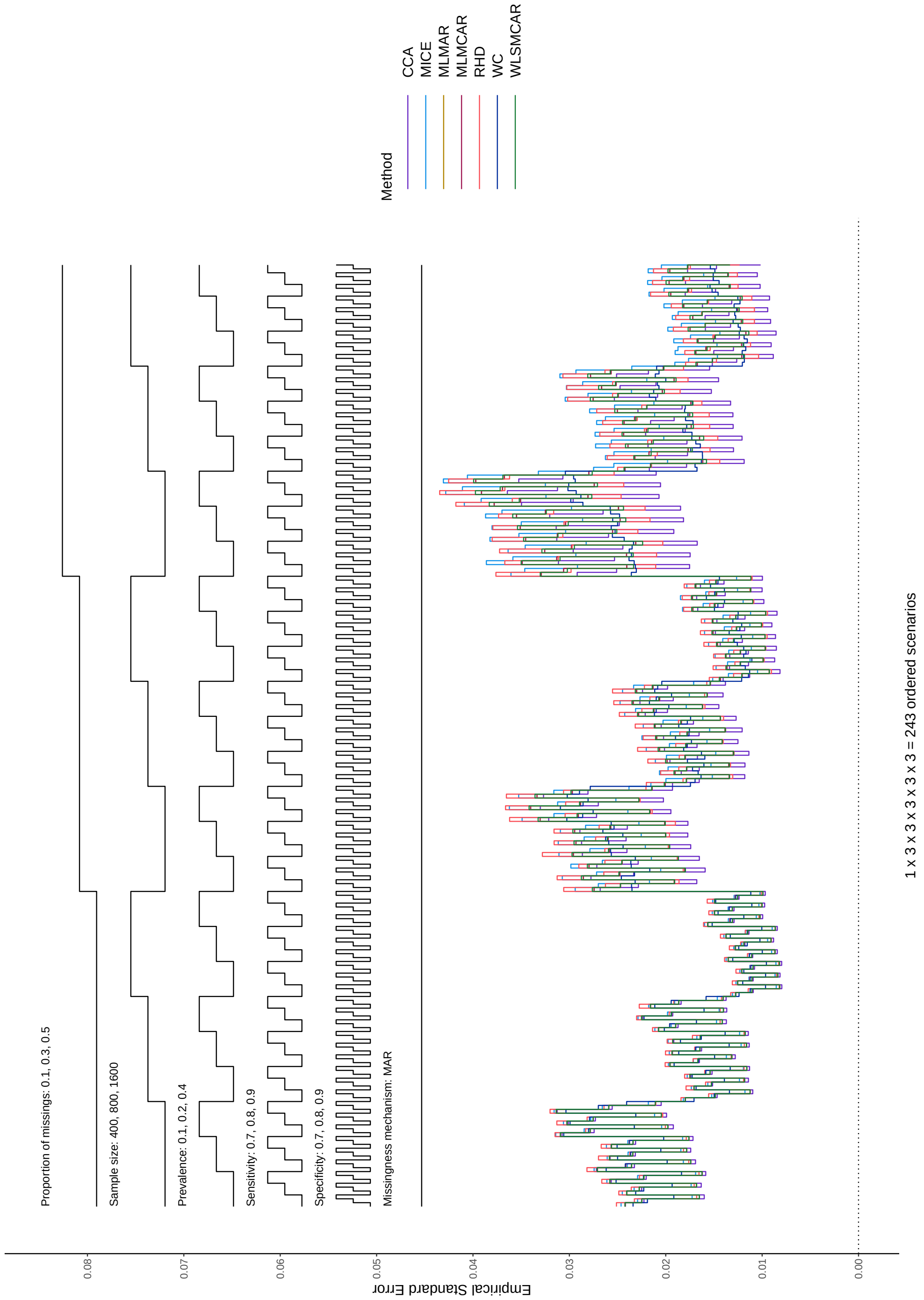

Fig. S34 EmpSE for specificity estimates of all methods across the distinct scenarios under MAR

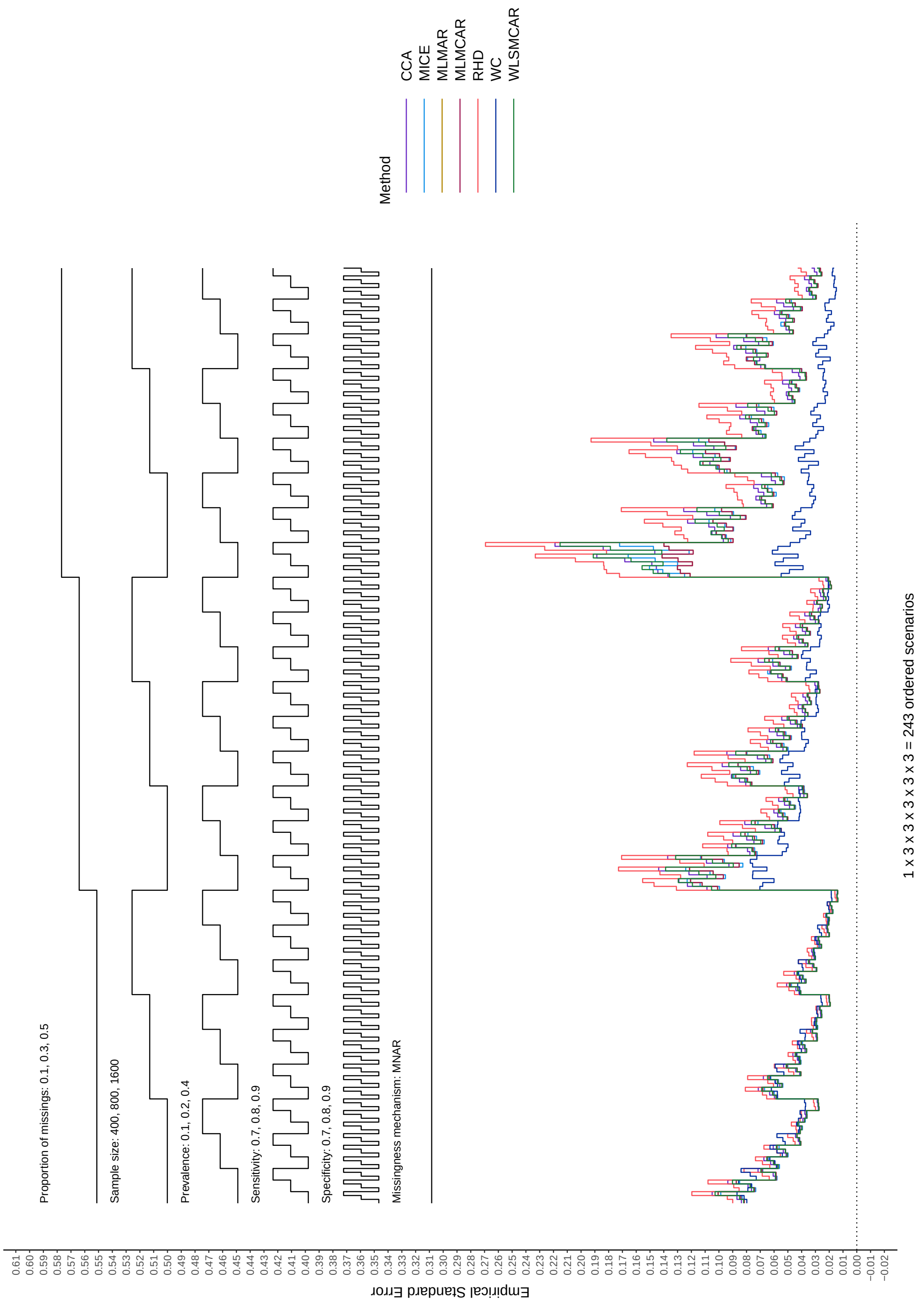

Fig. S35 EmpSE for sensitivity estimates of all methods across the distinct scenarios under MNAR

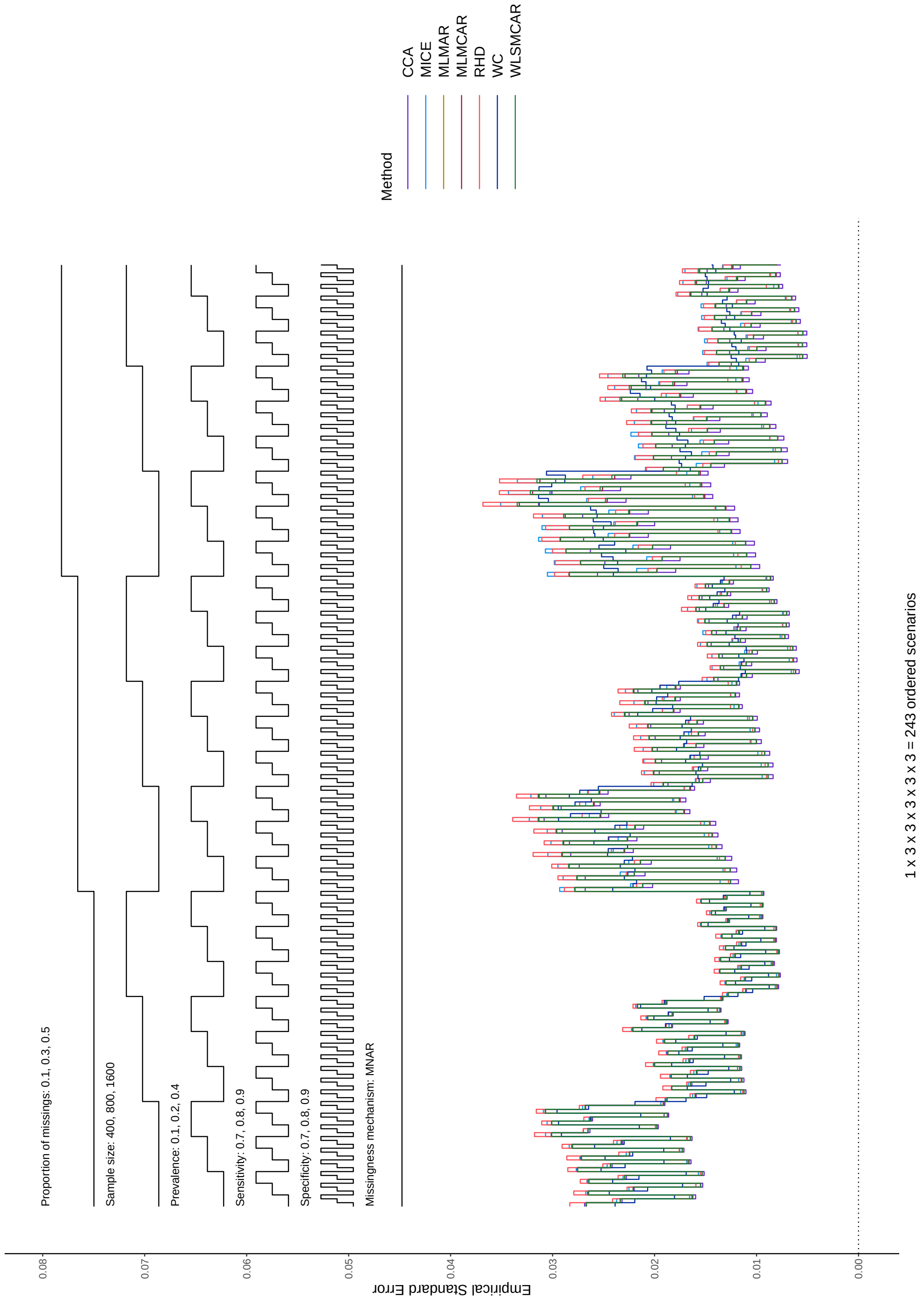

Fig. S36 EmpSE for specificity estimates of all methods across the distinct scenarios under MNAR

5 Power

Power analyses were only conducted for a few selected scenarios and methods that are unbiased and show good coverage. Since MICE is the only method that is unbiased and has good coverage under MCAR and MAR at least, power analyses were conducted for MICE. Additionally, power analyses for CCA were conducted as a reference. A scenario was chosen where the sensitivity and specificity are 0.8, the sample size is 800, the prevalence is 0.2 and the proportion of missings is 0.5. This scenario represents a kind of average or baseline scenario (with high missingness), which is helpful in providing insights into the power of MICE and CCA while also not being overbearing concerning the number of scenarios. Power analyses for this scenario were conducted under all missingness mechanisms.

For MCAR, the power of MICE is not consistently larger than the power of CCA for sensitivity estimates (Figure S37), while the power of MICE is consistently smaller than the power of CCA for specificity estimates (Figure S38). For MAR, the power of MICE is, this time, consistently larger than the power of CCA for sensitivity estimates (Figure S39), while the power of MICE is consistently smaller than the power of CCA for specificity estimates until both reach a power of 1 (Figure S40). For MNAR, a similar trend for the power of sensitivity estimates can be seen, with the difference being that the power is overall smaller than for other missingness mechanisms (Figure S41). For specificity estimates, the power is 1 in all cases (Figure S42).

The difference in power between CCA and MICE could be explained through bias. With a positive bias for specificity estimates, the null hypothesis is more likely to be rejected. With a negative bias for sensitivity estimates, the null hypothesis is less likely to be rejected. Since CCA is more biased than MICE, the power of CCA is more affected by these bias patterns. When data is MNAR, both CCA and MCAR reach a power of 1 for specificity estimates because both methods show large positive bias here.

The Monte Carlo Errors are small (Table 5), indicating a precise estimation of power.

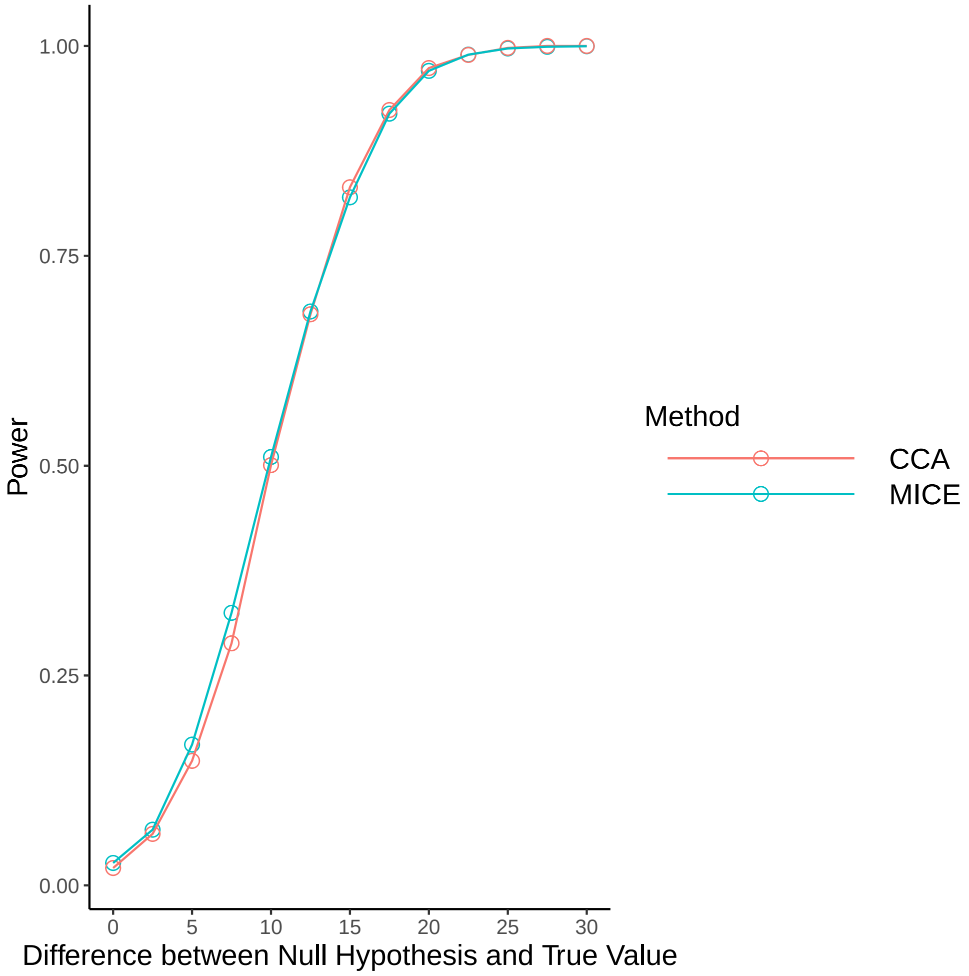

Fig. S37 Power of sensitivity estimates for CCA and MICE under MCAR

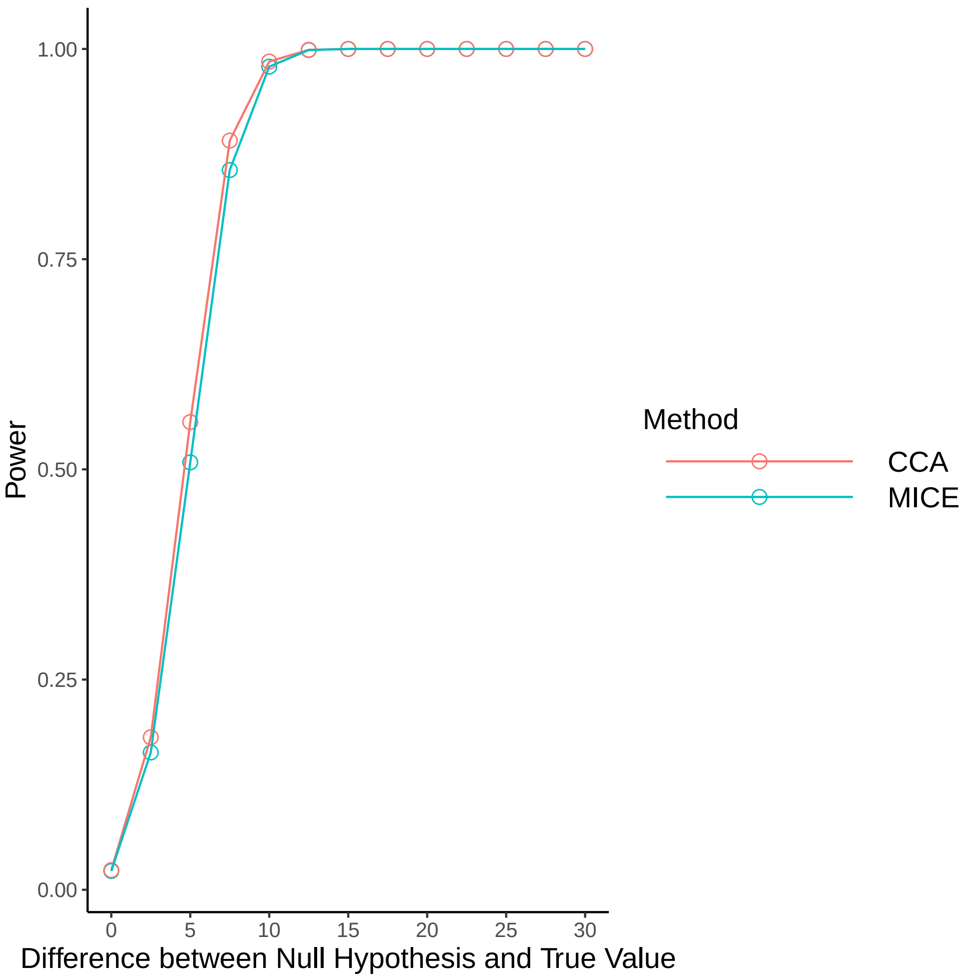

Fig. S38 Power of specificity estimates for CCA and MICE under MCAR

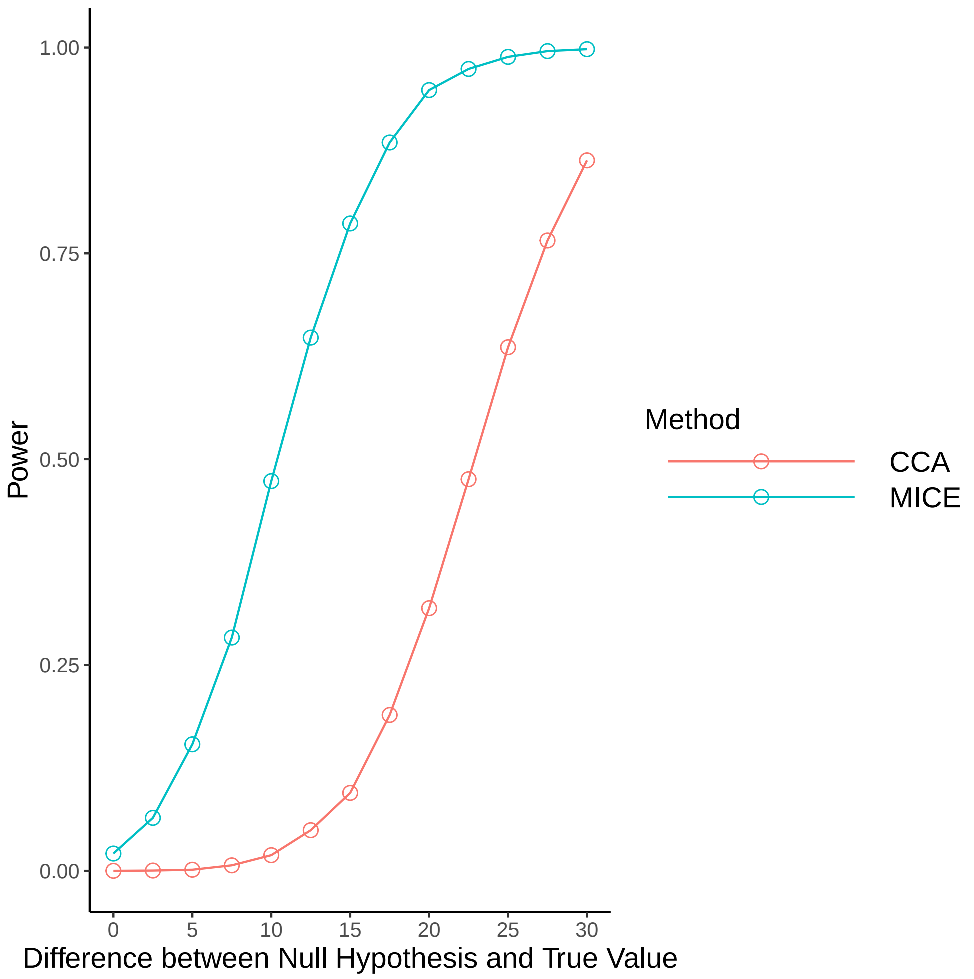

Fig. S39 Power of sensitivity estimates for CCA and MICE under MAR

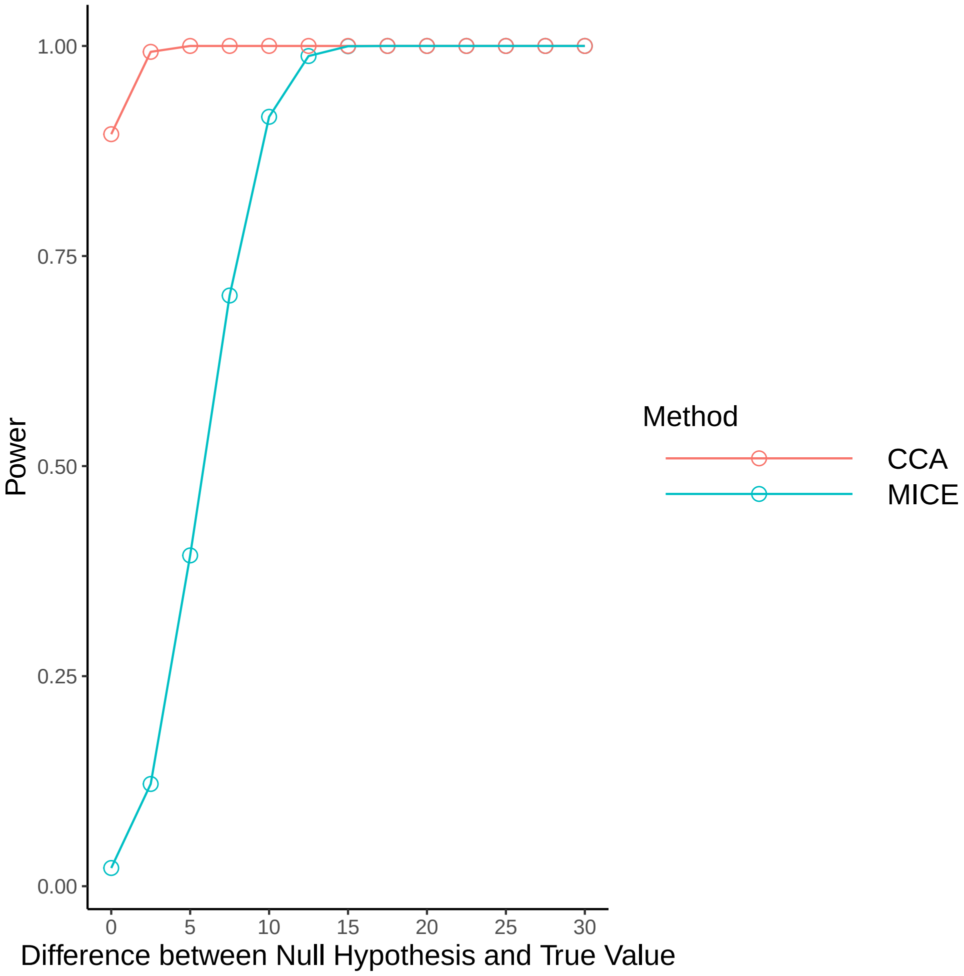

Fig. S40 Power of specificity estimates for CCA and MICE under MAR

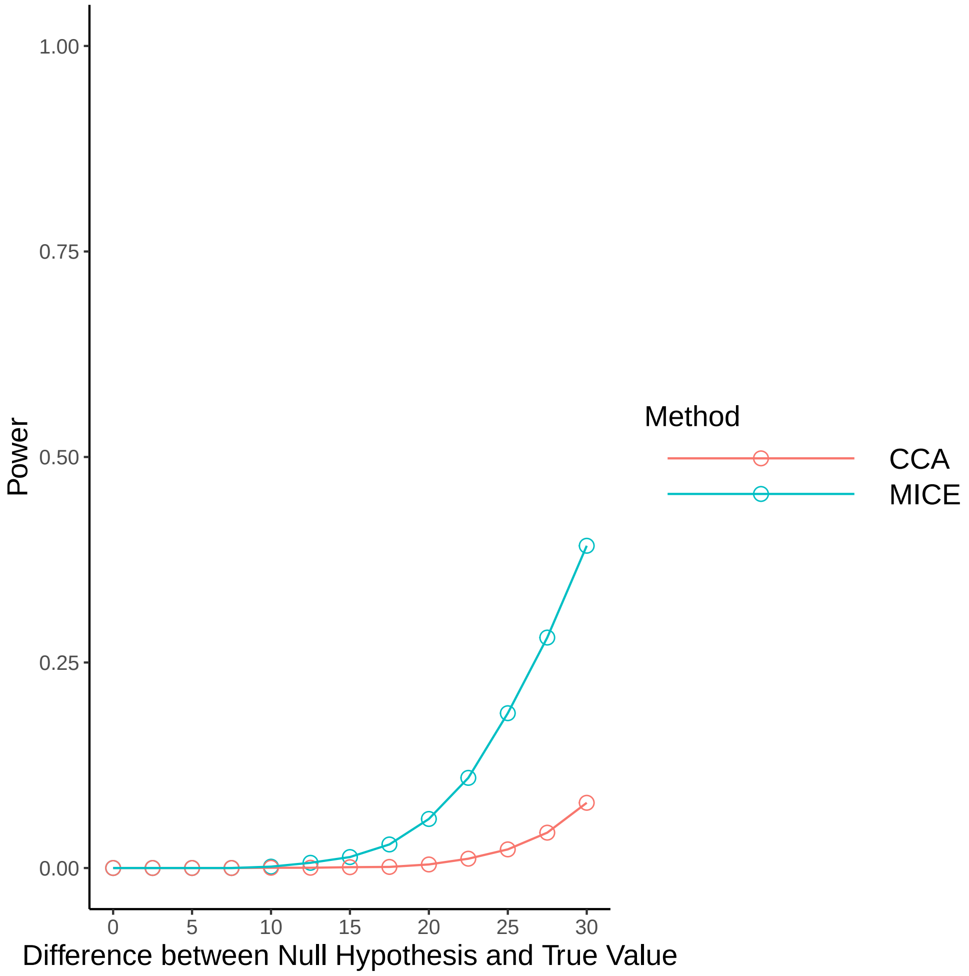

Fig. S41 Power of sensitivity estimates for CCA and MICE under MNAR

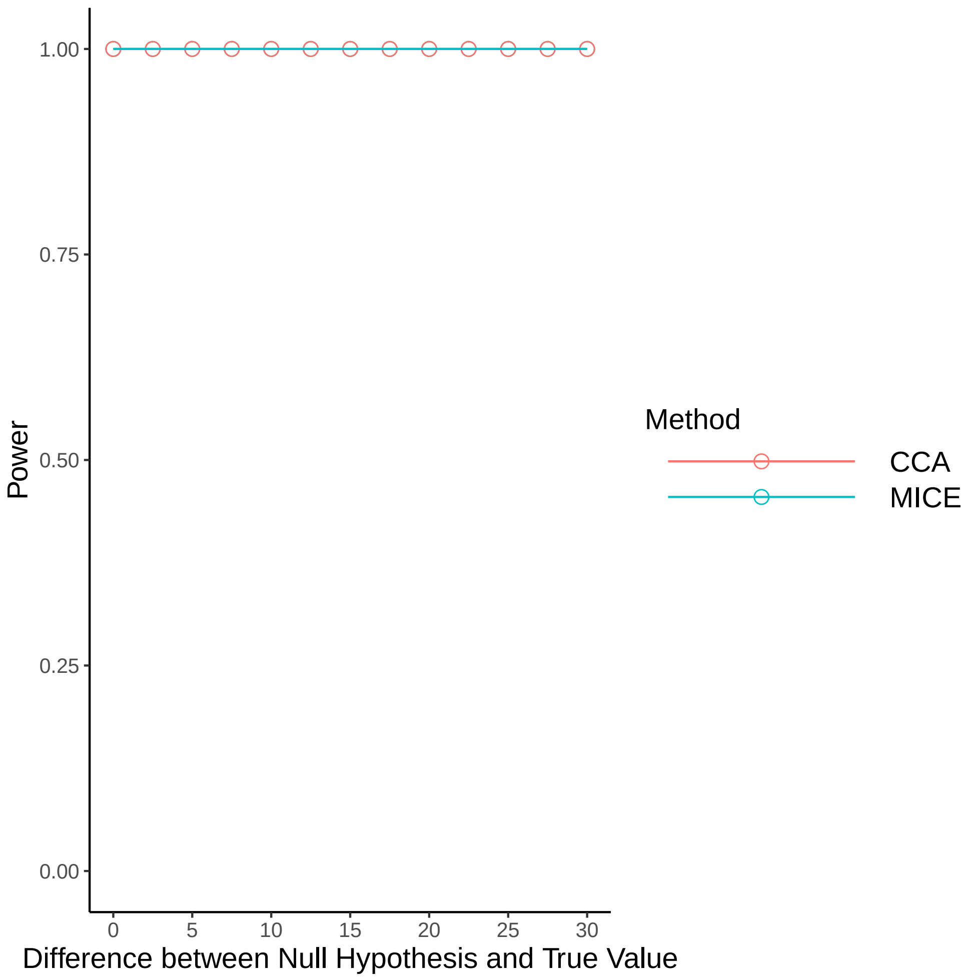

Fig. S42 Power of specificity estimates for CCA and MICE under MNAR

6 Context for the logit confidence interval

The delta method allows for an approximate variance for a (nonlinear) transformation of a random variable. If the random variable X is transformed by a nonlinear function f and evaluated at μ, the delta method can approximate the variance in the following way [1]:

$$var(f(X)) \approx [f'(\mu)]^2\sigma^2. \quad (1)$$

The derivative of the transformation function *logit* looks like this:

$$\frac{d}{dp}logit(p) = \frac{d}{dp}[log(p) - log(1-p)] = \frac{1}{p} - \frac{1}{1-p} = \frac{1}{p(1-p)}. \qquad (2)$$

If f is the *logit* transformation and the function is evaluated at the binomial proportion estimation $\hat{p}$, then the corresponding variance approximation

$$\left(\frac{d}{dp}logit(\hat{p})\right)^2 (\hat{p}(1-\hat{p})) = \frac{1}{\hat{p}(1-\hat{p})} \qquad (3)$$

is obtained with the standard error

$$\widehat{SE} = \frac{1}{n(\hat{p}(1-\hat{p}))}. \qquad (4)$$

From here it can clearly be seen how the confidence interval

$$logit^{-1}\left[log\left(\frac{p}{1-p}\right) \pm \frac{z_{\alpha/2}}{\sqrt{np(1-p)}}\right] \qquad (5)$$

is derived.

7 Confidence interval lengths

Table 8 Logit confidence interval lengths for specificity estimates

Imputation Method	Mean	Median	Minimum	Maximum
CCA	0.0688	0.0643	0.0085	0.1890
WC	0.0774	0.0729	0.0318	0.1258
RHD	0.0578	0.0543	0.0048	0.1258
MICE	0.0757	0.0701	0.0102	0.5099
MLMCAR	0.0595	0.0563	0.0051	1
MLMAR	0.0595	0.0563	0.0051	1
WLSMCAR	0.0595	0.0563	0.0051	1

Table 9 Wald confidence interval lengths for sensitivity estimates

Imputation Method	Mean	Median	Minimum	Maximum
CCA	0.1925	0.1654	0	1
WC	0.1509	0.1451	0	0.3099
RHD	0.1364	0.1268	0	0.3099
MICE	0.1747	0.1496	0	1
MLMCAR	0.1365	0.1262	0.00005	0.3099
MLMAR	0.1365	0.1262	0.00005	0.3099
WLSMCAR	0.1361	0.1265	0.00005	0.3099

Note. All upper confidence interval estimates that were larger than 1 have been set to 1.

Table 10 Wald confidence interval lengths for specificity estimates

Imputation Method	Mean	Median	Minimum	Maximum
CCA	0.0683	0.0636	0	0.1912
WC	0.0775	0.0730	0.0317	0.1265
RHD	0.0575	0.0538	0	0.1264
MICE	0.0752	0.0695	0	0.3193
MLMCAR	0.0591	0.0560	1.01734e-05	0.1260
MLMAR	0.0591	0.0560	1.01734e-05	0.1256
WLSMCAR	0.0591	0.0560	1.0168e-05	0.1260

Note. All upper confidence interval estimates that were larger than 1 have been set to 1.

8 Derivation of how sensitivity and specificity values were ensured

The distribution for the population without the target condition is $\mathcal{N}(0,1)$, while the distribution for the population with the target condition is $\mathcal{N}(\mu_1,1)$ with μ_1 being some mean yet to be determined.

To determine the specificity, we need a cut-off value c such that

$$Specificity = \Phi(c) \tag{6}$$

where Φ is the cumulative distribution function of the normal distribution.

The two distributions have the same shape and are only shifted by μ_1. Since this is the case, the false negative fraction, i.e. $1 - \mathit{Sensitivity}$ can be described in the following way:

$$1 - \mathit{Sensitivity} = \Phi(c - \mu_1). \tag{7}$$

Solving for μ_1 reveals

$$\mu_1 = c - \Phi^{-1}(1 - \mathit{Sensitivity}). \tag{8}$$

From 6 follows $c = \Phi^{-1}(Specificity)$, so finally

$$\mu_1 = \Phi^{-1}(Specificity) - \Phi^{-1}(1 - Sensitivity). \quad (9)$$

When specific sensitivity and specificity values need to be ensured for this model, a mean for the population with the target condition is chosen via $\mu_1 = \Phi^{-1}(True\ Specificity) - \Phi^{-1}(1 - True\ Sensitivity)$ and then $c = \Phi^{-1}(True\ Specificity)$ is specified as a cut-off value to dichotomize the continous data into index test results.

9 ampute function: weight equations for MAR and MNAR

9.1 MAR

$$wss_{MAR} = 0 \cdot Index\ test + 1 \cdot Covariate\ 1 + 1 \cdot Covariate\ 2 + 1 \cdot Covariate\ 3 + 1 \cdot Reference\ test \quad (10)$$

9.2 MNAR

$$wss_{MNAR} = 1 \cdot Index\ test + 0 \cdot Covariate\ 1 + 0 \cdot Covariate\ 2 + 0 \cdot Covariate\ 3 + 0 \cdot Reference\ test \quad (11)$$

References

[1] Ver Hoef, J.M.: Who invented the delta method? The American Statistician **66**(2), 124–127 (2012)
